# Facilitating safety evaluation in maternal immunization trials: a retrospective cohort study to assess pregnancy outcomes and events of interest in low-risk pregnancies in England

**DOI:** 10.1186/s12884-022-04769-x

**Published:** 2022-06-01

**Authors:** Megan Riley, Dimitra Lambrelli, Sophie Graham, Ouzama Henry, Andrea Sutherland, Alexander Schmidt, Nicola Sawalhi-Leckenby, Robert Donaldson, Sonia K. Stoszek

**Affiliations:** 1 GSK, 14200 Shady Grove Rd, Rockville, MD 20850 Washington, USA; 2 Evidera, 201 Talgarth Rd, Hammersmith, London, W6 8BJ, UK; 3 Moderna, Cambridge, MA USA; 4 Bill & Melinda Gates Medical Research Institute, Cambridge, MA USA

**Keywords:** Maternal vaccination, Pregnancy outcomes, Low-risk pregnancy, Background rate, Maternal immunization trial, Miscarriage, Preterm delivery, Pregnancy-related events of interest, Pharmacovigilance

## Abstract

**Background:**

Maternal characteristics like medical history and health-related risk factors can influence the incidence of pregnancy outcomes and pregnancy-related events of interest (EIs). Data on the incidence of these endpoints in low-risk pregnant women are needed for appropriate external safety comparisons in maternal immunization trials. To address this need, this study estimated the incidence proportions of pregnancy outcomes and pregnancy-related EIs in different pregnancy cohorts (including low-risk pregnancies) in England, contained in the Clinical Practice Research Datalink (CPRD) Pregnancy Register linked to Hospital Episode Statistics (HES) between 2005 and 2017.

**Methods:**

The incidence proportions of 7 pregnancy outcomes and 15 EIs were calculated for: (1) all pregnancies (AP) represented in the CPRD Pregnancy Register linked to HES (AP cohort; *N* = 298 155), (2) all pregnancies with a gestational age (GA) ≥ 24 weeks (AP24+ cohort; *N* = 208 328), and (3) low-risk pregnancies (LR cohort; *N* = 137 932) with a GA ≥ 24 weeks and no diagnosis of predefined high-risk medical conditions until 24 weeks GA.

**Results:**

Miscarriage was the most common adverse pregnancy outcome in the AP cohort (1 379.5 per 10 000 pregnancies) but could not be assessed in the other cohorts because these only included pregnancies with a GA ≥ 24 weeks, and miscarriages with GA ≥ 24 weeks were reclassified as stillbirths. Preterm delivery (< 37 weeks GA) was the most common adverse pregnancy outcome in the AP24+ and LR cohorts (742.9 and 680.0 per 10 000 pregnancies, respectively). Focusing on the cohorts with a GA ≥ 24 weeks, the most common pregnancy-related EIs in the AP24+ and LR cohorts were fetal/perinatal distress or asphyxia (1 824.3 and 1 833.0 per 10 000 pregnancies), vaginal/intrauterine hemorrhage (799.2 and 729.0 per 10 000 pregnancies), and labor protraction/arrest disorders (752.4 and 774.5 per 10 000 pregnancies).

**Conclusions:**

This study generated incidence proportions of pregnancy outcomes and pregnancy-related EIs from the CPRD for different pregnancy cohorts, including low-risk pregnancies. The reported incidence proportions of pregnancy outcomes and pregnancy-related EIs are largely consistent with external estimates. These results may facilitate the interpretation of safety data from maternal immunization trials and the safety monitoring of maternal vaccines. They may also be of interest for any intervention studied in populations of pregnant women.

**Supplementary Information:**

The online version contains supplementary material available at 10.1186/s12884-022-04769-x.

## Background

Maternal immunization has the potential to reduce the burden of infectious diseases in infants via the transplacental transfer of protective maternal antibodies, which persist after birth and help protect infants from infection in their first months of life [1, 2]. Maternal immunization may also provide additional benefits by preventing infectious diseases in pregnant women, potentially reducing adverse pregnancy and infant outcomes associated with maternal infections [3, 4].

Currently, immunization to protect against influenza, tetanus, and pertussis is recommended during pregnancy by the World Health Organization [5–7], and many individual countries including the United States (US) and the United Kingdom (UK) recommend that pregnant women receive influenza and pertussis vaccinations [8, 9]. In addition to licensed vaccines that are recommended during pregnancy, maternal vaccine candidates are being developed for the prevention of infections in mothers and their offspring, including vaccines against respiratory syncytial virus (RSV) and group B streptococcus (GBS) infections [10–12]. Group B streptococcus is a leading cause of neonatal sepsis and meningitis, with the highest incidence during the first 3 months of life [13, 14], and RSV causes respiratory tract infections that may be severe in infants and young children, with the highest hospitalization rate in infants < 1 year old [15, 16]. In the long term, these infants are more likely to suffer from recurrent respiratory symptoms and asthma [13].

Vaccines routinely recommended during pregnancy (e.g., inactivated influenza and tetanus-reduced-antigen-content diphtheria-acellular pertussis vaccines) were originally licensed based on data generated in non-pregnant populations. By contrast, maternal vaccine candidates against RSV and GBS aim to demonstrate safety in vaccinated pregnant women and their offspring, and efficacy (or immunogenicity as proxy) in the infants for their primary indication [2, 10]. The pregnancy-specific vaccine development approach requires the conduct of large-scale maternal immunization trials during clinical development. Prior to conducting such trials, it is critical to understand the background rates of pregnancy outcomes and pregnancy-related events of interest (EIs) in specific populations to facilitate the interpretation of these outcomes and EIs after maternal vaccination [10].

Previous studies have demonstrated that certain maternal characteristics, such as prior medical history and health-related risk factors, are associated with adverse pregnancy outcomes (e.g., stillbirth and preterm delivery) and pregnancy-related EIs (e.g., gestational diabetes and hypertension) [17–22]. Past studies have also demonstrated an increased risk of adverse pregnancy outcomes and pregnancy-related EIs in women from low socioeconomic backgrounds relative to those from high socioeconomic backgrounds [23–25]. Data describing the incidence of pregnancy outcomes and EIs in women with low-risk pregnancies (i.e., pregnancies without high-risk conditions expected to increase the risk of pregnancy complications) approaching the end of the second trimester (e.g., as of 24 weeks gestational age [GA]) are limited but needed as external reference in maternal immunization trial safety comparisons [26–28]. In addition, data are lacking to quantify pregnancy outcomes and pregnancy-related EIs in all pregnant women once they reach 24 weeks GA. We addressed this knowledge gap by conducting a retrospective, observational cohort study using the UK Clinical Practice Research Datalink (CPRD) with data linked to the Pregnancy Register and Hospital Episode Statistics (HES). The Pregnancy Register was created by an algorithm that identifies all pregnancies (and details on timing and outcomes) among women aged 11–49 years in CPRD GOLD, one of CPRD’s primary care databases [29, 30].

**Fig. 1:**
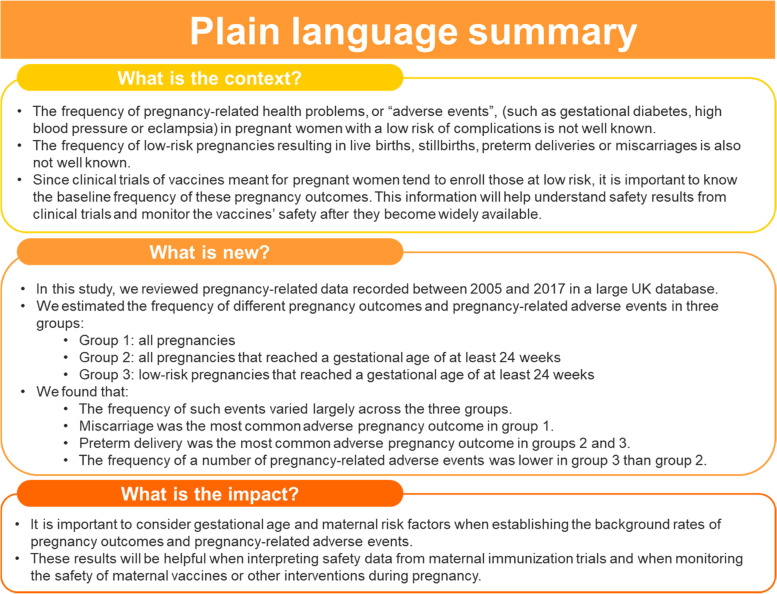
Plain language summary

The objective of this study was to estimate the incidence proportions of pregnancy outcomes and pregnancy-related EIs in three cohorts of pregnant women identified in the CPRD Pregnancy Register linked to HES: (1) all pregnancies, (2) all pregnancies with a GA ≥ 24 weeks, and (3) low-risk pregnancies with a GA ≥ 24 weeks. The study also examined adverse outcomes in liveborn infants from women in the different pregnancy cohorts with Mother-Baby Link. These data are published in an accompanying paper [31]. A plain language summary is provided in Fig. 1.

## Methods

The protocol of this retrospective observational cohort study was approved by the Independent Scientific Advisory Committee (ISAC) for research involving CPRD data (protocol no. 18_144RA) and has been made available to the journal reviewers.

### Data sources

At the time of data extraction (September 2018), CPRD GOLD contained longitudinal primary care data from 745 real-world clinical practices in the UK, with 269 currently contributing practices, 40% of which were located in England. CPRD GOLD includes over 15 million patient lives, with over 2 million registered and active patients (covering 3.5% of the UK population). CPRD primary care data are representative of the UK population with respect to age, gender, and ethnicity [32]. This study used data from the Pregnancy Register liked to HES, Office for National Statistics (ONS) mortality data, and the Index of Multiple Deprivation (IMD).

The Pregnancy Register uses a validated algorithm that identifies pregnancy episodes in CPRD GOLD. The algorithm uses all available data to identify the timing (start, end, and trimester dates), outcome, and other associated details of each pregnancy episode. As each pregnancy episode is included in the Pregnancy Register as a separate event, more than one pregnancy per woman may be included in the Pregnancy Register over time [29, 33]. A previously published study showed that the internal and external validation of the algorithm had a 91% sensitivity for identifying and dating hospital deliveries and a 77% sensitivity for hospital-based early pregnancy losses. For miscarriages, the rates were comparable to external sources while for termination and live births, lower rates were observed in the Pregnancy Register. Further validation studies are ongoing [29]. Data linkage to HES provides diagnostic secondary care records, including inpatient and outpatient records, for England only [30] (thus restricting the analysis to pregnancies in England which were linkable to HES). ONS mortality data provide information on the date and cause of all deaths recorded in England and Wales [30]. The IMD is an area-based measure of relative deprivation that ranks small areas in England on the patient level as a proxy for socioeconomic status. Data are provided in the form of quintiles of deprivation, from 1 (least deprived) to 5 (most deprived) [30].

### Study period

**Fig. 2:**
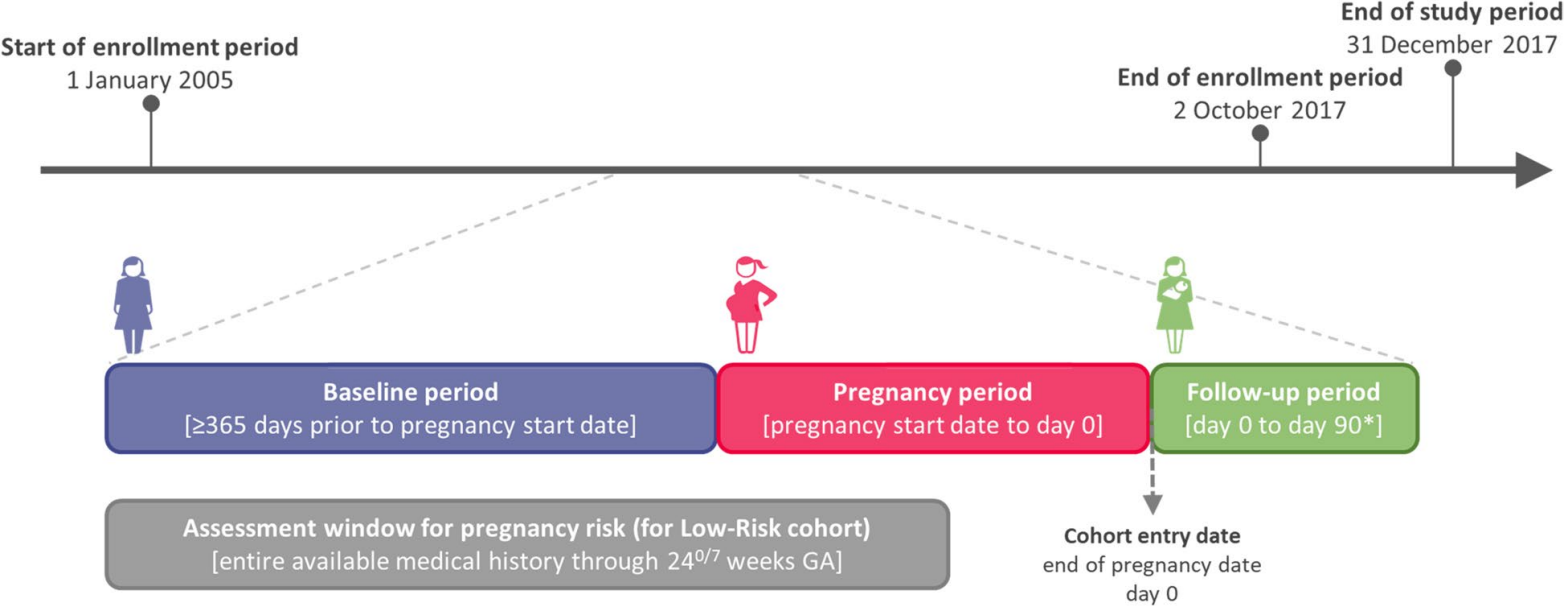
Overview of the study period. *GA*, gestational age. *A minimum of 90 days of active registration after the pregnancy end date was required for women to be enrolled except if the woman died during this 90-day period

The study included pregnancies in the CPRD Pregnancy Register with linkage to HES and a pregnancy end date between 1 January 2005 and 31 December 2017. To increase outcome ascertainment, a 90-day follow-up period after the pregnancy end date was required (unless the woman died before the end of this period). Therefore, pregnancies with an end date up until 2 October 2017 were included in the study cohorts. In addition, continuous active registration starting from at least 365 days before the start of pregnancy was required to assess for high-risk factors at baseline, which were used to establish the Low-Risk (LR) cohort. Figure 2 provides a visualization of each phase within the study period.

### Study population

To generate a range of background rates for each endpoint, three study cohorts were designed that might be expected in maternal immunization clinical trials, depending on the strictness of the inclusion/exclusion criteria and the timing of vaccination.

The All Pregnancies (AP) cohort included all pregnancies recorded in the CPRD Pregnancy Register between 1 January 2005 and 31 December 2017 with linkage to HES, ≥ 365 days of continuous active registration prior to the pregnancy start date, ≥ 90 days of active registration following the pregnancy end date (unless the woman died before the end of this period), acceptable data quality (i.e., whether the patient met certain quality standards based on a valid age and gender, recording of events and registration status [32]), and a maternal age ≥ 18 to ≤ 45 years on the pregnancy end date. Pregnancy episodes associated with multiple births (e.g., twins, triplets) and with an unknown outcome were excluded as this study was designed to reflect the population expected to be enrolled in maternal immunization trials (Additional file 1). For live births with a GA < 20 weeks or > 44 weeks, the GA was recategorized to missing.

The All Pregnancies ≥ 24 weeks GA cohort (AP24+ cohort) was a subgroup of the AP cohort, including pregnancies with a GA ≥ 24^0/7^ weeks. This subgroup excluded all women with a recorded GA < 24^0/7^ weeks (calculated using the variable in the Pregnancy Register: “gestdays” < 168 days) and served as a GA-based descriptive comparator group for the LR cohort. The GA cut-off of 24 weeks was selected as it is the same as the one chosen in previous GBS maternal immunization trials [26–28] and falls within the timeframe of recommended maternal pertussis immunization in several countries [34].

The LR cohort included pregnancies from the AP24+ cohort without diagnosis of select high-risk medical conditions or procedures in the woman’s medical history (including all available medical history prior to start of pregnancy through 24^0/7^ weeks GA). See Additional files 2 and 3 for additional information on the eligibility criteria of the LR cohort, including the codes used to identify the exclusion criteria. The high-risk medical conditions and procedures determined as exclusion criteria for the LR cohort were selected based on potential exclusion criteria for maternal immunization trials.

Additional cohorts were defined with linkage to the Mother-Baby Link to assess adverse infant outcomes, as described in the accompanying paper [31].

### Study endpoints and variables

The selection of study endpoints was guided by the standardized case definitions established by the Brighton Collaboration and Global Alignment of Immunization Safety Assessment (GAIA) project for use in maternal immunization trials. The aim of these standardized case definitions is to achieve global alignment in the case definitions of safety outcomes in clinical trials enrolling pregnant women. This harmonization will enable comparison of safety data between and among maternal immunization trials [35, 36]. To ensure the broad applicability of study results, the case definitions of pregnancy outcomes and pregnancy-related EIs were manually aligned with those provided by the Brighton Collaboration and GAIA wherever possible. However, the exact application of GAIA definitions was challenging because laboratory results, procedure results, and medication prescribed during a hospital stay are underreported in the CPRD and linked databases. Furthermore, GAIA case definitions were not available for all study endpoints. Therefore, diagnostic coding was used (Read codes in CPRD GOLD and International Classification of Diseases, 10^th^ Revision [ICD-10] codes in HES). Additional files 4 and 5 show each endpoint with the corresponding GAIA definition and diagnostic codes.

### Pregnancy outcomes

Table 1 lists the pregnancy outcomes assessed in the study (live birth and adverse pregnancy outcomes), as recorded in the Pregnancy Register between the pregnancy start and end dates. Of note, miscarriages with a GA > 24 weeks were reclassified as stillbirths. The identification algorithms and codes are listed in Additional files 4 and 5.

**Table 1:** Pregnancy outcomes and pregnancy-related events of interest

**Pregnancy outcomes**
***Assessed from start of pregnancy to pregnancy end date***
Live birth
Preterm delivery (or record of live birth occurring < 37 weeks GA)
Fetal death/stillbirth (loss at or after 22 weeks GA)
Miscarriage ^a^
Termination (elective or therapeutic)
Miscarriage or termination (composite endpoint)
Ectopic pregnancy
**Pregnancy-related events of interest**
***Assessed from start of pregnancy to 90 days post-pregnancy end date***
Maternal sepsis
Vaginal or intrauterine hemorrhage
Pre-eclampsia
Eclampsia
Pregnancy-related hypertension
Liver or biliary disease
***Assessed from start of pregnancy to pregnancy end date***
Premature or preterm labor
Labor protraction or arrest disorders
Oligohydramnios
Polyhydramnios
Intrauterine growth restriction or poor fetal growth
Gestational diabetes mellitus
***Assessed during period as stated***
Maternal death *(from start of pregnancy to 42 days after delivery date)*
Preterm premature rupture of membranes *(from start of pregnancy until 37 weeks GA)*
Fetal/perinatal distress or asphyxia *(from start of pregnancy to 7 days after delivery)*

*GA* gestational age

See Additional file 5 for codes used to identify these outcomes

^a^ Miscarriages with a GA > 24 weeks were reclassified as stillbirths

### Pregnancy-related EIs

Table 1 provides the list of pregnancy-related EIs assessed in the study along with the associated timeframe for which each was assessed. All pregnancy-related EIs, with the exception of maternal death, were identified based on Read codes in CPRD or ICD-10 codes in HES (Additional files 4 and 5). Maternal death was identified based on the date of death in CPRD (Additional file 6) or ONS (Additional files 4 and 5). The date from ONS was used if conflicting information was reported.

### Variables

The following variables were assessed in the study: contraception use, smoking status and alcohol intake in the 365 days before the pregnancy start date (data not shown); and maternal age at pregnancy start, calendar year at pregnancy start, number of pregnancies in the study period (data not shown), ethnicity, quintile of deprivation in IMD, and pregnancy number (data not shown) (Additional files 7 and 8). These variables were selected as being potential risk factors for the evaluated pregnancy outcomes and pregnancy-related EIs.

### Statistical analyses

Analyses were conducted using SAS software version 9.4 (SAS Institute Inc., Cary, NC, US). No hypothesis testing was performed in this descriptive study. Potential differences between groups were based on non-overlapping 95% confidence intervals (CIs). Feasibility counts during protocol development indicated that the sample size obtained from the databases would provide sufficient precision for the descriptive purpose of the study. Standard data management practices were performed on the databases (i.e., the initial cohort selection process, subsequent revisions of the selection process and statistical analyses were reviewed by the Data Analyst, the Quality Control Analyst and the Principal Investigator).

Descriptive analyses of demographic characteristics of all pregnancy cohorts were conducted, including number and proportion for categorical variables, and mean, standard deviation, median, interquartile range (IQR), and minimum and maximum values for continuous variables. Within each cohort, the incidence proportion of each study endpoint was calculated as follows:

$$\frac{\mathrm{Number\ of\ new\ cases\ of\ study\ outcomes\ or\ EI\ in\ the\ period\ of\ interest}}{\mathrm{Number\ of\ pregnancies\ identified\ in\ CPRD\ in\ the\ period\ of\ interest}}$$

The incidence proportions and 95% CIs of the study endpoints were calculated for every 10 000 pregnancies. Due to the study design and use of the Pregnancy Register as a data source, women were permitted to contribute more than one sequential pregnancy to the dataset over time. To account for clustering in the data due to the non-independent nature of sequential pregnancies included in the dataset for the same woman, the 95% CIs of incidence proportions were estimated via a generalized estimating equation model [37]. Missing values in the data were identified but not replaced, as assuming a nature of missing at random. To maintain confidentiality and individual data anonymization, data were provided only if at least five cases were observed for a given strata or subgroup. Each study endpoint was presented for the entire study period. Exploratory analyses to stratify each study endpoint by calendar year of pregnancy start date, maternal age at start of pregnancy (18–24, 25–29, 30–34, 35–39, and 40–45 years of age), ethnicity (white, Asian, black, mixed, other, and unknown), and IMD quintile (1 [least deprived]–5 [most deprived]) were also conducted.

## Results

### Sample selection and cohort description

**Fig. 3:**
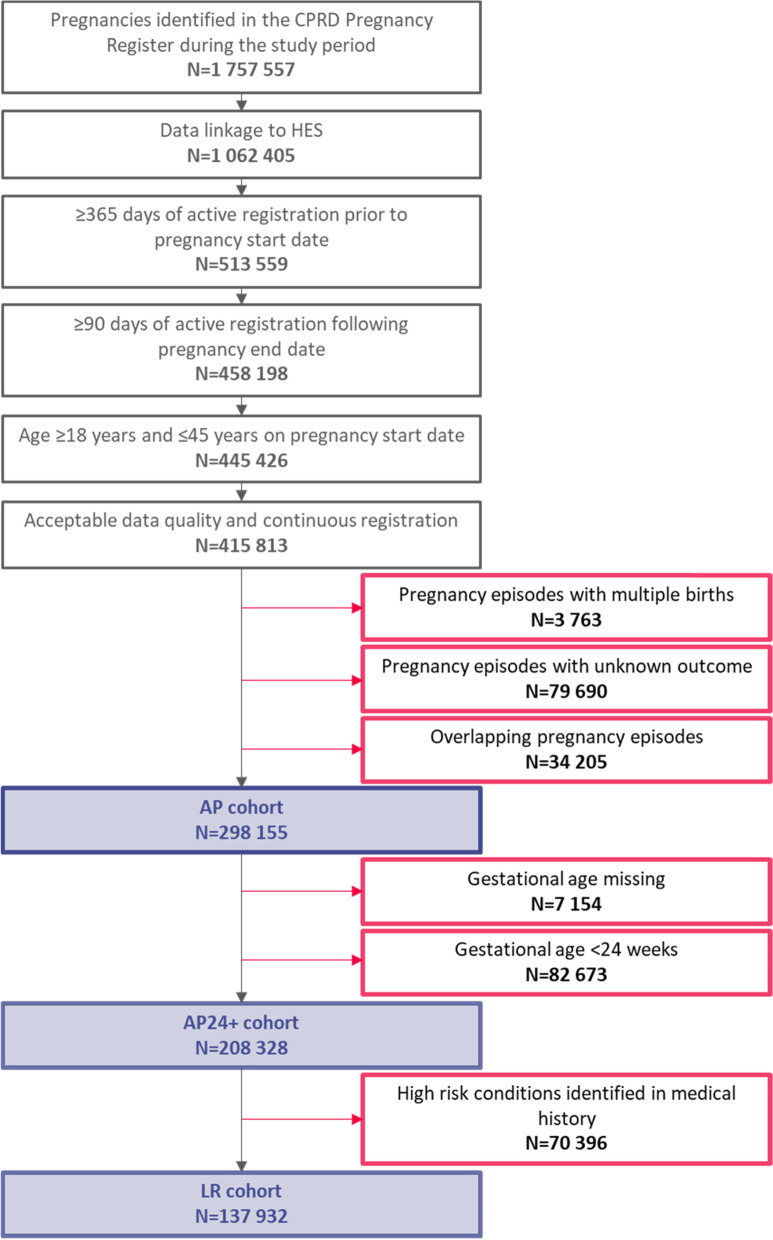
Cohort selection flow chart. *AP*, All Pregnancies; *AP24+*, All Pregnancies with gestational age ≥24 weeks; *LR*, Low-Risk pregnancies; *CPRD*, Clinical Practice Research Datalink; *HES*, Hospital Episode Statistics; *N*, number of pregnancies in the corresponding group/category

**Fig. 4:**
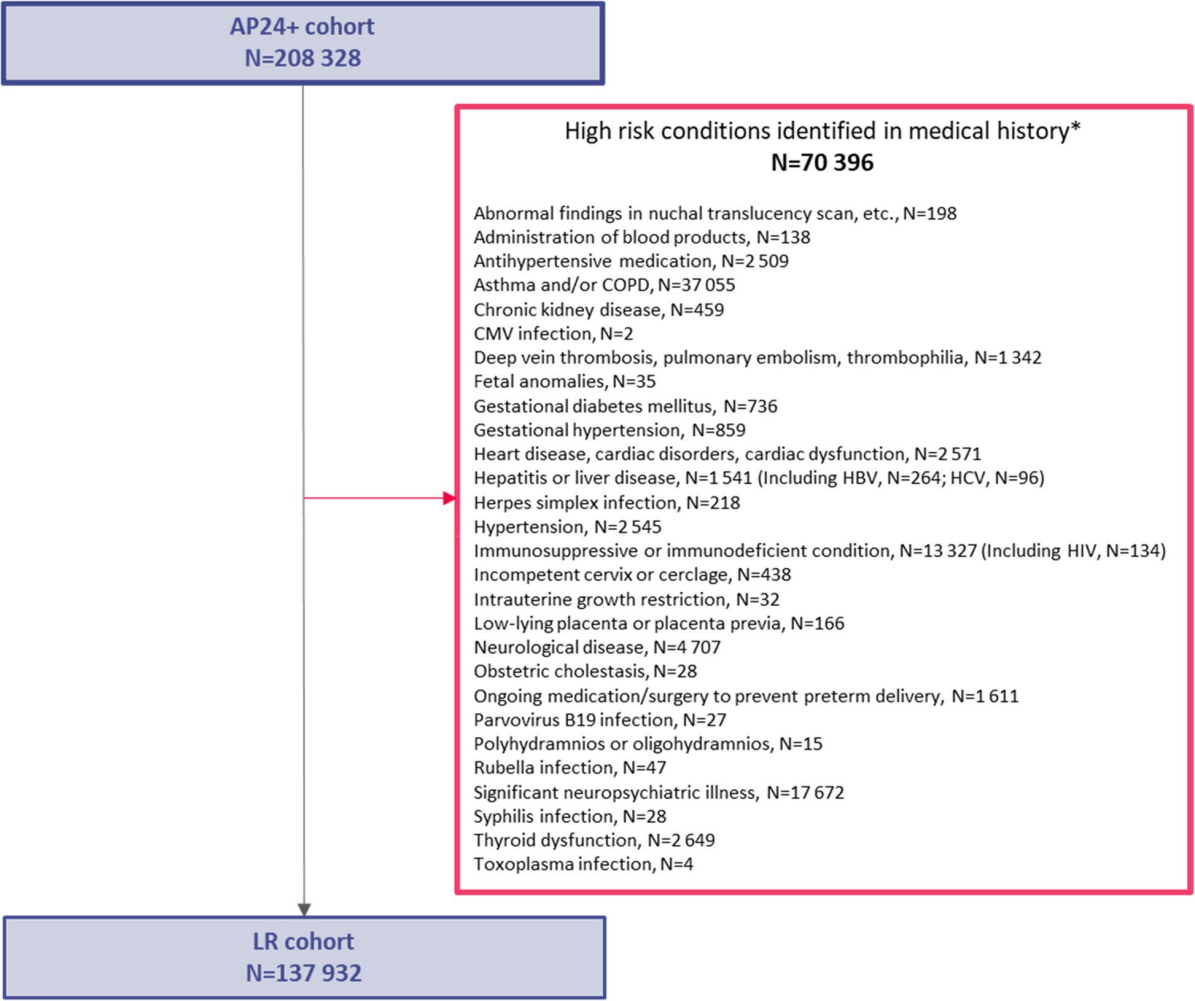
High-risk medical conditions or procedures in medical history* leading to exclusion from LR cohort. *AP*, All Pregnancies; *AP24+* , All Pregnancies with gestational age ≥ 24 weeks; *LR*, Low-Risk pregnancies; *N*, number of pregnancies in the corresponding group/category; *CMV*, cytomegalovirus; *COPD*, chronic obstructive pulmonary disorder; *HBV*, hepatitis B virus; *HCV*, hepatitis C virus; *HIV*, human immunodeficiency virus. *All available medical history prior to start of pregnancy through 24^0/7^ weeks gestational age (see Additional file 2 for algorithms and assessment periods)

We identified 1 757 557 pregnancies across the study period, of which 1 062 405 (60.4%) were linked to HES. Once selection criteria were applied, 298 155 pregnancies were ultimately included in the AP cohort, of which 208 328 (69.9%) had a recorded GA ≥ 24 weeks and were included in the AP24+ cohort (Fig. 3). Of the pregnancies in the AP24+ cohort, 137 932 (66.2%) were included in the LR cohort. Figure 3 provides the disposition of subjects within cohorts, and Fig. 4 provides an overview of the pregnancies excluded from the LR cohort by individual exclusion criteria.

### Demographic characteristics

The median duration of pregnancy in the AP cohort was shorter with a wider IQR compared to the AP24+ and LR cohorts (Table 2). The median age of women at the start of pregnancy was 30 years for all three cohorts (Table 2). By age category, the highest proportion of women were 30–34 years of age at the start of pregnancy (around 30% for all cohorts, Table 2). Most women were white, and women within each cohort were evenly distributed across the five IMD quartiles.

**Table 2:** Demographics and baseline characteristics by study cohort

Parameter	AP cohort *N* = 298 155	AP24+ cohort *N* = 208 328	LR cohort *N* = 137 932
**Duration of pregnancy, days**			
Mean (SD)	221 (89.3)	276 (13.9)	277 (13.4)
Median (IQR)	273 (84.0–280.0)	280 (273.0–280.0)	280 (273.0–281.0)
**Maternal age, years**			
Mean (SD)	30 (6.2)	30 (5.8)	30 (5.7)
Median (IQR)	30 (25.0–35.0)	30 (26.0–34.0)	30 (26.0–34.0)
**Maternal age group, n (%)**			
18–24 years	64 282 (21.6)	40 102 (19.2)	25 770 (18.7)
25–29 years	73 199 (24.6)	54 429 (26.1)	35 746 (25.9)
30–34 years	85 923 (28.8)	65 489 (31.4)	44 713 (32.4)
35–39 years	56 947 (19.1)	39 366 (18.9)	26 076 (18.9)
40–45 years	17 804 (6.0)	8 942 (4.3)	5 627 (4.1)
**Ethnicity, n (%)**			
White	252 166 (84.6)	178 527 (85.7)	115 810 (84.0)
Asian	16 757 (5.6)	12 054 (5.8)	8 918 (6.5)
Black	8 821 (3.0)	5 672 (2.7)	4 071 (3.0)
Mixed	3 528 (1.2)	2 320 (1.1)	1 518 (1.1)
Other	6 277 (2.1)	4 360 (2.1)	3 342 (2.4)
Unknown	10 606 (3.6)	5 395 (2.6)	4 273 (3.1)
**Index of Multiple Deprivation, n (%)**			
1 (least deprived)	61 945 (20.8)	44 577 (21.4)	30 724 (22.3)
2	58 360 (19.6)	41 240 (19.8)	27 772 (20.1)
3	58 758 (19.7)	40 881 (19.6)	27 328 (19.8)
4	59 246 (19.9)	40 908 (19.6)	26 604 (19.3)
5 (most deprived)	59 612 (20.0)	40 573 (19.5)	25 415 (18.4)
Missing	234 (0.1)	149 (0.1)	89 (0.1)
**Year of pregnancy start, n (%)**			
2004 ^a^	16 027 (5.4)	14 041 (6.7)	10 040 (7.3)
2005	27 609 (9.3)	19 220 (9.2)	13 456 (9.8)
2006	28 221 (9.5)	19 706 (9.5)	13 527 (9.8)
2007	29 076 (9.8)	20 268 (9.7)	13 830 (10.0)
2008	29 761 (10.0)	20 382 (9.8)	13 709 (9.9)
2009	29 342 (9.8)	20 125 (9.7)	13 421 (9.7)
2010	27 983 (9.4)	19 479 (9.4)	12 763 (9.3)
2011	27 209 (9.1)	19 073 (9.2)	12 349 (9.0)
2012	24 661 (8.3)	16 888 (8.1)	10 688 (7.7)
2013	20 812 (7.0)	14 269 (6.8)	8 982 (6.5)
2014	16 166 (5.4)	10 959 (5.3)	6 697 (4.9)
2015	11 514 (3.9)	7 903 (3.8)	4 856 (3.5)
2016	8 580 (2.9)	5 954 (2.9)	3 589 (2.6)
2017 ^a^	1 194 (0.4)	61 (0.0)	25 (0.0)

*AP* All Pregnancies, *AP24*+ All Pregnancies with gestational age ≥ 24 weeks, *IQR* interquartile range, *LR* Low-Risk pregnancies, *n* number of pregnancies in the specified category, *N* number of pregnancies included in the analysis in each cohort, *SD* standard deviation

^a^ Because the study start date was 1 January 2005, the number of pregnancies reported as starting in 2004 includes only those which began in the last 9 months of 2004 (if full term, for example). Conversely, because the study period end date was 31 December 2017 (with pregnancy end date up until 2 October 2017, as there was a requirement for at least a 90-day follow-up after the pregnancy end date), the number of pregnancies reported as starting in 2017 includes only those which began in the first month of 2017 (if full term, for example)

Between 2005 and 2017, the number and proportion of pregnancies identified generally decreased by calendar year of pregnancy start date across all cohorts, particularly from 2013 onward (Table 2).

### Pregnancy outcomes

Live birth was the most common pregnancy outcome across all cohorts (Table 3). In the AP cohort, which included pregnancies of any GA, 7 197.3 per 10 000 pregnancies resulted in live births. In the AP24+ and LR cohorts, which only included pregnancies with a GA ≥ 24 weeks, 9 944.7 and 9 949.4 pregnancies per 10 000 resulted in live births, respectively. Preterm delivery occurred less frequently in the AP cohort (534.3 per 10 000 pregnancies, Table 3) than in the AP24+ and LR cohorts; the incidence proportion of preterm delivery was higher in the AP24+ cohort (742.9 per 10 000 pregnancies) than the LR cohort (680.0 per 10 000 pregnancies, Table 3).

**Table 3:** Incidence proportions of pregnancy outcomes by study cohort for the entire study period

	**AP cohort *N* = 298 155**		**AP24+ cohort *N* = 208 328**		**LR cohort *N* = 137 932**	
Pregnancy outcome	**n**	**Incidence/10 000 (95% CI)**	**n**	**Incidence/10 000 (95% CI)**	**n**	**Incidence/10 000 (95% CI)**
Live birth	214 592	7 197.3 (7 181.2–7 213.4)	207 176	9 944.7 (9 941.7–9 947.6)	137 234	9 949.4 (9 945.7–9 952.9)
Preterm delivery	15 931	534.3 (526.0–542.7)	15 476	742.9 (731.3–754.5)	9 380	680.0 (666.5–693.8)
Stillbirth	1 239	41.6 (39.5–43.7)	1 042	50.0 (47.3–52.9)	637	46.2 (42.9–49.7)
Miscarriage	41 129	1 379.5 (1 367.6–1 391.4)	0*	-	0 ^a^	-
Termination	15 590	522.9 (514.7–531.2)	110	5.3 (4.4–6.3)	61	4.4 (3.4–5.6)
Miscarriage or termination (composite endpoint)	22 609	758.3 (748.3–768.4)	0 ^a^	-	0 ^a^	-
Ectopic pregnancy	2 996	100.5 (97.0–104.1)	0 ^a^	-	0 ^a^	-

*AP* All Pregnancies, *AP24*+ All Pregnancies with gestational age ≥ 24 weeks, *CI* confidence interval, *Incidence* incidence proportion per 10 000 pregnancies, *LR* Low-Risk pregnancies, *n* number of pregnancies belonging to the specified category, *N* number of pregnancies included in the analysis in each cohort

^a^ Note the AP24+ and LR cohorts were limited to gestational age ≥ 24 weeks. Therefore, miscarriages and ectopic pregnancies could not be identified because miscarriages with gestational age > 24 weeks were reclassified as stillbirths and ectopic pregnancies occur prior to 24 weeks

Stillbirth was relatively rare within all cohorts at ≤ 50.0 stillbirths per 10 000 pregnancies (Table 3). Miscarriage was the most common adverse pregnancy outcome in the AP cohort (1 379.5 per 10 000 pregnancies). It could not be assessed in the AP24+ and LR cohorts because in our study, miscarriages with a GA > 24 weeks were reclassified as stillbirths (Table 3). Likewise, the pregnancy outcomes of miscarriage or termination (composite endpoint) and ectopic pregnancy (which is also expected to occur prior to 24 weeks GA) could not be assessed in the AP24+ and LR cohorts. For termination, a very low incidence proportion was observed for the AP24+ and LR cohorts (5.3 and 4.4 per 10 000 pregnancies, respectively) relative to the AP cohort (522.9 per 10 000 pregnancies, Table 3).

### Pregnancy-related events of interest

Across all cohorts, the most common pregnancy-related EIs were fetal/perinatal distress or asphyxia (1 318.7, 1 824.3, and 1 833.0 per 10 000 pregnancies in the AP, AP24+ , and LR cohorts, respectively), followed by vaginal or intrauterine hemorrhage (697.4, 799.2, and 729.0 per 10 000 pregnancies) and labor protraction/arrest disorders (541.6, 752.4, and 774.5 per 10 000 pregnancies) (Table 4).

**Table 4:** Incidence proportions of pregnancy-related events of interest by study cohort for the entire study period

	**AP cohort *N* = 298 155**		**AP24+ cohort *N* = 208 328**		**LR cohort *N* = 137 932**	
	**n**	**Incidence/10 000 (95% CI)**	**n**	**Incidence/10 000 (95% CI)**	**n**	**Incidence/10 000 (95% CI)**
**Assessed from the start of pregnancy to 90 days post-pregnancy end date**						
Maternal sepsis	220	7.4 (6.4–8.4)	174	8.4 (7.1–9.7)	103	7.5 (6.1–9.1)
Vaginal or intrauterine hemorrhage	20 794	697.4 (688.2–706.7)	16 649	799.2 (787.4–811.1)	10 055	729.0 (715.1–743.0)
Pre-eclampsia	4 581	153.6 (149.0–158.4)	4 340	208.3 (202.0–214.8)	2 558	185.5 (178.1–193.0)
Eclampsia	224	7.5 (6.5–8.6)	213	10.2 (8.9–11.7)	114	8.3 (6.8–10.0)
Pregnancy-related hypertension	1 175	39.4 (37.1–41.8)	1 114	53.5 (50.3–56.8)	556	40.3 (36.9–43.9)
Liver or biliary disease	36	1.2 (0.8–1.7)	33	1.6 (1.1–2.3)	7	0.5 (0.2–1.1)
**Assessed from the start of pregnancy to pregnancy end date**						
Premature/preterm labor	6 417	215.2 (209.9–220.6)	6 094	292.5 (285.2–300.0)	3 590	260.3 (251.8–269.0)
Labor protraction/arrest disorders	16 148	541.6 (533.2–550.1)	15 675	752.4 (740.9–764.1)	10 683	774.5 (760.2–789.1)
Oligohydramnios	1 552	52.1 (49.5–54.7)	1 463	70.2 (66.6–73.9)	827	60.0 (55.9–64.2)
Polyhydramnios	1 663	55.8 (53.1–58.6)	1 612	77.4 (73.6–81.3)	922	66.8 (62.5–71.4)
Intrauterine growth restriction/poor fetal growth	4 412	148.0 (143.6–152.5)	4 240	203.5 (197.4–209.8)	2 370	171.8 (164.9–179.0)
Gestational diabetes mellitus	5 352	179.5 (174.5–184.6)	5 199	249.6 (242.6–256.6)	2 941	213.2 (205.4–221.3)
**Assessed during period as stated in Table **1						
Maternal death	20	0.7 (0.4–1.1)	14	0.7 (0.3–1.2)	11	0.8 (0.4–1.5)
Preterm premature rupture of membranes	3 362	112.8 (109.0–116.7)	2 918	140.1 (135.0–145.2)	1 730	125.4 (119.6–131.5)
Fetal/perinatal distress or asphyxia	39 319	1 318.7 (1 306.2–1 331.3)	38 006	1 824.3 (1 807.4–1 841.3)	25 283	1 833.0 (1 812.3–1 853.9)

*AP* All Pregnancies, *AP24*+ All Pregnancies with gestational age ≥ 24 weeks, *CI* confidence interval, *Incidence* incidence proportion per 10 000 pregnancies, *LR* Low-Risk pregnancies, *n* number of pregnancies belonging to the specified category, *N* number of pregnancies included in the analysis in each cohort. If a particular pregnancy-related event of interest occurred several times for the same pregnancy, it was only counted once for that pregnancy

The incidence proportions of pregnancy-related EIs were lower in the LR cohort than the AP24+ cohort for 10 out of the 15 EIs examined: vaginal or intrauterine hemorrhage, pre-eclampsia, pregnancy-related hypertension, liver or biliary disease, premature/preterm labor, oligohydramnios, polyhydramnios, intrauterine growth restriction/poor fetal growth, gestational diabetes, and preterm premature rupture of membranes (based on non-overlapping CIs, Table 4). The incidence proportions of maternal sepsis, eclampsia, labor protraction/arrest disorders, maternal death, and fetal/perinatal distress or asphyxia were similar in the AP24+ and LR cohorts (overlapping CIs, Table 4).

### Exploratory stratification of study endpoints by select variables

**Fig. 5:**
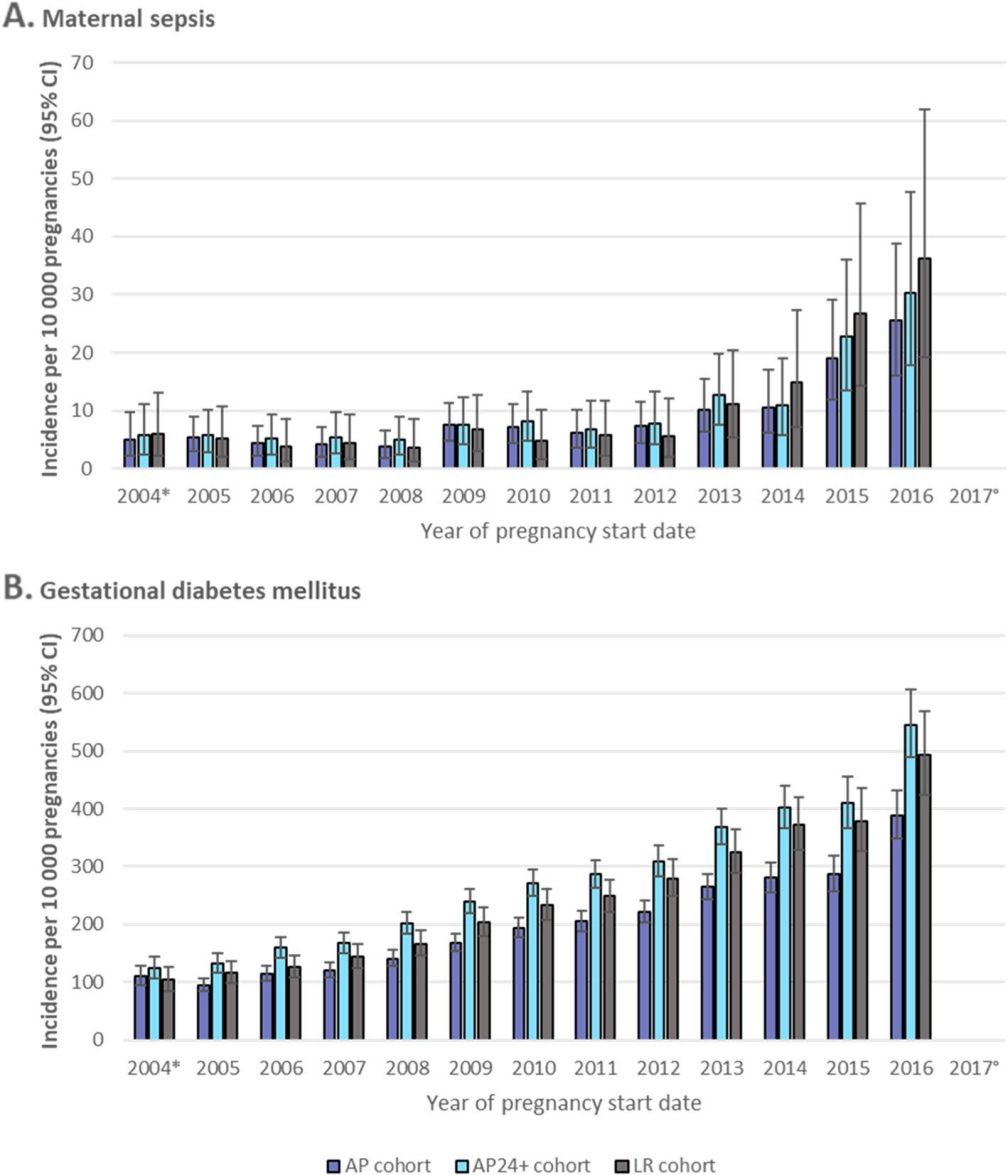
Incidence proportions of maternal sepsis **A** and gestational diabetes **B** by year of pregnancy start date. *AP*, All Pregnancies; *AP24+* , All Pregnancies with gestational age ≥ 24 weeks; *CI*, Confidence interval; *LR*, Low-Risk pregnancies. *Because the study start date was 1 January 2005, pregnancies reported as starting in 2004 include only those which began in the last 9 months of 2004 (if full term, for example)*. *°Pregnancies with a start date of 2017 were not included in this figure because the number was extremely low (and therefore incidence proportions less robust). Pregnancies reported as starting in 2017 included only those which began in the first month of 2017 (if full term, for example) because the study period end date was 31 December 2017 (with pregnancy end date up until 2 October 2017, as there was a requirement for at least a 90-day follow up after the pregnancy end date)

The incidence proportions of pregnancy outcomes and most pregnancy-related EIs remained relatively constant by calendar year of pregnancy start date across all cohorts (Additional file 9, Tables S9.1–S9.25). However, an increase was observed for some, including maternal sepsis, gestational diabetes, and intrauterine growth restriction/poor fetal growth (Additional file 9, Tables S9.8, S9.19, S9.18). The incidence proportion of gestational diabetes increased approximately four-fold in each cohort between 2005 and 2016, while that of maternal sepsis remained constant until 2012 and then increased between three- and seven-fold in the three cohorts between 2012 and 2016 (Fig. 5 and Additional file 9, Tables S9.19 and S9.8). The incidence proportion of intrauterine growth restriction/poor fetal growth increased about two-fold in each cohort between 2005 and 2016 (Additional file 9, Table S9.18).

Across all cohorts, the incidence proportions of pregnancy outcomes and pregnancy-related EIs generally varied by maternal age, ethnicity, and IMD quintile; however, observed patterns of risk were complex and non-uniform. For example, the incidence proportions of several pregnancy outcomes (e.g., stillbirth, preterm delivery) and pregnancy-related EIs (e.g., gestational diabetes, polyhydramnios) were highest amongst pregnancies with advanced maternal age, non-white race and higher socioeconomic deprivation levels (Additional file 9, Tables S9.2, S9.7, S9.19, and S9.17). By contrast, the incidence proportions of other pregnancy-related EIs (e.g., vaginal or intrauterine hemorrhage, labor protraction/arrest disorders, and intrauterine growth restriction/poor fetal growth) were lowest among pregnancies with advanced maternal age (Additional file 9, Tables S9.9, S9.15 and S9.18). Additionally, the incidence proportion of pregnancy-related hypertension was lowest in pregnancies where the women were the most deprived (Additional file 9, Table S9.12).

## Discussion

This descriptive, retrospective cohort study based on CPRD and linked data showed that the incidence proportions of pregnancy outcomes and pregnancy-related EIs represented in the CPRD varied between a cohort including all pregnancies, a cohort including all pregnancies with a GA ≥ 24 weeks, and a cohort including only low-risk pregnancies with a GA ≥ 24 weeks. This demonstrates the importance of accounting for GA and maternal risk profile when establishing background rates for a population of interest.

Because (by definition) the AP24+ and LR cohorts only included pregnancies with a GA of at least 24 weeks, the median duration of pregnancy was 7 days shorter with a much wider IQR in the AP cohort than the AP24+ and LR cohorts. The impact of the GA restriction was reflected in the observed rates of pregnancy outcomes. For instance, the incidence proportions of pregnancies resulting in live birth and preterm delivery (outcomes normally occurring after 24 weeks GA) were notably lower in the AP cohort than the AP24+ or LR cohorts. By contrast, the incidence proportion of termination was higher in the AP cohort than the AP24+ and LR cohorts as this outcome is expected to occur early in pregnancy (i.e., prior to 24 weeks GA). For the same reason, ectopic pregnancies could not be assessed in the AP24+ and LR cohorts. Neither could miscarriages and miscarriages or terminations (composite endpoint) because miscarriages with a GA > 24 weeks were reclassified as stillbirths in our study. When focusing on the AP24+ and LR cohorts, the incidence proportions of live birth, stillbirth, and termination were similar between cohorts. However, the incidence proportion of preterm delivery was lower in the LR cohort than the AP24+ cohort, potentially as a result of the exclusion of pregnant women with known risk factors for preterm delivery (e.g., hypertension [18]).

Due to the inclusion criterion of ≥ 24 weeks GA in the AP24+ and LR cohorts, which corresponds to a likely timing of enrollment in a maternal immunization trial [26–28], the AP24+ and LR cohorts are the most relevant for understanding the background rates of pregnancy-related EIs that might be expected in maternal immunization trials. For 10 of the EIs examined, lower incidence proportions were reported in the LR cohort relative to the AP24+ cohort (based on non-overlapping CIs). For the 5 remaining EIs examined, the incidence proportions in the LR cohort were similar to those in the AP24+ cohort. These results suggest that maternal risk profile (as defined by the presence of certain medical conditions and/or procedures in a woman’s available medical history and up to 24^0/7^ weeks GA in the current study) influences the likelihood of developing certain pregnancy-related EIs more strongly than others. For some EIs, the lower incidence proportions may be explained by these being included as exclusion criteria for the LR cohort (e.g., gestational hypertension).

Although the requirement for pregnancies in all cohorts to have a linkage to HES restricted the study population to England only, the rates of pregnancy outcomes reported in this study are largely consistent with available reports from independent sources for England and Wales, supporting the external validity and generalizability of the results for these areas. Extrapolation to other high-income areas should be done with caution as population dynamics may vary. For example, the ONS reported annual rates of stillbirth in England and Wales decreasing from 54 per 10 000 births in 2005 to 42 per 10 000 births in 2017 [38]. In the current study, 42.7 stillbirths per 10 000 pregnancies were reported in the AP cohort in 2005 and 39.6/10 000 in 2016. The UK National Health Service estimated that 1 in 8 pregnancies (12.5%) ends in miscarriage [39]; in the current study, 1 379.5 miscarriages per 10 000 pregnancies (13.8%) were reported for the AP cohort over the entire study period. Similarly, the prematurity rate in England and Wales was 7.3 per 100 live births in 2012 [40]; for the same year, 609.5 premature deliveries per 10 000 pregnancies were reported in the AP cohort in the current study. The ONS reported that 22.7% of conceptions among women resident in England and Wales in 2017 led to a legal abortion [41]. This is four times higher than the termination rate observed in the AP cohort of the current study (522.9/10 000 pregnancies; 5.2%). An underestimation of termination rates was also observed by Minassian et al. in their external validation of the Pregnancy Register [29].

There was a decrease in the number of pregnancies identified in CPRD by calendar year over the study period. This reflects a decrease in the number of English general practices contributing data to CPRD GOLD over time as well as a decline in the fertility rate in England and Wales in recent years (from 1.94 in 2012 to 1.76 in 2017) [38, 41, 42].

An increase in the incidence proportions of maternal sepsis and gestational diabetes over time was observed in the current study, which is consistent with reports for both maternal sepsis in the US [43] and gestational diabetes [44, 45]. For maternal sepsis, which increased sharply from 2012 onwards, this was likely driven by a combination of true changes in incidence, coding changes (Read code “A3C..00: Sepsis” was introduced in 2012), changes in screening and testing practices, and an increased clinical awareness of signs and symptoms [46]. For gestational diabetes, this increase may also have been driven by revised diagnostic criteria and an increased clinician awareness following the publication of the Hyperglycemia and Adverse Pregnancy Outcome (HAPO) study, which was conducted during the study period [47]. The current study also showed an increase in the incidence proportion of intrauterine growth restriction/poor fetal growth over time, which may in part be explained by changes in screening and diagnosis guidelines, e.g., an update on the management for the small for GA fetus in the Royal College of Obstetricians and Gynecologists guidelines and the publication of the Perinatal Institute’s “Growth Assessment Protocol” in 2013 [48–50]. The observed increase in these three endpoints highlights the importance of understanding changes in epidemiology and clinical practices over time when conducting retrospective studies with a long study period (e.g., 2005–2017 in the current study) or when selecting historical controls in real-world studies.

A key strength of this study is the use of the CPRD Pregnancy Register as the primary data source. As one of the largest and best-established primary care databases for research, the CPRD and the available linked datasets provide a rich and generalizable source of data on antenatal care, postnatal care, and pregnancy outcomes for England. Consistently, in the current study, the distribution of women across the five IMD quartiles was similar to the female population of England aged 18–45 years [51]. The recently validated CPRD Pregnancy Register leverages all available pregnancy data to identify pregnancy episodes. It has been demonstrated to closely agree with external hospitalization data in terms of the completeness and timing of pregnancy outcomes. However, some pregnancy outcomes such as termination and live birth appear to be underestimated in the Pregnancy Register as compared to data from the Department of Health and Social Care and ONS, respectively [29].

Another strength of this study is the application of the standardized case definitions established by the Brighton Collaboration and GAIA project for use in maternal immunization trials [35, 36]. These definitions were used to guide the selection and determination of study endpoints. Although the exact application of clinical case definitions was at times difficult within the context of this database study, with diagnoses recorded under the Read and ICD-10 systems, the incorporation of the GAIA guidance and philosophy contributes to the broad applicability and interest of the study results. This study may also help optimize the design of future studies (e.g., maternal immunization studies) by providing background rates of certain pregnancy outcomes and pregnancy-related EIs.

The major limitation of this study is its descriptive nature, which limits the strength of the conclusions that can be extracted from the analysis, particularly for the exploratory stratification of study endpoints by maternal age, ethnicity, and IMD. Demographic and temporal changes can substantially impact wider applicability of the present data to other populations. The vaccination history of the mother may also influence some outcomes. As this was not assessed, the potential impact could not be determined. In addition, the exclusion of a large proportion of pregnancies as a result of the required ≥ 365-day baseline period may have introduced selection bias. Cohort selection is also a limitation of this study. The LR cohort was selected to represent pregnant women likely to be enrolled in maternal immunization trials. However, coding limitations inherent to database studies (e.g., past medical conditions may be included as current diagnoses) may have led to erroneous exclusions from the LR cohort. On the other hand, past medical conditions or behavioral risk factors may have been omitted, thereby including high-risk pregnancies in the LR cohort. Another limitation is the possible presence of coding errors in the source data. Although the impact of coding errors is expected to be minimal based on prior CPRD validation studies for different disease states [52–54], they could have influenced incidence proportions. Additionally, Read and ICD-10 codes were used to identify the study outcomes and could have led to over- or underreporting of outcomes. Nevertheless, the study contributes to the evidence that maternal characteristics, including medical history and health-related risk factors, influence pregnancy outcomes and pregnancy-related EIs.

## Conclusions

Before conducting maternal immunization trials, it is essential to understand the background incidence proportions of pregnancy outcomes and pregnancy-related EIs in specific populations to evaluate and reliably interpret and monitor the safety of maternal vaccine candidates. This real-world analysis, using English primary and secondary care data that are largely representative of the general population, addressed this knowledge gap by generating the incidence proportions of a comprehensive list of pregnancy outcomes and pregnancy-related EIs in all and low-risk pregnancies represented in the CPRD Pregnancy Register. The results of this study demonstrate the importance of considering both the GA of a pregnancy episode and maternal risk factors when establishing background rates for a population of interest. These data may facilitate the interpretation of safety data from maternal immunization trials and the safety monitoring of maternal vaccines. In addition, these data can be of interest for any intervention studied in populations of pregnant women.

## Supplementary Information

**Additional file 1.** Exclusion criteria for the all pregnancies cohort. 

**Additional file 2.** Identification algorithm and assessment period for low-risk cohort exclusion criteria. 

**Additional file 3.** Codes used to identify exclusion criteria for the low-risk cohort. 

**Additional file 4.** Endpoints with GAIA definitions and the feasibility of applying these using CPRD data. 

**Additional file 5.** Codes used to identify pregnancy outcomes and pregnancy-related events of interest. 

**Additional file 6.** Maternal death. 

**Additional file 7.** Variable definitions and measures. 

**Additional file 8.** Codes used to define the variables. 

**Additional file 9.** Incidence proportions of pregnancy outcomes and pregnancy-related events of interest per 10 000 pregnancies by study cohort and select variables. 

## Data Availability

Study documents can be requested for further research from www.clinicalstudydatarequest.com. The CPRD and linked data used in this study cannot be shared directly with others due to contractual agreements. CPRD data may be requested from enquiries@cprd.com.

## Abbreviations

AP: All Pregnancies
AP24+: All Pregnancies with gestational age ≥ 24 weeks
CI: Confidence interval
CPRD: Clinical Practice Research Datalink
EI: Event of interest
GA: Gestational age
GAIA: Global Alignment of Immunization Safety Assessment
GBS: Group B streptococcus
HES: Hospital Episode Statistics
ICD-10: International Classification of Diseases, 10^th^ Revision
IQR: Interquartile range
ISAC: Independent Scientific Advisory Committee
IMD: Index of Multiple Deprivation
LR: Low-Risk
ONS: Office for National Statistics
RSV: Respiratory syncytial virus
UK: United Kingdom
US: United States
