# Supplementary material for: Facilitating safety evaluation in maternal immunization trials: a retrospective cohort study to assess pregnancy outcomes and events of interest in low-risk pregnancies in England
Source: BMC Pregnancy Childbirth. 2022 Jun 1;22:461. doi: 10.1186/s12884-022-04769-x (PMC9157029; doi:10.1186/s12884-022-04769-x)
Supplement: Supplementary file 1 — Additional file 1. Exclusion criteria for the all pregnancies cohort. [file 12884_2022_4769_MOESM1_ESM.docx]

**Additional file 1. Exclusion criteria for the All Pregnancies cohort**

- Pregnancy episodes associated with multiple births (e.g., twins, triplets)

Such episodes were identified in the Clinical Practice Research Datalink (CPRD) Pregnancy Register using the variable “multiple-ev” (flag to indicate evidence of a multiple pregnancy taking values 1=multiple, 0=no evidence of multiple). Pregnancy episodes where “multiple-ev”=1 or “mblbabies” >1 were excluded.

- Pregnancies with an unknown outcome (outcome=13)

Pregnancy episodes with an unknown outcome (variable “outcome=13”) generated by the CPRD algorithm are made up of antenatal records that do not fit inside any of the pregnancy episodes. For these episodes, the estimated episode end is the date of the last antenatal record in the episode and the estimated last menstrual period is 4 weeks before the first antenatal record. As recommended by CPRD, due to the lack of sufficient information for these pregnancies such as an accurate outcome, and pregnancy end and start dates, for the purpose of this study, pregnancies with outcome unknown were excluded from the analysis.

Pregnancies with a Pregnancy Register outcome=13 but ICD-10 codes or birstat variables in HES indicating specific outcomes (e.g., stillbirth, termination, live birth, miscarriage) were not excluded (see Additional file 4 for details on the algorithms used for these cases).
