# Supplementary material for: Facilitating safety evaluation in maternal immunization trials: a retrospective cohort study to assess pregnancy outcomes and events of interest in low-risk pregnancies in England
Source: BMC Pregnancy Childbirth. 2022 Jun 1;22:461. doi: 10.1186/s12884-022-04769-x (PMC9157029; doi:10.1186/s12884-022-04769-x)
Supplement: Supplementary file 2 — Additional file 2. Identification algorithm and assessment period for low-risk cohort exclusion criteria. [file 12884_2022_4769_MOESM2_ESM.docx]

**Additional file 2. Identification algorithm and assessment period for Low-Risk cohort exclusion criteria**

| Exclusion criterion | Identification algorithm | Assessment period |
| --- | --- | --- |
| Diagnoses identified during pregnancy | | |
| Incompetent cervix or cerclage | Read codes in CPRD or ICD-10 codes/OPCS procedure codes in HES* | From the start of pregnancy through 24^0/7^ weeks GA |
| Low-lying placenta or placenta previa with or without hemorrhage | Read codes in CPRD or ICD-10 codes in HES* |  |
| Polyhydramnios or oligohydramnios |  |  |
| Fetal anomalies |  |  |
| Gestational hypertension |  |  |
| Rubella infection |  |  |
| Gestational diabetes mellitus |  |  |
| Obstetric cholestasis |  |  |
| Abnormal finding in nuchal translucency scan, serum testing, or other prenatal tests |  |  |
| Intrauterine growth restriction |  |  |
| Cytomegalovirus infection |  |  |
| Herpes simplex infection |  |  |
| Toxoplasma infection |  |  |
| Parvovirus B19 infection |  |  |
| Syphilis infection |  |  |
| Diagnoses identified in the women’s medical history | | |
| A diagnosis of heart disease, cardiac disorders, or cardiac surgery | Read codes in CPRD or ICD-10 codes/OPCS procedure codes in HES* | Entire available medical record until 24^0/7^ weeks GA |
| Deep vein thrombosis, pulmonary embolism, or thrombophilia | Read codes in CPRD or ICD-10 codes in HES* |  |
| A diagnosis of asthma and/or chronic obstructive pulmonary disorder |  |  |
| A diagnosis of neuropsychiatric illness |  |  |
| Hypertension |  |  |
| Thyroid dysfunction |  |  |
| Hepatitis or liver disease |  |  |
| Immunosuppressive or immunodeficient condition |  |  |
| Chronic kidney disease |  |  |
| Neurological disease |  |  |
| Human immunodeficiency virus |  |  |
| Hepatitis B virus |  |  |
| Hepatitis C virus |  |  |
| Women with a prescription record of any of the following treatments: | | |
| With the following ongoing medical or surgical intervention to prevent preterm delivery:   - Cerclage - Progesterone - Tocolytics (e.g., terbutaline, nifedipine, magnesium sulfate, betamethasone) | Gemscript codes in CPRD or surgical intervention identified with OPCS codes in HES to prevent preterm delivery* | From the start of pregnancy through 24^0/7^ weeks GA |
| Involving administration of blood products | Read codes in CPRD or OPCS codes in HES* |  |
| Antihypertensive medication | Gemscript codes in CPRD* |  |

CPRD, Clinical Practice Research Datalink; GA, gestational age; HES, Hospital Episode Statistics; ICD-10, International Classification of Diseases, 10th Revision; OPCS, Office of Population Censuses and Surveys classification of procedures and interventions.

*See Additional file 3 for the codes.
