# Supplementary material for: Facilitating safety evaluation in maternal immunization trials: a retrospective cohort study to assess pregnancy outcomes and events of interest in low-risk pregnancies in England
Source: BMC Pregnancy Childbirth. 2022 Jun 1;22:461. doi: 10.1186/s12884-022-04769-x (PMC9157029; doi:10.1186/s12884-022-04769-x)
Supplement: Supplementary file 4 — Additional file 4. Endpoints with GAIA definitions and the feasibility of applying these using CPRD data. [file 12884_2022_4769_MOESM4_ESM.docx]

**Additional file 4. Endpoints with GAIA definitions and the feasibility of applying these using CPRD data**

| **Pregnancy outcomes and pregnancy-related events of interest** | **GAIA definition** | **Identification algorithm** | **Feasibility of identification in the CPRD Pregnancy Register and HES** |
| --- | --- | --- | --- |
| Fetal death/stillbirth [1] | Fetal death occurs prior to the evidence of labor (antepartum)  OR  Fetal death occurs during labor and before delivery (intrapartum) | Fetal death/stillbirth is recorded in the CPRD Pregnancy Register, or as a diagnosis ICD-10 code and a birstat variable in HES if the pregnancy outcome=13*^†^. If the pregnancy outcome=4^†^ and gestational age is ≥168 days, the outcome was changed to stillbirth | Pregnancy outcomes are well recorded in the CPRD Pregnancy Register |
| Elective/therapeutic termination | No GAIA definition | Termination of pregnancy in the CPRD Pregnancy Register, or ICD-10 code in HES if the pregnancy outcome=13*^†^ | Termination of pregnancy is recorded in the CPRD Pregnancy Register; however, we cannot determine whether this was elective |
| Preterm birth [2] | **Preterm birth** [3]:  A birth that occurs <37 weeks of gestation  **Pathway to preterm birth case definition** [4]:  a clinical syndrome characterized by any one  or some combination of the following four  pathways:  • Premature preterm rupture of  membranes  • Preterm labor  • Insufficient cervix  • Provider-initiated preterm birth | Preterm birth is recorded in the CPRD Pregnancy Register, or as a diagnosis ICD-10 code in HES*  OR  a live birth that occurred before 37 weeks of gestation using information from the CPRD Pregnancy Register on the estimated start and end date of pregnancy | Pregnancy outcomes are well recorded in the CPRD Pregnancy Register |
| Live birth | No GAIA definition | Live births were identified using the CPRD Pregnancy Register, and ICD-10 codes and birstat variables in HES if the pregnancy outcome=13*^†^. If the pregnancy outcome=2^†^ and there was a link with the MBL, the outcome was changed to live birth | Pregnancy outcomes are well recorded in the CPRD Pregnancy Register |
| Miscarriages [5] | GAIA defines spontaneous abortion as early pregnancy loss.  **First trimester spontaneous abortion:** Crown-rump length >7 mm and no visible heartbeat on transvaginal ultrasound  or  Crown-rump length >15 mm and no visible heartbeat on transvaginal ultrasound  or  Ultrasound examination demonstrating mean gestational sac diameter >25 mm and no visible embryo or yolk sac  AND  Second transvaginal ultrasound >7 days later (or 14 days later if transabdominal)  confirming diagnosis of non-viable pregnancy  or  Absence of embryo with heartbeat >2 weeks after a transabdominal scan that showed a gestational sac without a yolk sac  or  Absence of embryo with heartbeat >11 days after a transvaginal scan that showed a gestational sac with a yolk sac  **Alternatively**:  Gestational age within pre-defined range for selected abortion definition as assessed by maternal and/or fetal parameters (Level 1–2) (using the Brighton Preterm Birth Gestational Age algorithm).  AND  Positive urine or blood pregnancy test that becomes negative after 7 days  or  Products of conception found on histopathological evaluation of pregnancy tissue  or  Ultrasound examination demonstrating an empty uterine cavity in a woman who had clear evidence of intrauterine pregnancy on previous ultrasound examination  or  Vaginal bleeding, external cervical or open or closed with visible expulsion of pregnancy tissue/products of conception  **Second trimester spontaneous abortion:** Gestational age within pre-defined range for selected abortion definition as assessed by maternal and/or fetal-neonatal parameters (Level 1–2) (using Brighton Preterm Birth Gestational Age algorithm).  AND  No visible heartbeat on ultrasound  or  Visible expulsion of pregnancy tissues/products of conception on examination of the cervix  or  Products of conception found on histopathological evaluation of uterine  contents | Miscarriages were identified using the  CPRD Pregnancy Register, and ICD-10 codes if the pregnancy outcome=13*^†^  If the pregnancy outcome=4^†^ and gestational age is ≥168 days, the outcome was changed to stillbirth | Pregnancy outcomes are well recorded in the CPRD Pregnancy Register |
| Ectopic pregnancy [5] | Gestational age within pre-defined range for selected ectopic pregnancy definition as assessed by maternal and/or fetal-neonatal parameters (Level 1–2) (using Brighton Preterm Birth Gestational Age algorithm).  AND  B-HCG serum blood test >2000 mlU/ml  AND  TVUS showing no intrauterine pregnancy  OR  Gestational age within pre-defined range for selected ectopic pregnancy definition as assessed by maternal and/or fetal-neonatal parameters (Level 1–2) (using Brighton Preterm Birth Gestational Age algorithm).  AND  TVUS showing extrauterine pregnancy  OR  No products of conception found on endometrial curettage after D&C procedure | Ectopic pregnancies were identified  using the CPRD Pregnancy Register | Pregnancy outcomes are well recorded in the CPRD Pregnancy Register |
| Termination or miscarriage (composite endpoint) |  | Termination or miscarriage is recorded in the CPRD Pregnancy Register |  |
| Maternal death [6] | Death of the mother while pregnant or within 42 days of termination of pregnancy, irrespective of the duration and site of the pregnancy, from any cause related to aggravated by the pregnancy or its management but not from accidental or incidental causes | Death recorded as date of death in CPRD^#^ or in ONS* | Expected to be well recorded in the data |
| Maternal sepsis | No GAIA definition | Read codes in CPRD or ICD-10 codes in HES* | Diagnosis codes expected to be well recorded in the data |
| Vaginal or intrauterine hemorrhage [7] | **Postpartum hemorrhage:** Genital bleeding after delivery leading to severe maternal outcome (maternal death or near miss) as defined by WHO | Read codes in CPRD or ICD-10 code in HES indicative of hemorrhage in early pregnancy or antepartum hemorrhage* | Diagnosis codes expected to be well recorded in the data |
| Premature/preterm labor [4] | Patient is determined to have delivered preterm (at less than 37 weeks gestation complete).  On presentation, >4 documented uterine contractions per hour as determined by tocodynamometer  AND  Documented change in length of dilation of cervix by physical examination or transvaginal ultrasound over a 2-hour period, with clinical criteria for documenting cervical change by exam including:  Cervical dilation 2 cm or greater at the internal os by digital examination, cervical length of 1 cm or less by digital examination,  50% or greater effacement by digital examination | Read codes in CPRD or ICD-10 codes in HES indicative of premature/preterm labor* | It is likely that preterm labor is under-recorded in CPRD, and partially recorded in HES |
| Labor protraction/arrest disorders [8] | **First stage of labor:** Progress of less than 0.5 cm cervical dilation per hour, for at least 4 hours, in women in established labor (i.e., regular contractions and cervical dilation of at least 4 cm) and with confirmed ruptured membranes.  **Second stage of labor:** Full dilation of the cervix  AND  onset of the active stage (active maternal effort or visible baby)  AND  in nulliparous women >2 hours of pushing, in multiparous >1 hour of pushing  or  use of instrument for the indication of dystocia  or  cesarean delivery for the indication of dystocia.  Note: in the GAIA definitions this is the definition for dysfunctional labor. | Read codes in CPRD or ICD-10 codes in HES indicative of labor protraction/arrest disorders* | Clinical observations are not detailed throughout the stages of labor, hence identification of case was determined by diagnosis codes. It is likely that long or abnormal labor is very poorly recorded in CPRD, and partially recorded in HES |
| Pre-eclampsia and eclampsia [9] | **Pre-eclampsia:** Pregnancy ≥20 weeks and up to 6 weeks post-partum.  AND  New-onset hypertension (systolic blood pressure ≥140 mmHg and/or diastolic blood pressure ≥90 mmHg) sustained on two measurements over a minimum of 1 hour  AND  New-onset proteinuria  **Eclampsia:**  Severe features of pre-eclampsia | Read codes in CPRD and ICD-10 codes in HES* | Not expected to see test results recorded in the data, so the identification of cases was determined by diagnosis codes. It is expected that pre-eclampsia and eclampsia might be poorly recorded in HES |
| Pregnancy-related hypertension [9] | Blood pressure ≥140 mmHg (systolic)/≥90 mmHg (diastolic) that starts after 20 weeks of gestation (measured twice at least 1 hour apart)  AND  absence of protein or other stigmata of pre-eclampsia | Read codes in CPRD or ICD-10 codes in HES* | Diagnosis codes expected to be well recorded in the data |
| Preterm premature (pre-labor) rupture of membranes [4] | Clinical history of rupture of membranes  AND  Visible leakage of fluid on vaginal speculum exam  AND  Visible arborization (ferning) on microscopy of amniotic fluid  OR  Ultrasound with oligohydramnios (AFI <5 or MVP <2)  AND  Documented membrane rupture by a diagnostic test | Read codes in CPRD and ICD-10 codes in HES identified before 37 weeks of gestation* | Case definitions in combination with gestational age |
| Oligohydramnios [10] and polyhydramnios [11] | **Oligohydramnios:**  No GAIA definition.  **Standardized definition:** AFI <8 or DVP <2 **Polyhydramnios:**  No GAIA definition. **Standardized definition:** the presence of excess amniotic fluid in the uterus. By definition, polyhydramnios is diagnosed if the deepest vertical pool is more than 8 cm or AFI is more than 95^th^ percentile for the corresponding gestational age | Read codes in CPRD and ICD-10 codes in HES* | Laboratory test results not available in the data, however, diagnoses codes expected to be well reported in the data |
| Intrauterine growth restriction/poor fetal growth [12] | Fetal growth restriction is a sonographic finding characterized by certain parameters of estimated fetal weight.  Estimated fetal weight below 3% using locally accepted growth curve;  OR  Estimated fetal weight below 10% using locally accepted growth curve;  AND  Absent or reversed end-diastolic flow of the umbilical artery Doppler; OR  Oligohydramnios as defined as AFI <8 cm or DVP <2 cm in the presence of intact membranes without concern for fetal anomalies to its etiology | Read codes in CPRD and ICD-10 codes in HES* | Not expected to see test results recorded in the data, so the identification of cases determined by diagnosis codes. Typically, fetal outcomes are recorded in the free-text field of women’s records, which is not accessible to researchers. Therefore, these outcomes are expected to be underreported. Also difficult to differentiate between codes for intrauterine growth restrictions and small for gestational age |
| Gestational diabetes mellitus [13] | The absence of pre-gestational diabetes diagnosis defined by: Previous diagnosis of diabetes while not pregnant  or  First trimester hemoglobin A1c level of ≥6.5% (47.5 mmol/mol)  or  First trimester fasting blood glucose 126 mg/dL / ≥7 mmol/L  AND  Identification of sustained hyperglycemia during pregnancy | Read codes in CPRD and ICD-10 codes in HES identified in women with no prior diagnosis of diabetes (i.e., before the start date of pregnancy)* | Not expected to see test results recorded in the data, so identification of cases determined by diagnosis codes. Diagnoses codes expected to be well reported in the data. |
| Liver or biliary disease | No GAIA definition | Read codes in CPRD and ICD-10 codes in HES* | Not expected to see test results recorded in the data, so identification of cases determined by diagnosis codes. Diagnoses codes expected to be well reported in the data. |
| Fetal/perinatal distress [14] | Either of the following:  Recurrent late decelerations. Recurrent variable decelerations Bradycardia (<100 bpm)  OR  Sinusoidal pattern  AND  Umbilical cord blood analyses consistent with metabolic acidosis (pH <7.0 and Base deficit >12 mmol/L) | Read codes in CPRD and ICD-10 codes in HES* | Not expected to see test results recorded in the data, so identification of cases determined by diagnosis codes. Typically, fetal outcomes are recorded in the free-text field of women’s records, which is not accessible to researchers. Therefore, these outcomes are expected to be underreported. |

AFI, amniotic fluid index; B-HCG, beta human chorionic gonadotropin ; BPM, beats per minute; CPRD, Clinical Practice Research Datalink; D&C, dilation and curettage; DVP, deepest vertical pocket; GAIA, Global Alignment of Immunization Safety Assessment in Pregnancy; HES, Hospital Episode Statistics; ICD-10, International Classification of Diseases, 10^th^ Revision; MBL, mother-baby link; MVP, maximum vertical pocket; ONS, Office for National Statistics; TVUS, transvaginal ultrasounds; WHO, World Health Organization

*See Additional file 5 for the codes

^#^See Additional file 6

^†^Note, the CPRD Pregnancy Register uses the following pregnancy outcomes: 1 = Live birth, 2 = Stillbirth, 3 = 1 and 2, 4 = Miscarriage, 5 = Termination of pregnancy, 6 = Probable termination of pregnancy, 7 = Ectopic, 8 = Molar, 9 = Blighted ovum, 10 = Unspecified loss, 11 = Delivery based on a third trimester pregnancy record, 12 = Delivery based on a late pregnancy record, 13 = Outcome unknown [15].

**References**

1. Tavares Da Silva F, Gonik B, McMillan M, Keech C, Dellicour S, Bhange S et al. Stillbirth: Case definition and guidelines for data collection, analysis, and presentation of maternal immunization safety data. Vaccine. 2016;34(49):6057-68.

2. Howson CP, Kinney MV, McDougall L, Lawn JE. Born too soon: preterm birth matters. Reprod Health. 2013;10 Suppl 1(Suppl 1):S1.

3. Quinn JA, Munoz FM, Gonik B, Frau L, Cutland C, Mallett-Moore T et al. Preterm birth: Case definition & guidelines for data collection, analysis, and presentation of immunisation safety data. Vaccine. 2016;34(49):6047-56.

4. Harrison MS, Eckert LO, Cutland C, Gravett M, Harper DM, McClure EM et al. Pathways to preterm birth: Case definition and guidelines for data collection, analysis, and presentation of immunization safety data. Vaccine. 2016;34(49):6093-101.

5. Rouse CE, Eckert LO, Babarinsa I, Fay E, Gupta M, Harrison MS et al. Spontaneous abortion and ectopic pregnancy: Case definition & guidelines for data collection, analysis, and presentation of maternal immunization safety data. Vaccine. 2017;35(48 Pt A):6563-74.

6. Patwardhan M, Eckert LO, Spiegel H, Pourmalek F, Cutland C, Kochhar S et al. Maternal death: Case definition and guidelines for data collection, analysis, and presentation of immunization safety data. Vaccine. 2016;34(49):6077-83.

7. Kerr R, Eckert LO, Winikoff B, Durocher J, Meher S, Fawcus S et al. Postpartum haemorrhage: Case definition and guidelines for data collection, analysis, and presentation of immunization safety data. Vaccine. 2016;34(49):6102-9.

8. Boatin AA, Eckert LO, Boulvain M, Grotegut C, Fisher BM, King J et al. Dysfunctional labor: Case definition & guidelines for data collection, analysis, and presentation of immunization safety data. Vaccine. 2017;35(48 Pt A):6538-45.

9. Rouse CE, Eckert LO, Wylie BJ, Lyell DJ, Jeyabalan A, Kochhar S et al. Hypertensive disorders of pregnancy: Case definitions & guidelines for data collection, analysis, and presentation of immunization safety data. Vaccine. 2016;34(49):6069-76.

10. Preboth M. ACOG guidelines on antepartum fetal surveillance. American College of Obstetricians and Gynecologists. Am Fam Physician. 2000;62(5):1184, 7-8.

11. Hamza A, Herr D, Solomayer EF, Meyberg-Solomayer G. Polyhydramnios: Causes, Diagnosis and Therapy. Geburtshilfe Frauenheilkd. 2013;73(12):1241-6.

12. Easter SR, Eckert LO, Boghossian N, Spencer R, Oteng-Ntim E, Ioannou C et al. Fetal growth restriction: Case definition & guidelines for data collection, analysis, and presentation of immunization safety data. Vaccine. 2017;35(48 Pt A):6546-54.

13. Kachikis A, Eckert LO, Walker C, Oteng-Ntim E, Guggilla R, Gupta M et al. Gestational diabetes mellitus: Case definition & guidelines for data collection, analysis, and presentation of immunization safety data. Vaccine. 2017;35(48 Pt A):6555-62.

14. Gravett C, Eckert LO, Gravett MG, Dudley DJ, Stringer EM, Mujobu TB et al. Non-reassuring fetal status: Case definition & guidelines for data collection, analysis, and presentation of immunization safety data. Vaccine. 2016;34(49):6084-92.

15. Minassian C, Williams R, Meeraus WH, Smeeth L, Campbell OMR, Thomas SL. Methods to generate and validate a Pregnancy Register in the UK Clinical Practice Research Datalink primary care database. Pharmacoepidemiol Drug Saf. 2019;28(7):923-33.
