# Supplementary material for: Facilitating safety evaluation in maternal immunization trials: a retrospective cohort study to assess pregnancy outcomes and events of interest in low-risk pregnancies in England
Source: BMC Pregnancy Childbirth. 2022 Jun 1;22:461. doi: 10.1186/s12884-022-04769-x (PMC9157029; doi:10.1186/s12884-022-04769-x)
Supplement: Supplementary file 6 — Additional file 6. Maternal death. [file 12884_2022_4769_MOESM6_ESM.docx]

**Additional file 6. Maternal death**

Maternal death was identified based on the date of death in the Clinical Practice Research Datalink (CPRD) or the date of death in the Office for National Statistics (ONS). In the instance of conflicting information in the two sources, the date of death in ONS was used.

Maternal death was identified within three mutually exclusive time periods:

1. Death during pregnancy: death that occurred between the start of pregnancy until the day before (inclusive) the end of pregnancy date.

2. Death 24 hours after delivery: death that occurred on the day of delivery or 1 day after delivery.

3. Death after 42 days: Death that occurred between 2 and 42 days (inclusive) after delivery.

The frequency of maternal deaths in each of these time periods were reported.

*Deriving death in the CPRD*

1. Transfer Out Date (TOD) in patient file

TOD=Transfer Out Date if transfer out reason is Death (toreason=1) otherwise TOD=0

1. ‘Statement of Death’ (SOD) event dates (SODmin, SODmax) in clinical file

Search through patient’s clinical files for records of SOD Read codes - each event will have an associated event date. Ignore event dates that are empty, invalid, prior to first registration date of the patient, after the last collection date of the practice and those preceding patient’s TOD (when reason is death) by more than 95 days.

If SOD is a suicide code:

May refer to a completed patient suicide or an attempted suicide. Ignore event dates if patient has not transferred out, or reason for the transfer is not ‘Death’.

If SOD is a ‘Death of Baby’ code:

May be a record in the parent’s, sibling’s or the baby’s record. Ignore event dates if patient’s age at event is >2 years.

If SOD is a ‘Death of Mother’ code:

May be a record in the mother’s or baby’s record. Ignore event dates if patient’s age at event is <12 years.

A patient can have multiple records of a SOD Read code recorded in their Clinical file. For each patient, retain the earliest (SODmin), and latest (SODmax) event dates that meet above criteria. Patients with no records will have the SODmin and SODmax values set to 0.

1. Death administration entity type (ACD, date of death [DOD]) in clinical and additional files

Search through the patient’s clinical files for entity 148 (death administration), and a non-zero adid, that links with the patient’s additional file.

The clinical record provides the event date (ACD), and the data1 field of the record with entity 148 in the additional file provides a DOD.

Ignore event dates (ACD) and DOD that are empty, invalid, prior to first registration date of the patient, or after the last collection date of the practice.

For each death administration record, set DOD as the earliest of DOD and ACD dates.

For each patient, retain the earliest DOD date. If no such date, DOD=0.

1. Final derived death date (DEATHDATE)

For each patient, ignore the SOD event dates (SODmin and SODmax=0) if the patient has not transferred out (TOD=0), and does not have a record in the death administration structured data area (DOD=0).

Patient’s death date (DEATHDATE) is set to the earliest of (TOD, SODmin, DOD). If the patient does not have a record in the death administration structured data area (DOD=0), but has transferred out due to death, and both the earliest and latest SOD event dates (SODmin and SODmax) are within 95 days of the TOD, the patient’s death date (DEATHDATE) should be set to the latest SOD event dates (SODmax).
