# Supplementary material for: Facilitating safety evaluation in maternal immunization trials: a retrospective cohort study to assess pregnancy outcomes and events of interest in low-risk pregnancies in England
Source: BMC Pregnancy Childbirth. 2022 Jun 1;22:461. doi: 10.1186/s12884-022-04769-x (PMC9157029; doi:10.1186/s12884-022-04769-x)
Supplement: Supplementary file 7 — Additional file 7. Variable definitions and measures. [file 12884_2022_4769_MOESM7_ESM.docx]

**Additional file 7. Variable definitions and measures**

**Maternal age at start of pregnancy**

Women’s age was calculated at the estimated start of pregnancy (dd/mm/yyyy). Age was divided into five separate categories from 18–45 years: 18–24, 25–29, 30–34, 35–39, 40–45 years. Age was reported as a continuous as well as a categorical variable.

**Calendar year of start of pregnancy**

A calendar year (yyyy) was assigned to each pregnancy, based on the estimated start date of pregnancy (dd/mm/yyyy) from 2005 to 2017.

**Number of pregnancies in the study period**

The number of pregnancies per woman (0–9) recorded in the Pregnancy Register was described using continuous and categorical descriptive statistics.

**Ethnicity**

Women’s ethnicity was extracted from the Hospital Episode Statistics/Clinical Practice Research Datalink (CPRD). The six categories for ethnicity were white, Asian, Black, mixed, other, and unknown.

**Quintile of deprivation**

Women’s socioeconomic status was extracted from the linked Index of Multiple Deprivation data. There are five categories, with scores ranging from 1, indicating the least deprived to 5, indicating the most deprived.

**Pregnancy Number**

Pregnancy number was identified as categories (0–9) of the number of previous pregnancies recorded ever in the women’s records in the Pregnancy Register that resulted in a live birth.

**Contraception use**

Contraception use was extracted in the year prior to the start of pregnancy using Gemscript or Read codes in CPRD (See Additional file 8). It was categorized into oral, vaginal ring and intra-uterine device, implant, injection, patch diaphragm and other, and unknown. Women can have more than one type of contraception recorded. Condom use was not reported.

**Smoking status**

Smoking status was categorized into either current, former, non-smoker, or missing. The value closest to before the index date (i.e., estimated start of pregnancy) and within the 365 days pre-index was used. A single specific variable describing smoking status does not exist in the CPRD. Therefore, smoking status was derived using CPRD enttype code 4 and relevant medical terms (See Additional file 8).

Note that smoking status may be potentially under-recorded in the CPRD.

**Alcohol intake**

Alcohol intake was categorized into either light, moderate, heavy, non-drinker, or missing. The value closest to before the index date and within the 356 days pre-index was used. A single specific variable describing alcohol intake does not exist in the CPRD. Therefore, alcohol intake was derived using records with enttype 5 and Read codes in CPRD (See Additional file 8).
