# Supplementary material for: Facilitating safety evaluation in maternal immunization trials: a retrospective cohort study to assess pregnancy outcomes and events of interest in low-risk pregnancies in England
Source: BMC Pregnancy Childbirth. 2022 Jun 1;22:461. doi: 10.1186/s12884-022-04769-x (PMC9157029; doi:10.1186/s12884-022-04769-x)
Supplement: Supplementary file 9 — Additional file 9. Incidence proportions of pregnancy outcomes and pregnancy-related events of interest per 10 000 pregnancies by study cohort and select variables. [file 12884_2022_4769_MOESM9_ESM.docx]

**Additional file 9. Incidence proportions of pregnancy outcomes and pregnancy-related events of interest per 10,000 pregnancies by study cohort and select variables**

Note applicable to all tables:

*Because the study start date was 1 January 2005, the number of pregnancies reported as starting in 2004 includes only those which began in the last 9 months of 2004 (if full term, for example).

°Because the study period end date was 31 December 2017 (with pregnancy end date up until 2 October 2017, as there was a requirement for at least a 90-day follow-up after the pregnancy end date), the number of pregnancies reported as starting in 2017 includes only those which began in the first month of 2017 (if full term, for example).

**Table S9.1. Live birth**

|  |  | **AP cohort** | | | | |  | | **AP24+ cohort** | | | | | |  | **LR cohort** | | | | | |
| --- | --- | --- | --- | --- | --- | --- | --- | --- | --- | --- | --- | --- | --- | --- | --- | --- | --- | --- | --- | --- | --- |
|  |  | **n** | **Population under study** | **Incidence/**  **10 000** | **95% CI** | | |  | | **n** | **Population under study** | **Incidence/**  **10 000** | **95% CI** | |  | **n** | **Population under study** | **Incidence/**  **10 000** | **95% CI** | | |
| **Year of pregnancy start date** | | | | | | | | | | | | | | | | | | | | |  |
| **2004*** |  | 14209 | 16027 | 8865.7 | 8815.6 | 8914.4 | |  | | 13964 | 14041 | 9945.2 | 9931.5 | 9956.7 |  | 9985 | 10040 | 9945.2 | 9928.8 | 9958.7 | |
| **2005** |  | 19765 | 27609 | 7158.9 | 7106 | 7211.4 | |  | | 19111 | 19220 | 9943.3 | 9931.7 | 9953.4 |  | 13392 | 13456 | 9952.4 | 9939.4 | 9963.3 | |
| **2006** |  | 20194 | 28221 | 7155.7 | 7103.3 | 7207.6 | |  | | 19589 | 19706 | 9940.6 | 9929.0 | 9950.8 |  | 13454 | 13527 | 9946.0 | 9932.3 | 9957.6 | |
| **2007** |  | 20856 | 29076 | 7172.9 | 7121.4 | 7224 | |  | | 20161 | 20268 | 9947.2 | 9936.3 | 9956.6 |  | 13760 | 13830 | 9949.4 | 9936.1 | 9960.5 | |
| **2008** |  | 21040 | 29761 | 7069.7 | 7018.2 | 7120.7 | |  | | 20276 | 20382 | 9948.0 | 9937.2 | 9957.4 |  | 13647 | 13709 | 9954.8 | 9942.1 | 9965.3 | |
| **2009** |  | 20747 | 29342 | 7070.8 | 7018.8 | 7122.3 | |  | | 20005 | 20125 | 9940.4 | 9928.8 | 9950.5 |  | 13341 | 13421 | 9940.4 | 9925.9 | 9952.7 | |
| **2010** |  | 20094 | 27983 | 7180.8 | 7128.3 | 7232.8 | |  | | 19379 | 19479 | 9948.7 | 9937.7 | 9958.2 |  | 12699 | 12763 | 9949.9 | 9936.0 | 9961.4 | |
| **2011** |  | 19611 | 27209 | 7207.5 | 7154.5 | 7260.1 | |  | | 18967 | 19073 | 9944.4 | 9932.9 | 9954.4 |  | 12288 | 12349 | 9950.6 | 9936.7 | 9962.1 | |
| **2012** |  | 17510 | 24661 | 7100.3 | 7043.9 | 7156.1 | |  | | 16785 | 16888 | 9939.0 | 9926.1 | 9950.2 |  | 10636 | 10688 | 9951.3 | 9936.3 | 9963.6 | |
| **2013** |  | 14832 | 20812 | 7126.7 | 7065.3 | 7187.4 | |  | | 14194 | 14269 | 9947.4 | 9934.2 | 9958.6 |  | 8942 | 8982 | 9955.5 | 9939.4 | 9968.2 | |
| **2014** |  | 11335 | 16166 | 7011.6 | 6941 | 7081.6 | |  | | 10892 | 10959 | 9938.9 | 9922.6 | 9952.5 |  | 6658 | 6697 | 9941.8 | 9920.5 | 9958.6 | |
| **2015** |  | 8143 | 11514 | 7072.3 | 6989 | 7154.5 | |  | | 7874 | 7903 | 9963.3 | 9947.3 | 9975.4 |  | 4841 | 4856 | 9969.1 | 9949.1 | 9982.7 | |
| **2016** |  | 6119 | 8580 | 7131.7 | 7035.7 | 7226.3 | |  | | 5922 | 5954 | 9946.3 | 9924.4 | 9963.1 |  | 3569 | 3589 | 9944.3 | 9914.1 | 9965.9 | |
| **2017°** |  | 137 | 1194 | 1147.4 | 971.4 | 1342.8 | |  | | 57 | 61 | 9344.3 | 8394.8 | 9821.0 |  | 22 | 25 | 8800.0 | 6826.2 | 9756.7 | |
| **Age at pregnancy start date** | | | | | | | | | | | | | | | | | | | | |  |
| **18–24** |  | 41526 | 64282 | 6460.0 | 6422.7 | 6497.1 | |  | | 39851 | 40102 | 9937.4 | 9929.5 | 9944.6 |  | 25621 | 25770 | 9942.2 | 9932.4 | 9950.9 | |
| **25–29** |  | 56083 | 73199 | 7661.7 | 7631.7 | 7691.5 | |  | | 54155 | 54429 | 9949.7 | 9943.8 | 9955.1 |  | 35577 | 35746 | 9952.7 | 9945.5 | 9959.2 | |
| **30–34** |  | 67377 | 85923 | 7841.6 | 7814.9 | 7868.1 | |  | | 65185 | 65489 | 9953.6 | 9948.5 | 9958.3 |  | 44522 | 44713 | 9957.3 | 9951.1 | 9962.8 | |
| **35–39** |  | 40442 | 56947 | 7101.7 | 7065.2 | 7137.9 | |  | | 39132 | 39366 | 9940.6 | 9933.1 | 9947.4 |  | 25935 | 26076 | 9945.9 | 9936.9 | 9954.0 | |
| **40–45** |  | 9164 | 17804 | 5147.2 | 5073.4 | 5220.9 | |  | | 8853 | 8942 | 9900.5 | 9878.5 | 9919.3 |  | 5579 | 5627 | 9914.7 | 9888.5 | 9936.1 | |
| **Ethnicity (from HES data)** | | | | | | | | | | | | | | | | | | | | |  |
| **White** |  | 183662 | 252166 | 7283.4 | 7266.1 | 7300.6 | |  | | 177564 | 178527 | 9946.1 | 9942.8 | 9949.2 |  | 115239 | 115810 | 9950.7 | 9946.7 | 9954.4 | |
| **Asian** |  | 12526 | 16757 | 7475.1 | 7409.4 | 7539.9 | |  | | 11972 | 12054 | 9932.0 | 9917.6 | 9944.4 |  | 8867 | 8918 | 9942.8 | 9926.7 | 9956.2 | |
| **Black** |  | 5909 | 8821 | 6698.8 | 6599.2 | 6797.3 | |  | | 5631 | 5672 | 9927.7 | 9902.6 | 9947.7 |  | 4044 | 4071 | 9933.7 | 9904.0 | 9956.1 | |
| **Mixed** |  | 2409 | 3528 | 6828.2 | 6671.1 | 6982.3 | |  | | 2306 | 2320 | 9939.7 | 9900.0 | 9966.5 |  | 1509 | 1518 | 9940.7 | 9886.8 | 9973.2 | |
| **Other** |  | 4574 | 6277 | 7286.9 | 7175.0 | 7396.7 | |  | | 4332 | 4360 | 9935.8 | 9908.3 | 9956.7 |  | 3322 | 3342 | 9940.2 | 9907.8 | 9963.4 | |
| **Unknown** |  | 5512 | 10606 | 5197.1 | 5098.2 | 5295.8 | |  | | 5371 | 5395 | 9955.5 | 9934.6 | 9971.1 |  | 4253 | 4273 | 9953.2 | 9928.8 | 9970.9 | |
| **Index of Multiple Deprivation** | | | | | | | | | | | | | | | | | | | | |  |
| **1 (least)** |  | 45754 | 61945 | 7386.2 | 7351.7 | 7420.6 | |  | | 44400 | 44577 | 9960.3 | 9954.6 | 9965.4 |  | 30620 | 30724 | 9966.2 | 9959.5 | 9972.0 | |
| **2** |  | 42526 | 58360 | 7286.8 | 7250.7 | 7322.7 | |  | | 41051 | 41240 | 9954.2 | 9947.7 | 9960.0 |  | 27654 | 27772 | 9957.5 | 9949.7 | 9964.4 | |
| **3** |  | 42063 | 58758 | 7158.7 | 7122.1 | 7195.1 | |  | | 40632 | 40881 | 9939.1 | 9931.6 | 9946.0 |  | 27156 | 27328 | 9937.1 | 9927.5 | 9945.7 | |
| **4** |  | 42133 | 59246 | 7111.5 | 7074.8 | 7148.1 | |  | | 40650 | 40908 | 9936.9 | 9929.5 | 9943.7 |  | 26453 | 26604 | 9943.2 | 9934.2 | 9951.3 | |
| **5 (most)** |  | 41961 | 59612 | 7039.0 | 7002.2 | 7075.7 | |  | | 40294 | 40573 | 9931.2 | 9923.2 | 9938.6 |  | 25262 | 25415 | 9939.8 | 9929.9 | 9948.7 | |
| **Missing** |  | 155 | 234 | 6623.9 | 6012.1 | 7198.1 | |  | | 149 | 149 | 10000 |  |  |  | 89 | 89 | 10000 |  |  | |

AP, All Pregnancies; AP24+, All Pregnancies with gestational age ≥24 weeks; LR, Low-Risk pregnancies; n, number of pregnancies in the specified category; CI, confidence interval; HES, Hospital Episode Statistics.

**Table S9.2. Stillbirth**

|  |  | **AP cohort** | | | | |  | | **AP24+ cohort** | | | | | |  | **LR cohort** | | | | | |
| --- | --- | --- | --- | --- | --- | --- | --- | --- | --- | --- | --- | --- | --- | --- | --- | --- | --- | --- | --- | --- | --- |
|  |  | **n** | **Population under study** | **Incidence/**  **10 000** | **95% CI** | | |  | | **n** | **Population under study** | **Incidence/**  **10 000** | **95% CI** | |  | **n** | **Population under study** | **Incidence/**  **10 000** | **95% CI** | | |
| **Year of pregnancy start date** | | | | | | | | | | | | | | | | | | | | |  |
| **2004*** |  | 74 | 16027 | 46.2 | 36.3 | 57.9 | |  | | 69 | 14041 | 49.1 | 38.3 | 62.2 |  | 50 | 10040 | 49.8 | 37.0 | 65.6 | |
| **2005** |  | 118 | 27609 | 42.7 | 35.4 | 51.1 | |  | | 93 | 19220 | 48.4 | 39.1 | 59.2 |  | 53 | 13456 | 39.4 | 29.6 | 51.4 | |
| **2006** |  | 127 | 28221 | 45.0 | 37.6 | 53.4 | |  | | 111 | 19706 | 56.3 | 46.4 | 67.7 |  | 70 | 13527 | 51.7 | 40.4 | 65.2 | |
| **2007** |  | 117 | 29076 | 40.2 | 33.4 | 48.1 | |  | | 102 | 20268 | 50.3 | 41.1 | 60.9 |  | 67 | 13830 | 48.4 | 37.6 | 61.4 | |
| **2008** |  | 119 | 29761 | 40.0 | 33.1 | 47.8 | |  | | 97 | 20382 | 47.6 | 38.6 | 58.0 |  | 58 | 13709 | 42.3 | 32.1 | 54.7 | |
| **2009** |  | 124 | 29342 | 42.3 | 35.2 | 50.3 | |  | | 105 | 20125 | 52.2 | 42.7 | 63.1 |  | 73 | 13421 | 54.4 | 42.7 | 68.3 | |
| **2010** |  | 108 | 27983 | 38.6 | 31.6 | 46.6 | |  | | 88 | 19479 | 45.2 | 36.3 | 55.6 |  | 58 | 12763 | 45.4 | 34.5 | 58.7 | |
| **2011** |  | 118 | 27209 | 43.4 | 36.0 | 51.8 | |  | | 99 | 19073 | 51.9 | 42.3 | 63.0 |  | 55 | 12349 | 44.5 | 33.7 | 57.8 | |
| **2012** |  | 113 | 24661 | 45.8 | 37.8 | 55.0 | |  | | 92 | 16888 | 54.5 | 44.0 | 66.7 |  | 47 | 10688 | 44.0 | 32.4 | 58.3 | |
| **2013** |  | 85 | 20812 | 40.8 | 32.7 | 50.4 | |  | | 67 | 14269 | 47.0 | 36.4 | 59.6 |  | 36 | 8982 | 40.1 | 28.1 | 55.4 | |
| **2014** |  | 69 | 16166 | 42.7 | 33.3 | 53.9 | |  | | 62 | 10959 | 56.6 | 43.5 | 72.3 |  | 37 | 6697 | 55.2 | 38.9 | 76.1 | |
| **2015** |  | 28 | 11514 | 24.3 | 16.3 | 35.0 | |  | | 24 | 7903 | 30.4 | 19.5 | 45.2 |  | 13 | 4856 | 26.8 | 14.3 | 45.7 | |
| **2016** |  | 34 | 8580 | 39.6 | 27.5 | 55.2 | |  | | 29 | 5954 | 48.7 | 32.8 | 69.7 |  | 17 | 3589 | 47.4 | 27.6 | 75.7 | |
| **2017°** |  | 5 | 1194 | 41.9 | 13.5 | 97.9 | |  | | *#* | *#* | *#* | *#* | *#* |  | *#* | *#* | *#* | *#* | *#* | |
| **Age at pregnancy start date** | | | | | | | | | | | | | | | | | | | | |  |
| **18–24** |  | 266 | 64282 | 41.4 | 36.7 | 46.4 | |  | | 226 | 40102 | 56.4 | 49.5 | 63.8 |  | 133 | 25770 | 51.6 | 43.4 | 60.9 | |
| **25–29** |  | 301 | 73199 | 41.1 | 36.9 | 45.7 | |  | | 246 | 54429 | 45.2 | 40.1 | 50.8 |  | 154 | 35746 | 43.1 | 36.9 | 50.0 | |
| **30–34** |  | 321 | 85923 | 37.4 | 33.7 | 41.3 | |  | | 278 | 65489 | 42.4 | 38.0 | 47.3 |  | 173 | 44713 | 38.7 | 33.4 | 44.5 | |
| **35–39** |  | 256 | 56947 | 45.0 | 40.1 | 50.3 | |  | | 209 | 39366 | 53.1 | 46.7 | 60.1 |  | 130 | 26076 | 49.9 | 42.2 | 58.5 | |
| **40–45** |  | 95 | 17804 | 53.4 | 43.9 | 64.3 | |  | | 83 | 8942 | 92.8 | 74.6 | 114.1 |  | 47 | 5627 | 83.5 | 62.4 | 109.4 | |
| **Ethnicity (from HES data)** | | | | | | | | | | | | | | | | | | | | |  |
| **White** |  | 1022 | 252166 | 40.5 | 38.3 | 42.8 | |  | | 863 | 178527 | 48.3 | 45.4 | 51.4 |  | 513 | 115810 | 44.3 | 40.8 | 48.0 | |
| **Asian** |  | 94 | 16757 | 56.1 | 46.5 | 67.0 | |  | | 76 | 12054 | 63.0 | 51.3 | 76.7 |  | 50 | 8918 | 56.1 | 42.8 | 72.1 | |
| **Black** |  | 51 | 8821 | 57.8 | 42.9 | 76.2 | |  | | 40 | 5672 | 70.5 | 50.8 | 95.3 |  | 26 | 4071 | 63.9 | 42.0 | 93.0 | |
| **Mixed** |  | 18 | 3528 | 51.0 | 30.3 | 80.4 | |  | | 14 | 2320 | 60.3 | 33.5 | 100.0 |  | 9 | 1518 | 59.3 | 26.8 | 113.2 | |
| **Other** |  | 31 | 6277 | 49.4 | 34.2 | 69.0 | |  | | 27 | 4360 | 61.9 | 41.5 | 88.8 |  | 20 | 3342 | 59.8 | 36.6 | 92.2 | |
| **Unknown** |  | 23 | 10606 | 21.7 | 14.4 | 31.4 | |  | | 22 | 5395 | 40.8 | 26.1 | 60.8 |  | 19 | 4273 | 44.5 | 27.4 | 68.2 | |
| **Index of Multiple Deprivation** | | | | | | | | | | | | | | | | | | | | |  |
| **1 (least)** |  | 188 | 61945 | 30.3 | 26.7 | 34.4 | |  | | 157 | 44577 | 35.2 | 30.4 | 40.5 |  | 93 | 30724 | 30.3 | 24.8 | 36.6 | |
| **2** |  | 210 | 58360 | 36.0 | 31.8 | 40.6 | |  | | 169 | 41240 | 41.0 | 35.5 | 47.1 |  | 105 | 27772 | 37.8 | 31.4 | 45.2 | |
| **3** |  | 258 | 58758 | 43.9 | 39.0 | 49.2 | |  | | 226 | 40881 | 55.3 | 48.8 | 62.4 |  | 158 | 27328 | 57.8 | 49.6 | 67.0 | |
| **4** |  | 282 | 59246 | 47.6 | 42.7 | 52.9 | |  | | 239 | 40908 | 58.4 | 51.9 | 65.6 |  | 141 | 26604 | 53.0 | 45.2 | 61.8 | |
| **5 (most)** |  | 301 | 59612 | 50.5 | 45.3 | 56.1 | |  | | 251 | 40573 | 61.9 | 54.9 | 69.4 |  | 140 | 25415 | 55.1 | 46.7 | 64.6 | |
| **Missing** |  | 0 | 234 | 0 |  |  | |  | | 0 | 149 | 0 |  |  |  | 0 | 89 | 0 |  |  | |

AP, All Pregnancies; AP24+, All Pregnancies with gestational age ≥24 weeks; LR, Low-Risk pregnancies; n, number of pregnancies in the specified category; CI, confidence interval; #, results where an individual cell count is <5 (to maintain confidentiality); HES, Hospital Episode Statistics.

**Table S9.3. Miscarriage**

|  |  | **AP cohort** | | | | |  | | **AP24+ cohort** | | | | | |  | **LR cohort** | | | | | |  |
| --- | --- | --- | --- | --- | --- | --- | --- | --- | --- | --- | --- | --- | --- | --- | --- | --- | --- | --- | --- | --- | --- | --- |
|  |  | **n** | **Population under study** | **Incidence/**  **10 000** | **95% CI** | | |  | | **n** | **Population under study** | **Incidence/**  **10 000** | **95% CI** | |  | **n** | **Population under study** | **Incidence/**  **10 000** | **95% CI** | | |  |
| **Year of pregnancy start date** | | | | | | | | | | | | | | | | | | | | |  |  |
| **2004*** |  | 871 | 16027 | 543.5 | 508.9 | 579.7 | |  | | 0 |  | 0 |  |  |  | 0 |  | 0 |  |  | |  |
| **2005** |  | 3740 | 27609 | 1354.6 | 1315.8 | 1394.2 | |  | | 0 |  | 0 |  |  |  | 0 |  | 0 |  |  | |  |
| **2006** |  | 3769 | 28221 | 1335.5 | 1297.3 | 1374.5 | |  | | 0 |  | 0 |  |  |  | 0 |  | 0 |  |  | |  |
| **2007** |  | 3812 | 29076 | 1311.0 | 1273.6 | 1349.2 | |  | | 0 |  | 0 |  |  |  | 0 |  | 0 |  |  | |  |
| **2008** |  | 4101 | 29761 | 1378.0 | 1340.2 | 1416.4 | |  | | 0 |  | 0 |  |  |  | 0 |  | 0 |  |  | |  |
| **2009** |  | 4142 | 29342 | 1411.6 | 1373.0 | 1450.9 | |  | | 0 |  | 0 |  |  |  | 0 |  | 0 |  |  | |  |
| **2010** |  | 3849 | 27983 | 1375.5 | 1336.5 | 1415.2 | |  | | 0 |  | 0 |  |  |  | 0 |  | 0 |  |  | |  |
| **2011** |  | 3713 | 27209 | 1364.6 | 1325.3 | 1404.7 | |  | | 0 |  | 0 |  |  |  | 0 |  | 0 |  |  | |  |
| **2012** |  | 3676 | 24661 | 1490.6 | 1447.6 | 1534.4 | |  | | 0 |  | 0 |  |  |  | 0 |  | 0 |  |  | |  |
| **2013** |  | 3137 | 20812 | 1507.3 | 1460.1 | 1555.4 | |  | | 0 |  | 0 |  |  |  | 0 |  | 0 |  |  | |  |
| **2014** |  | 2475 | 16166 | 1531.0 | 1476.9 | 1586.2 | |  | | 0 |  | 0 |  |  |  | 0 |  | 0 |  |  | |  |
| **2015** |  | 1788 | 11514 | 1552.9 | 1488.5 | 1618.9 | |  | | 0 |  | 0 |  |  |  | 0 |  | 0 |  |  | |  |
| **2016** |  | 1395 | 8580 | 1625.9 | 1550.1 | 1703.8 | |  | | 0 |  | 0 |  |  |  | 0 |  | 0 |  |  | |  |
| **2017°** |  | 661 | 1194 | 5536.0 | 5247.7 | 5821.6 | |  | | 0 |  | 0 |  |  |  | 0 |  | 0 |  |  | |  |
| **Age at pregnancy start date** | | | | | | | | | | | | | | | | | | | | |  |  |
| **18–24** |  | 6374 | 64282 | 991.6 | 969.4 | 1014.0 | |  | | 0 |  | 0 |  |  |  | 0 |  | 0 |  |  | |  |
| **25–29** |  | 7926 | 73199 | 1082.8 | 1061.8 | 1104.1 | |  | | 0 |  | 0 |  |  |  | 0 |  | 0 |  |  | |  |
| **30–34** |  | 10772 | 85923 | 1253.7 | 1233.2 | 1274.4 | |  | | 0 |  | 0 |  |  |  | 0 |  | 0 |  |  | |  |
| **35–39** |  | 10250 | 56947 | 1799.9 | 1770.3 | 1829.8 | |  | | 0 |  | 0 |  |  |  | 0 |  | 0 |  |  | |  |
| **40–45** |  | 5807 | 17804 | 3261.6 | 3193.7 | 3330.1 | |  | | 0 |  | 0 |  |  |  | 0 |  | 0 |  |  | |  |
| **Ethnicity (from HES data)** | | | | | | | | | | | | | | | | | | | | |  |  |
| **White** |  | 34585 | 252166 | 1371.5 | 1358.7 | 1384.4 | |  | | 0 |  | 0 |  |  |  | 0 |  | 0 |  |  | |  |
| **Asian** |  | 2336 | 16757 | 1394.0 | 1344.1 | 1445.1 | |  | | 0 |  | 0 |  |  |  | 0 |  | 0 |  |  | |  |
| **Black** |  | 1321 | 8821 | 1497.6 | 1425.0 | 1572.4 | |  | | 0 |  | 0 |  |  |  | 0 |  | 0 |  |  | |  |
| **Mixed** |  | 469 | 3528 | 1329.4 | 1223.0 | 1441.4 | |  | | 0 |  | 0 |  |  |  | 0 |  | 0 |  |  | |  |
| **Other** |  | 835 | 6277 | 1330.3 | 1250.2 | 1413.5 | |  | | 0 |  | 0 |  |  |  | 0 |  | 0 |  |  | |  |
| **Unknown** |  | 1583 | 10606 | 1492.6 | 1424.5 | 1562.6 | |  | | 0 |  | 0 |  |  |  | 0 |  | 0 |  |  | |  |
| **Index of Multiple Deprivation** | | | | | | | | | | | | | | | | | | | | |  |  |
| **1 (least)** |  | 9425 | 61945 | 1521.5 | 1494.7 | 1548.6 | |  | | 0 |  | 0 |  |  |  | 0 |  | 0 |  |  | |  |
| **2** |  | 8359 | 58360 | 1432.3 | 1405.3 | 1459.6 | |  | | 0 |  | 0 |  |  |  | 0 |  | 0 |  |  | |  |
| **3** |  | 8148 | 58758 | 1386.7 | 1359.9 | 1413.8 | |  | | 0 |  | 0 |  |  |  | 0 |  | 0 |  |  | |  |
| **4** |  | 7579 | 59246 | 1279.2 | 1253.2 | 1305.7 | |  | | 0 |  | 0 |  |  |  | 0 |  | 0 |  |  | |  |
| **5 (most)** |  | 7589 | 59612 | 1273.1 | 1247.1 | 1299.3 | |  | | 0 |  | 0 |  |  |  | 0 |  | 0 |  |  | |  |
| **Missing** |  | 29 | 234 | 1239.3 | 871.8 | 1691.5 | |  | | 0 |  | 0 |  |  |  | 0 |  | 0 |  |  | |  |

AP, All Pregnancies; AP24+, All Pregnancies with gestational age ≥24 weeks; LR, Low-Risk pregnancies; n, number of pregnancies in the specified category; CI, confidence interval; HES, Hospital Episode Statistics.

**Table S9.4. Termination**

|  |  | **AP cohort** | | | | |  | | **AP24+ cohort** | | | | | |  | **LR cohort** | | | | | |  |
| --- | --- | --- | --- | --- | --- | --- | --- | --- | --- | --- | --- | --- | --- | --- | --- | --- | --- | --- | --- | --- | --- | --- |
|  |  | **n** | **Population under study** | **Incidence/**  **10 000** | **95% CI** | | |  | | **n** | **Population under study** | **Incidence/**  **10 000** | **95% CI** | |  | **n** | **Population under study** | **Incidence/**  **10 000** | **95% CI** | | |  |
| **Year of pregnancy start date** | | | | | | | | | | | | | | | | | | | | |  |  |
| **2004*** |  | 331 | 16027 | 206.5 | 185.1 | 229.7 | |  | | 8 | 14041 | 5.7 | 2.5 | 11.2 |  | 5 | 10040 | 5.0 | 1.6 | 11.6 | |  |
| **2005** |  | 1608 | 27609 | 582.4 | 554.9 | 610.9 | |  | | 16 | 19220 | 8.3 | 4.8 | 13.5 |  | 11 | 13456 | 8.2 | 4.1 | 14.6 | |  |
| **2006** |  | 1580 | 28221 | 559.9 | 533.2 | 587.4 | |  | | 6 | 19706 | 3.0 | 1.1 | 6.6 |  | *#* | *#* | *#* | *#* | *#* | |  |
| **2007** |  | 1702 | 29076 | 585.4 | 558.6 | 613.0 | |  | | 5 | 20268 | 2.5 | 0.8 | 5.8 |  | *#* | *#* | *#* | *#* | *#* | |  |
| **2008** |  | 1793 | 29761 | 602.5 | 575.5 | 630.3 | |  | | 9 | 20382 | 4.4 | 2.1 | 8.2 |  | *#* | *#* | *#* | *#* | *#* | |  |
| **2009** |  | 1698 | 29342 | 578.7 | 552.1 | 606.2 | |  | | 15 | 20125 | 7.5 | 4.2 | 12.3 |  | 7 | 13421 | 5.2 | 2.1 | 10.7 | |  |
| **2010** |  | 1551 | 27983 | 554.3 | 527.6 | 581.9 | |  | | 12 | 19479 | 6.2 | 3.2 | 10.6 |  | 6 | 12763 | 4.7 | 1.7 | 10.2 | |  |
| **2011** |  | 1380 | 27209 | 507.2 | 481.3 | 534.1 | |  | | 7 | 19073 | 3.7 | 1.5 | 7.6 |  | 6 | 12349 | 4.9 | 1.8 | 10.6 | |  |
| **2012** |  | 1190 | 24661 | 482.5 | 456.1 | 510.1 | |  | | 11 | 16888 | 6.5 | 3.3 | 11.7 |  | 5 | 10688 | 4.7 | 1.5 | 10.9 | |  |
| **2013** |  | 1018 | 20812 | 489.1 | 460.1 | 519.5 | |  | | 8 | 14269 | 5.6 | 2.4 | 11.0 |  | *#* | *#* | *#* | *#* | *#* | |  |
| **2014** |  | 774 | 16166 | 478.8 | 446.2 | 513.0 | |  | | 5 | 10959 | 4.6 | 1.5 | 10.6 |  | *#* | *#* | *#* | *#* | *#* | |  |
| **2015** |  | 519 | 11514 | 450.8 | 413.5 | 490.3 | |  | | 5 | 7903 | 6.3 | 2.1 | 14.8 |  | *#* | *#* | *#* | *#* | *#* | |  |
| **2016** |  | 337 | 8580 | 392.8 | 352.4 | 436.4 | |  | | *#* | *#* | *#* | *#* | *#* |  | *#* | *#* | *#* | *#* | *#* | |  |
| **2017°** |  | 109 | 1194 | 912.9 | 754.6 | 1091.9 | |  | | 0 | 61 | 0 | 0 | 0 |  | 0 | 25 | 0 | 0 | 0 | |  |
| **Age at pregnancy start date** | | | | | | | | | | | | | | | | | | | | |  |  |
| **18–24** |  | 6438 | 64282 | 1001.5 | 977.9 | 1025.5 | |  | | 25 | 40102 | 6.2 | 4.1 | 9.1 |  | 16 | 25770 | 6.2 | 3.6 | 10.1 | |  |
| **25–29** |  | 3314 | 73199 | 452.7 | 437.8 | 468.0 | |  | | 28 | 54429 | 5.1 | 3.5 | 7.3 |  | 15 | 35746 | 4.2 | 2.4 | 6.8 | |  |
| **30–34** |  | 2662 | 85923 | 309.8 | 298.2 | 321.7 | |  | | 26 | 65489 | 4.0 | 2.6 | 5.8 |  | 18 | 44713 | 4.0 | 2.4 | 6.3 | |  |
| **35–39** |  | 2157 | 56947 | 378.8 | 362.8 | 395.3 | |  | | 25 | 39366 | 6.4 | 4.2 | 9.2 |  | 11 | 26076 | 4.2 | 2.2 | 7.4 | |  |
| **40–45** |  | 1019 | 17804 | 572.3 | 537.2 | 609.0 | |  | | 6 | 8942 | 6.7 | 2.5 | 14.4 |  | *#* | *#* | *#* | *#* | 10.2 | |  |
| **Ethnicity (from HES data)** | | | | | | | | | | | | | | | | | | | | |  |  |
| **White** |  | 13424 | 252166 | 532.3 | 523.4 | 541.5 | |  | | 100 | 178527 | 5.6 | 4.6 | 6.8 |  | 58 | 115810 | 5.0 | 3.8 | 6.4 | |  |
| **Asian** |  | 488 | 16757 | 291.2 | 265.5 | 318.7 | |  | | 6 | 12054 | 5.0 | 1.6 | 11.5 |  | *#* | *#* | *#* | *#* | 4.6 | |  |
| **Black** |  | 462 | 8821 | 523.8 | 477.2 | 573.4 | |  | | *#* | *#* | *#* | *#* | *#* |  | *#* | *#* | *#* | *#* | 15.6 | |  |
| **Mixed** |  | 231 | 3528 | 654.8 | 572.2 | 745.2 | |  | | 0 | 2320 | 0 | 0 | 0 |  | 0 | 1518 | 0 | 0 | 0 | |  |
| **Other** |  | 284 | 6277 | 452.4 | 401.5 | 507.8 | |  | | *#* | *#* | *#* | *#* | *#* |  | 0 | 3342 | 0 | 0 | 0 | |  |
| **Unknown** |  | 701 | 10606 | 660.9 | 613.0 | 711.5 | |  | | *#* | *#* | *#* | *#* | *#* |  | *#* | *#* | *#* | *#* | 14.3 | |  |
| **Index of Multiple Deprivation** | | | | | | | | | | | | | | | | | | | | |  |  |
| **1 (least)** |  | 2191 | 61945 | 353.7 | 338.6 | 369.3 | |  | | 20 | 44577 | 4.5 | 2.8 | 6.8 |  | 11 | 30724 | 3.6 | 1.9 | 6.2 | |  |
| **2** |  | 2474 | 58360 | 423.9 | 407.2 | 441.1 | |  | | 20 | 41240 | 4.8 | 3.0 | 7.4 |  | 13 | 27772 | 4.7 | 2.5 | 8.1 | |  |
| **3** |  | 3034 | 58758 | 516.4 | 497.9 | 535.3 | |  | | 23 | 40881 | 5.6 | 3.6 | 8.4 |  | 14 | 27328 | 5.1 | 2.8 | 8.6 | |  |
| **4** |  | 3590 | 59246 | 605.9 | 586.4 | 625.9 | |  | | 19 | 40908 | 4.6 | 3.0 | 6.9 |  | 10 | 26604 | 3.8 | 2.0 | 6.4 | |  |
| **5 (most)** |  | 4280 | 59612 | 718.0 | 696.9 | 739.4 | |  | | 28 | 40573 | 6.9 | 4.6 | 9.9 |  | 13 | 25415 | 5.1 | 2.7 | 8.8 | |  |
| **Missing** |  | 21 | 234 | 897.4 | 554.0 | 1357.0 | |  | | 0 | 149 | 0 | 0 | 0 |  | 0 | 89 | 0 | 0 | 0 | |  |

AP, All Pregnancies; AP24+, All Pregnancies with gestational age ≥24 weeks; LR, Low-Risk pregnancies; n, number of pregnancies in the specified category; CI, confidence interval; #, results where an individual cell count is <5 (to maintain confidentiality); HES, Hospital Episode Statistics.

**Table S9.5. Miscarriage or termination (composite endpoint)**

|  |  | **AP cohort** | | | | |  | | **AP24+ cohort** | | | | | |  | **LR cohort** | | | | | |  |
| --- | --- | --- | --- | --- | --- | --- | --- | --- | --- | --- | --- | --- | --- | --- | --- | --- | --- | --- | --- | --- | --- | --- |
|  |  | **n** | **Population under study** | **Incidence/**  **10 000** | **95% CI** | | |  | | **n** | **Population under study** | **Incidence/**  **10 000** | **95% CI** | |  | **n** | **Population under study** | **Incidence/**  **10 000** | **95% CI** | | |  |
| **Year of pregnancy start date** | | | | | | | | | | | | | | | | | | | | |  |  |
| **2004*** |  | 498 | 16027 | 310.7 | 284.4 | 338.8 | |  | | 0 | 14041 | 0 |  |  |  | 0 | 10040 | 0 |  |  | |  |
| **2005** |  | 2131 | 27609 | 771.8 | 740.3 | 804.3 | |  | | 0 | 19220 | 0 |  |  |  | 0 | 13456 | 0 |  |  | |  |
| **2006** |  | 2299 | 28221 | 814.6 | 782.7 | 847.5 | |  | | 0 | 19706 | 0 |  |  |  | 0 | 13527 | 0 |  |  | |  |
| **2007** |  | 2298 | 29076 | 790.3 | 759.4 | 822.1 | |  | | 0 | 20268 | 0 |  |  |  | 0 | 13830 | 0 |  |  | |  |
| **2008** |  | 2427 | 29761 | 815.5 | 784.4 | 847.4 | |  | | 0 | 20382 | 0 |  |  |  | 0 | 13709 | 0 |  |  | |  |
| **2009** |  | 2347 | 29342 | 799.9 | 768.8 | 831.8 | |  | | 0 | 20125 | 0 |  |  |  | 0 | 13421 | 0 |  |  | |  |
| **2010** |  | 2099 | 27983 | 750.1 | 719.3 | 781.8 | |  | | 0 | 19479 | 0 |  |  |  | 0 | 12763 | 0 |  |  | |  |
| **2011** |  | 2098 | 27209 | 771.1 | 739.3 | 803.7 | |  | | 0 | 19073 | 0 |  |  |  | 0 | 12349 | 0 |  |  | |  |
| **2012** |  | 1913 | 24661 | 775.7 | 742.3 | 810.1 | |  | | 0 | 16888 | 0 |  |  |  | 0 | 10688 | 0 |  |  | |  |
| **2013** |  | 1508 | 20812 | 724.6 | 689.5 | 760.9 | |  | | 0 | 14269 | 0 |  |  |  | 0 | 8982 | 0 |  |  | |  |
| **2014** |  | 1304 | 16166 | 806.6 | 764.8 | 850.0 | |  | | 0 | 10959 | 0 |  |  |  | 0 | 6697 | 0 |  |  | |  |
| **2015** |  | 882 | 11514 | 766.0 | 717.8 | 816.5 | |  | | 0 | 7903 | 0 |  |  |  | 0 | 4856 | 0 |  |  | |  |
| **2016** |  | 575 | 8580 | 670.2 | 617.7 | 725.7 | |  | | 0 | 5954 | 0 |  |  |  | 0 | 3589 | 0 |  |  | |  |
| **2017°** |  | 230 | 1194 | 1926.3 | 1704.8 | 2163.2 | |  | | 0 | 61 | 0 |  |  |  | 0 | 25 | 0 |  |  | |  |
| **Age at pregnancy start date** | | | | | | | | | | | | | | | | | | | | |  |  |
| **18–24** |  | 9266 | 64282 | 1441.5 | 1413.2 | 1470.0 | |  | | 0 | 40102 | 0 |  |  |  | 0 | 25770 | 0 |  |  | |  |
| **25–29** |  | 4872 | 73199 | 665.6 | 647.4 | 684.1 | |  | | 0 | 54429 | 0 |  |  |  | 0 | 35746 | 0 |  |  | |  |
| **30–34** |  | 3834 | 85923 | 446.2 | 432.1 | 460.6 | |  | | 0 | 65489 | 0 |  |  |  | 0 | 44713 | 0 |  |  | |  |
| **35–39** |  | 3143 | 56947 | 551.9 | 532.5 | 571.9 | |  | | 0 | 39366 | 0 |  |  |  | 0 | 26076 | 0 |  |  | |  |
| **40–45** |  | 1494 | 17804 | 839.1 | 796.4 | 883.4 | |  | | 0 | 8942 | 0 |  |  |  | 0 | 5627 | 0 |  |  | |  |
| **Ethnicity (from HES data)** | | | | | | | | | | | | | | | | | | | | |  |  |
| **White** |  | 16973 | 252166 | 673.1 | 662.9 | 683.4 | |  | | 0 | 178527 | 0 |  |  |  | 0 | 115810 | 0 |  |  | |  |
| **Asian** |  | 1164 | 16757 | 694.6 | 654.8 | 736.2 | |  | | 0 | 12054 | 0 |  |  |  | 0 | 8918 | 0 |  |  | |  |
| **Black** |  | 940 | 8821 | 1065.6 | 1000.4 | 1133.6 | |  | | 0 | 5672 | 0 |  |  |  | 0 | 4071 | 0 |  |  | |  |
| **Mixed** |  | 363 | 3528 | 1028.9 | 926.7 | 1138.3 | |  | | 0 | 2320 | 0 |  |  |  | 0 | 1518 | 0 |  |  | |  |
| **Other** |  | 465 | 6277 | 740.8 | 674.4 | 811.6 | |  | | 0 | 4360 | 0 |  |  |  | 0 | 3342 | 0 |  |  | |  |
| **Unknown** |  | 2704 | 10606 | 2549.5 | 2461.9 | 2638.6 | |  | | 0 | 5395 | 0 |  |  |  | 0 | 4273 | 0 |  |  | |  |
| **Index of Multiple Deprivation** | | | | | | | | | | | | | | | | | | | | |  |  |
| **1 (least)** |  | 3842 | 61945 | 620.2 | 600.0 | 641.0 | |  | | 0 | 44577 | 0 |  |  |  | 0 | 30724 | 0 |  |  | |  |
| **2** |  | 4201 | 58360 | 719.8 | 697.6 | 742.6 | |  | | 0 | 41240 | 0 |  |  |  | 0 | 27772 | 0 |  |  | |  |
| **3** |  | 4673 | 58758 | 795.3 | 772.3 | 818.7 | |  | | 0 | 40881 | 0 |  |  |  | 0 | 27328 | 0 |  |  | |  |
| **4** |  | 5013 | 59246 | 846.1 | 822.8 | 869.9 | |  | | 0 | 40908 | 0 |  |  |  | 0 | 26604 | 0 |  |  | |  |
| **5 (most)** |  | 4856 | 59612 | 814.6 | 791.9 | 837.7 | |  | | 0 | 40573 | 0 |  |  |  | 0 | 25415 | 0 |  |  | |  |
| **Missing** |  | 24 | 234 | 1025.6 | 656.0 | 1508.2 | |  | | 0 | 149 | 0 |  |  |  | 0 | 89 | 0 |  |  | |  |

AP, All Pregnancies; AP24+, All Pregnancies with gestational age ≥24 weeks; LR, Low-Risk pregnancies; n, number of pregnancies in the specified category; CI, confidence interval; HES, Hospital Episode Statistics.

**Table S9.6. Ectopic pregnancy**

|  |  | **AP cohort** | | | | |  | | **AP24+ cohort** | | | | | |  | **LR cohort** | | | | | |  |
| --- | --- | --- | --- | --- | --- | --- | --- | --- | --- | --- | --- | --- | --- | --- | --- | --- | --- | --- | --- | --- | --- | --- |
|  |  | **n** | **Population under study** | **Incidence/**  **10 000** | **95% CI** | | |  | | **n** | **Population under study** | **Incidence/**  **10 000** | **95% CI** | |  | **n** | **Population under study** | **Incidence/**  **10 000** | **95% CI** | | |  |
| **Year of pregnancy start date** | | | | | | | | | | | | | | | | | | | | |  |  |
| **2004*** |  | 44 | 16027 | 27.5 | 20.0 | 36.8 | |  | | 0 | 14041 | 0 |  |  |  | 0 | 10040 | 0 |  |  | |  |
| **2005** |  | 247 | 27609 | 89.5 | 78.8 | 101.2 | |  | | 0 | 19220 | 0 |  |  |  | 0 | 13456 | 0 |  |  | |  |
| **2006** |  | 252 | 28221 | 89.3 | 78.9 | 100.7 | |  | | 0 | 19706 | 0 |  |  |  | 0 | 13527 | 0 |  |  | |  |
| **2007** |  | 291 | 29076 | 100.1 | 89.1 | 112.0 | |  | | 0 | 20268 | 0 |  |  |  | 0 | 13830 | 0 |  |  | |  |
| **2008** |  | 281 | 29761 | 94.4 | 83.9 | 105.9 | |  | | 0 | 20382 | 0 |  |  |  | 0 | 13709 | 0 |  |  | |  |
| **2009** |  | 284 | 29342 | 96.8 | 86.0 | 108.6 | |  | | 0 | 20125 | 0 |  |  |  | 0 | 13421 | 0 |  |  | |  |
| **2010** |  | 282 | 27983 | 100.8 | 89.7 | 112.9 | |  | | 0 | 19479 | 0 |  |  |  | 0 | 12763 | 0 |  |  | |  |
| **2011** |  | 289 | 27209 | 106.2 | 94.5 | 118.9 | |  | | 0 | 19073 | 0 |  |  |  | 0 | 12349 | 0 |  |  | |  |
| **2012** |  | 259 | 24661 | 105.0 | 92.8 | 118.4 | |  | | 0 | 16888 | 0 |  |  |  | 0 | 10688 | 0 |  |  | |  |
| **2013** |  | 232 | 20812 | 111.5 | 97.8 | 126.5 | |  | | 0 | 14269 | 0 |  |  |  | 0 | 8982 | 0 |  |  | |  |
| **2014** |  | 209 | 16166 | 129.3 | 112.6 | 147.7 | |  | | 0 | 10959 | 0 |  |  |  | 0 | 6697 | 0 |  |  | |  |
| **2015** |  | 154 | 11514 | 133.8 | 113.8 | 156.1 | |  | | 0 | 7903 | 0 |  |  |  | 0 | 4856 | 0 |  |  | |  |
| **2016** |  | 120 | 8580 | 139.9 | 116.7 | 166.2 | |  | | 0 | 5954 | 0 |  |  |  | 0 | 3589 | 0 |  |  | |  |
| **2017°** |  | 52 | 1194 | 435.5 | 327.3 | 566.7 | |  | | 0 | 61 | 0 |  |  |  | 0 | 25 | 0 |  |  | |  |
| **Age at pregnancy start date** | | | | | | | | | | | | | | | | | | | | |  |  |
| **18–24** |  | 412 | 64282 | 64.1 | 58.2 | 70.5 | |  | | 0 | 40102 | 0 |  |  |  | 0 | 25770 | 0 |  |  | |  |
| **25–29** |  | 703 | 73199 | 96.0 | 89.4 | 103.1 | |  | | 0 | 54429 | 0 |  |  |  | 0 | 35746 | 0 |  |  | |  |
| **30–34** |  | 957 | 85923 | 111.4 | 104.7 | 118.4 | |  | | 0 | 65489 | 0 |  |  |  | 0 | 44713 | 0 |  |  | |  |
| **35–39** |  | 699 | 56947 | 122.7 | 114.0 | 131.9 | |  | | 0 | 39366 | 0 |  |  |  | 0 | 26076 | 0 |  |  | |  |
| **40–45** |  | 225 | 17804 | 126.4 | 110.3 | 144.1 | |  | | 0 | 8942 | 0 |  |  |  | 0 | 5627 | 0 |  |  | |  |
| **Ethnicity (from HES data)** | | | | | | | | | | | | | | | | | | | | |  |  |
| **White** |  | 2500 | 252166 | 99.1 | 95.4 | 103.0 | |  | | 0 | 178527 | 0 |  |  |  | 0 | 115810 | 0 |  |  | |  |
| **Asian** |  | 149 | 16757 | 88.9 | 75.6 | 103.9 | |  | | 0 | 12054 | 0 |  |  |  | 0 | 8918 | 0 |  |  | |  |
| **Black** |  | 138 | 8821 | 156.4 | 131.7 | 184.4 | |  | | 0 | 5672 | 0 |  |  |  | 0 | 4071 | 0 |  |  | |  |
| **Mixed** |  | 38 | 3528 | 107.7 | 76.4 | 147.5 | |  | | 0 | 2320 | 0 |  |  |  | 0 | 1518 | 0 |  |  | |  |
| **Other** |  | 88 | 6277 | 140.2 | 113.2 | 171.6 | |  | | 0 | 4360 | 0 |  |  |  | 0 | 3342 | 0 |  |  | |  |
| **Unknown** |  | 83 | 10606 | 78.3 | 62.4 | 96.9 | |  | | 0 | 5395 | 0 |  |  |  | 0 | 4273 | 0 |  |  | |  |
| **Index of Multiple Deprivation** | | | | | | | | | | | | | | | | | | | | |  |  |
| **1 (least)** |  | 545 | 61945 | 88.0 | 81.1 | 95.3 | |  | | 0 | 44577 | 0 |  |  |  | 0 | 30724 | 0 |  |  | |  |
| **2** |  | 590 | 58360 | 101.1 | 93.5 | 109.2 | |  | | 0 | 41240 | 0 |  |  |  | 0 | 27772 | 0 |  |  | |  |
| **3** |  | 582 | 58758 | 99.1 | 91.3 | 107.3 | |  | | 0 | 40881 | 0 |  |  |  | 0 | 27328 | 0 |  |  | |  |
| **4** |  | 649 | 59246 | 109.5 | 101.4 | 118.2 | |  | | 0 | 40908 | 0 |  |  |  | 0 | 26604 | 0 |  |  | |  |
| **5 (most)** |  | 625 | 59612 | 104.8 | 96.8 | 113.4 | |  | | 0 | 40573 | 0 |  |  |  | 0 | 25415 | 0 |  |  | |  |
| **Missing** |  | 5 | 234 | 213.7 | 68.5 | 496.2 | |  | | 0 | 149 | 0 |  |  |  | 0 | 89 | 0 |  |  | |  |

AP, All Pregnancies; AP24+, All Pregnancies with gestational age ≥24 weeks; LR, Low-Risk pregnancies; n, number of pregnancies in the specified category; CI, confidence interval; HES, Hospital Episode Statistics.

**Table S9.7. Preterm delivery**

|  |  | **AP cohort** | | | | |  | | **AP24+ cohort** | | | | | |  | **LR cohort** | | | | | |  |
| --- | --- | --- | --- | --- | --- | --- | --- | --- | --- | --- | --- | --- | --- | --- | --- | --- | --- | --- | --- | --- | --- | --- |
|  |  | **n** | **Population under study** | **Incidence/**  **10 000** | **95% CI** | | |  | | **n** | **Population under study** | **Incidence/**  **10 000** | **95% CI** | |  | **n** | **Population under study** | **Incidence/**  **10 000** | **95% CI** | | |  |
| **Year of pregnancy start date** | | | | | | | | | | | | | | | | | | | | |  |  |
| **2004*** |  | 971 | 16027 | 605.9 | 569.4 | 643.9 | |  | | 948 | 14041 | 675.2 | 634.2 | 717.9 |  | 616 | 10040 | 613.5 | 567.4 | 662.3 | |  |
| **2005** |  | 1423 | 27609 | 515.4 | 489.5 | 542.3 | |  | | 1395 | 19220 | 725.8 | 689.5 | 763.4 |  | 900 | 13456 | 668.8 | 627.2 | 712.4 | |  |
| **2006** |  | 1474 | 28221 | 522.3 | 496.5 | 549.0 | |  | | 1447 | 19706 | 734.3 | 698.3 | 771.6 |  | 921 | 13527 | 680.9 | 639.0 | 724.6 | |  |
| **2007** |  | 1433 | 29076 | 492.8 | 468.1 | 518.5 | |  | | 1402 | 20268 | 691.7 | 657.2 | 727.5 |  | 907 | 13830 | 655.8 | 615.2 | 698.3 | |  |
| **2008** |  | 1518 | 29761 | 510.1 | 485.2 | 535.8 | |  | | 1485 | 20382 | 728.6 | 693.3 | 765.1 |  | 940 | 13709 | 685.7 | 643.9 | 729.3 | |  |
| **2009** |  | 1367 | 29342 | 465.9 | 441.9 | 490.8 | |  | | 1341 | 20125 | 666.3 | 632.3 | 701.7 |  | 820 | 13421 | 611.0 | 571.1 | 652.8 | |  |
| **2010** |  | 1257 | 27983 | 449.2 | 425.1 | 474.3 | |  | | 1232 | 19479 | 632.5 | 598.7 | 667.6 |  | 740 | 12763 | 579.8 | 539.9 | 621.8 | |  |
| **2011** |  | 1406 | 27209 | 516.7 | 490.6 | 543.9 | |  | | 1378 | 19073 | 722.5 | 686.1 | 760.1 |  | 827 | 12349 | 669.7 | 626.2 | 715.2 | |  |
| **2012** |  | 1503 | 24661 | 609.5 | 579.7 | 640.3 | |  | | 1431 | 16888 | 847.3 | 805.8 | 890.4 |  | 848 | 10688 | 793.4 | 742.8 | 846.3 | |  |
| **2013** |  | 1185 | 20812 | 569.4 | 538.1 | 601.9 | |  | | 1119 | 14269 | 784.2 | 740.6 | 829.6 |  | 630 | 8982 | 701.4 | 649.4 | 756.2 | |  |
| **2014** |  | 987 | 16166 | 610.5 | 573.9 | 648.8 | |  | | 946 | 10959 | 863.2 | 811.3 | 917.3 |  | 525 | 6697 | 783.9 | 720.6 | 850.9 | |  |
| **2015** |  | 757 | 11514 | 657.5 | 612.7 | 704.4 | |  | | 731 | 7903 | 925.0 | 862.0 | 991.0 |  | 384 | 4856 | 790.8 | 716.4 | 870.2 | |  |
| **2016** |  | 607 | 8580 | 707.5 | 653.9 | 764.0 | |  | | 583 | 5954 | 979.2 | 904.9 | 1057.4 |  | 310 | 3589 | 863.8 | 773.8 | 960.5 | |  |
| **2017°** |  | 43 | 1194 | 360.1 | 261.3 | 482.9 | |  | | 38 | 61 | 6229.5 | 4884.5 | 7448.8 |  | 12 | 25 | 4800.0 | 2739.4 | 6911.7 | |  |
| **Age at pregnancy start date** | | | | | | | | | | | | | | | | | | | | |  |  |
| **18–24** |  | 3087 | 64282 | 480.2 | 463.6 | 497.3 | |  | | 2985 | 40102 | 744.4 | 718.5 | 770.9 |  | 1788 | 25770 | 693.8 | 662.7 | 726.0 | |  |
| **25–29** |  | 4135 | 73199 | 564.9 | 547.9 | 582.2 | |  | | 4021 | 54429 | 738.8 | 716.6 | 761.4 |  | 2454 | 35746 | 686.5 | 660.1 | 713.6 | |  |
| **30–34** |  | 4872 | 85923 | 567.0 | 551.3 | 583.0 | |  | | 4736 | 65489 | 723.2 | 703.2 | 743.5 |  | 2970 | 44713 | 664.2 | 641.1 | 688.0 | |  |
| **35–39** |  | 3030 | 56947 | 532.1 | 513.4 | 551.3 | |  | | 2955 | 39366 | 750.6 | 724.6 | 777.4 |  | 1718 | 26076 | 658.8 | 628.7 | 690.0 | |  |
| **40–45** |  | 807 | 17804 | 453.3 | 422.4 | 485.7 | |  | | 779 | 8942 | 871.2 | 813.5 | 931.6 |  | 450 | 5627 | 799.7 | 730.1 | 873.8 | |  |
| **Ethnicity (from HES data)** | | | | | | | | | | | | | | | | | | | | |  |  |
| **White** |  | 13311 | 252166 | 527.9 | 518.9 | 537.0 | |  | | 12956 | 178527 | 725.7 | 713.4 | 738.2 |  | 7670 | 115810 | 662.3 | 647.7 | 677.1 | |  |
| **Asian** |  | 1173 | 16757 | 700.0 | 660.7 | 740.9 | |  | | 1134 | 12054 | 940.8 | 887.9 | 995.7 |  | 786 | 8918 | 881.4 | 822.0 | 943.6 | |  |
| **Black** |  | 536 | 8821 | 607.6 | 556.6 | 661.9 | |  | | 510 | 5672 | 899.2 | 823.9 | 978.9 |  | 316 | 4071 | 776.2 | 693.4 | 865.5 | |  |
| **Mixed** |  | 196 | 3528 | 555.6 | 481.6 | 637.2 | |  | | 186 | 2320 | 801.7 | 694.6 | 919.6 |  | 113 | 1518 | 744.4 | 617.5 | 888.0 | |  |
| **Other** |  | 338 | 6277 | 538.5 | 483.0 | 598.3 | |  | | 322 | 4360 | 738.5 | 661.9 | 821.0 |  | 214 | 3342 | 640.3 | 559.4 | 729.0 | |  |
| **Unknown** |  | 377 | 10606 | 355.5 | 320.3 | 393.3 | |  | | 368 | 5395 | 682.1 | 614.4 | 754.8 |  | 281 | 4273 | 657.6 | 583.1 | 738.5 | |  |
| **Index of Multiple Deprivation** | | | | | | | | | | | | | | | | | | | | |  |  |
| **1 (least)** |  | 2923 | 61945 | 471.9 | 454.7 | 489.5 | |  | | 2852 | 44577 | 639.8 | 616.6 | 663.6 |  | 1874 | 30724 | 609.9 | 582.7 | 638.0 | |  |
| **2** |  | 2980 | 58360 | 510.6 | 492.3 | 529.5 | |  | | 2878 | 41240 | 697.9 | 672.7 | 723.7 |  | 1765 | 27772 | 635.5 | 606.3 | 665.7 | |  |
| **3** |  | 3280 | 58758 | 558.2 | 539.3 | 577.6 | |  | | 3186 | 40881 | 779.3 | 752.9 | 806.4 |  | 1930 | 27328 | 706.2 | 675.5 | 737.9 | |  |
| **4** |  | 3199 | 59246 | 540.0 | 521.3 | 559.1 | |  | | 3121 | 40908 | 762.9 | 736.7 | 789.8 |  | 1846 | 26604 | 693.9 | 662.9 | 725.8 | |  |
| **5 (most)** |  | 3534 | 59612 | 592.8 | 573.4 | 612.7 | |  | | 3424 | 40573 | 843.9 | 816.2 | 872.2 |  | 1956 | 25415 | 769.6 | 736.3 | 803.9 | |  |
| **Missing** |  | 15 | 234 | 641.0 | 349.9 | 1062.3 | |  | | 15 | 149 | 1006.7 | 555.2 | 1643.7 |  | 9 | 89 | 1011.2 | 452.1 | 1882.7 | |  |

AP, All Pregnancies; AP24+, All Pregnancies with gestational age ≥24 weeks; LR, Low-Risk pregnancies; n, number of pregnancies in the specified category; CI, confidence interval; HES, Hospital Episode Statistics.

**Table S9.8. Maternal sepsis**

|  |  | **AP cohort** | | | | |  | | **AP24+ cohort** | | | | | |  | **LR cohort** | | | | | |  |
| --- | --- | --- | --- | --- | --- | --- | --- | --- | --- | --- | --- | --- | --- | --- | --- | --- | --- | --- | --- | --- | --- | --- |
|  |  | **n** | **Population under study** | **Incidence/**  **10 000** | **95% CI** | | |  | | **n** | **Population under study** | **Incidence/**  **10 000** | **95% CI** | |  | **n** | **Population under study** | **Incidence/**  **10 000** | **95% CI** | | |  |
| **Year of pregnancy start date** | | | | | | | | | | | | | | | | | | | | |  |  |
| **2004*** |  | 8 | 16027 | 5 | 2.2 | 9.8 | |  | | 8 | 14041 | 5.7 | 2.5 | 11.2 |  | 6 | 10040 | 6 | 2.2 | 13 | |  |
| **2005** |  | 15 | 27609 | 5.4 | 3 | 9 | |  | | 11 | 19220 | 5.7 | 2.9 | 10.2 |  | 7 | 13456 | 5.2 | 2.1 | 10.7 | |  |
| **2006** |  | 12 | 28221 | 4.3 | 2.2 | 7.4 | |  | | 10 | 19706 | 5.1 | 2.4 | 9.3 |  | 5 | 13527 | 3.7 | 1.2 | 8.6 | |  |
| **2007** |  | 12 | 29076 | 4.1 | 2.1 | 7.2 | |  | | 11 | 20268 | 5.4 | 2.7 | 9.7 |  | 6 | 13830 | 4.3 | 1.6 | 9.4 | |  |
| **2008** |  | 11 | 29761 | 3.7 | 1.9 | 6.6 | |  | | 10 | 20382 | 4.9 | 2.4 | 9 |  | 5 | 13709 | 3.6 | 1.2 | 8.5 | |  |
| **2009** |  | 22 | 29342 | 7.5 | 4.7 | 11.3 | |  | | 15 | 20125 | 7.5 | 4.2 | 12.3 |  | 9 | 13421 | 6.7 | 3.1 | 12.7 | |  |
| **2010** |  | 20 | 27983 | 7.1 | 4.3 | 11.1 | |  | | 16 | 19479 | 8.2 | 4.7 | 13.3 |  | 6 | 12763 | 4.7 | 1.7 | 10.2 | |  |
| **2011** |  | 17 | 27209 | 6.2 | 3.6 | 10.1 | |  | | 13 | 19073 | 6.8 | 3.6 | 11.7 |  | 7 | 12349 | 5.7 | 2.3 | 11.7 | |  |
| **2012** |  | 18 | 24661 | 7.3 | 4.3 | 11.6 | |  | | 13 | 16888 | 7.7 | 4.1 | 13.2 |  | 6 | 10688 | 5.6 | 2.1 | 12.2 | |  |
| **2013** |  | 21 | 20812 | 10.1 | 6.3 | 15.4 | |  | | 18 | 14269 | 12.6 | 7.5 | 19.9 |  | 10 | 8982 | 11.1 | 5.3 | 20.5 | |  |
| **2014** |  | 17 | 16166 | 10.5 | 6.1 | 17 | |  | | 12 | 10959 | 10.9 | 5.7 | 19.1 |  | 10 | 6697 | 14.9 | 7.2 | 27.4 | |  |
| **2015** |  | 22 | 11514 | 19.1 | 11.9 | 29.1 | |  | | 18 | 7903 | 22.8 | 13.5 | 36 |  | 13 | 4856 | 26.8 | 14.3 | 45.7 | |  |
| **2016** |  | 22 | 8580 | 25.6 | 16.1 | 38.7 | |  | | 18 | 5954 | 30.2 | 17.9 | 47.7 |  | 13 | 3589 | 36.2 | 19.3 | 61.9 | |  |
| **2017°** |  | *#* | *#* | *#* | *#* | *#* | |  | | *#* | *#* | *#* | *#* | *#* |  | 0 | 25 | 0 |  |  | |  |
| **Age at pregnancy start date** | | | | | | | | | | | | | | | | | | | | |  |  |
| **18–24** |  | 45 | 64282 | 7 | 5.1 | 9.4 | |  | | 38 | 40102 | 9.5 | 6.7 | 13 |  | 22 | 25770 | 8.5 | 5.3 | 13.1 | |  |
| **25–29** |  | 63 | 73199 | 8.6 | 6.6 | 11 | |  | | 46 | 54429 | 8.5 | 6.2 | 11.3 |  | 28 | 35746 | 7.8 | 5.2 | 11.3 | |  |
| **30–34** |  | 66 | 85923 | 7.7 | 5.9 | 9.8 | |  | | 55 | 65489 | 8.4 | 6.3 | 11 |  | 34 | 44713 | 7.6 | 5.2 | 10.7 | |  |
| **35–39** |  | 33 | 56947 | 5.8 | 3.9 | 8.2 | |  | | 25 | 39366 | 6.4 | 4.1 | 9.4 |  | 15 | 26076 | 5.8 | 3.1 | 9.7 | |  |
| **40–45** |  | 13 | 17804 | 7.3 | 3.9 | 12.5 | |  | | 10 | 8942 | 11.2 | 5.6 | 19.9 |  | # | # | # | # | # | |  |
| **Ethnicity** | | | | | | | | | | | | | | | | | | | | |  |  |
| **White** |  | 180 | 252166 | 7.1 | 6.1 | 8.3 | |  | | 139 | 178527 | 7.8 | 6.5 | 9.2 |  | 79 | 115810 | 6.8 | 5.4 | 8.6 | |  |
| **Asian** |  | 13 | 16757 | 7.8 | 4.2 | 13 | |  | | 12 | 12054 | 10 | 5.2 | 17.3 |  | 9 | 8918 | 10.1 | 4.6 | 19.3 | |  |
| **Black** |  | 15 | 8821 | 17 | 9 | 29.2 | |  | | 14 | 5672 | 24.7 | 13.1 | 42.2 |  | 10 | 4071 | 24.6 | 11.4 | 46.2 | |  |
| **Mixed** |  | *#* | *#* | *#* | *#* | *#* | |  | | *#* | *#* | *#* | *#* | *#* |  | 0 | 1518 | 0 |  |  | |  |
| **Other** |  | 7 | 6277 | 11.2 | 4.4 | 23.4 | |  | | 5 | 4360 | 11.5 | 3.8 | 26.3 |  | 3 | 3342 | 9 | 2.3 | 23.4 | |  |
| **Unknown** |  | *#* | *#* | *#* | *#* | *#* | |  | | *#* | *#* | *#* | *#* | *#* |  | # | # | # | # | # | |  |
| **Index of Multiple Deprivation** | | | | | | | | | | | | | | | | | | | | |  |  |
| **1 (least)** |  | 37 | 61945 | 6 | 4.1 | 8.4 | |  | | 27 | 44577 | 6.1 | 3.8 | 9.1 |  | 19 | 30724 | 6.2 | 3.6 | 9.9 | |  |
| **2** |  | 42 | 58360 | 7.2 | 5.2 | 9.7 | |  | | 36 | 41240 | 8.7 | 6.2 | 12 |  | 21 | 27772 | 7.6 | 4.7 | 11.5 | |  |
| **3** |  | 49 | 58758 | 8.3 | 6.1 | 11.1 | |  | | 36 | 40881 | 8.8 | 6.2 | 12.2 |  | 22 | 27328 | 8.1 | 5 | 12.3 | |  |
| **4** |  | 48 | 59246 | 8.1 | 6.1 | 10.6 | |  | | 39 | 40908 | 9.5 | 6.8 | 13 |  | 24 | 26604 | 9 | 5.7 | 13.6 | |  |
| **5 (most)** |  | 44 | 59612 | 7.4 | 5.2 | 10.1 | |  | | 36 | 40573 | 8.9 | 6.1 | 12.4 |  | 17 | 25415 | 6.7 | 3.8 | 10.8 | |  |
| **Missing** |  | 0 | 234 | 0 |  |  | |  | | 0 | 149 | 0 |  |  |  | 0 | 89 | 0 |  |  | |  |

AP, All Pregnancies; AP24+, All Pregnancies with gestational age ≥24 weeks; LR, Low-Risk pregnancies; n, number of pregnancies in the specified category; CI, confidence interval; #, results where an individual cell count is <5 (to maintain confidentiality).

If a particular pregnancy-related event of interest occurred several times for the same pregnancy, it was only counted once for that pregnancy.

**Table S9.9. Vaginal or intrauterine hemorrhage**

|  |  | **AP cohort** | | | | |  | | **AP24+ cohort** | | | | | |  | **LR cohort** | | | | | |  |
| --- | --- | --- | --- | --- | --- | --- | --- | --- | --- | --- | --- | --- | --- | --- | --- | --- | --- | --- | --- | --- | --- | --- |
|  |  | **n** | **Population under study** | **Incidence/**  **10 000** | **95% CI** | | |  | | **n** | **Population under study** | **Incidence/**  **10 000** | **95% CI** | |  | **n** | **Population under study** | **Incidence/**  **10 000** | **95% CI** | | |  |
| **Year of pregnancy start date** | | | | | | | | | | | | | | | | | | | | |  |  |
| **2004*** |  | 1245 | 16027 | 776.8 | 735.8 | 819.3 | |  | | 1141 | 14041 | 812.6 | 767.9 | 859 |  | 772 | 10040 | 768.9 | 717.5 | 822.8 | |  |
| **2005** |  | 2002 | 27609 | 725.1 | 694.9 | 756.3 | |  | | 1570 | 19220 | 816.9 | 778.5 | 856.5 |  | 1009 | 13456 | 749.9 | 705.9 | 795.7 | |  |
| **2006** |  | 1952 | 28221 | 691.7 | 662.3 | 721.9 | |  | | 1582 | 19706 | 802.8 | 765.2 | 841.6 |  | 1035 | 13527 | 765.1 | 720.9 | 811.2 | |  |
| **2007** |  | 2005 | 29076 | 689.6 | 660.7 | 719.3 | |  | | 1639 | 20268 | 808.7 | 771.5 | 847 |  | 996 | 13830 | 720.2 | 677.6 | 764.5 | |  |
| **2008** |  | 2059 | 29761 | 691.8 | 663.3 | 721.3 | |  | | 1631 | 20382 | 800.2 | 763.3 | 838.3 |  | 961 | 13709 | 701 | 658.8 | 745 | |  |
| **2009** |  | 2056 | 29342 | 700.7 | 671.7 | 730.5 | |  | | 1614 | 20125 | 802 | 764.8 | 840.4 |  | 987 | 13421 | 735.4 | 691.8 | 780.9 | |  |
| **2010** |  | 1968 | 27983 | 703.3 | 673.6 | 733.9 | |  | | 1572 | 19479 | 807 | 769.2 | 846.1 |  | 984 | 12763 | 771 | 725.3 | 818.6 | |  |
| **2011** |  | 1925 | 27209 | 707.5 | 677.3 | 738.6 | |  | | 1539 | 19073 | 806.9 | 768.6 | 846.4 |  | 889 | 12349 | 719.9 | 674.9 | 766.9 | |  |
| **2012** |  | 1731 | 24661 | 701.9 | 670.3 | 734.6 | |  | | 1369 | 16888 | 810.6 | 769.9 | 852.8 |  | 791 | 10688 | 740.1 | 691.1 | 791.4 | |  |
| **2013** |  | 1395 | 20812 | 670.3 | 636.6 | 705.2 | |  | | 1085 | 14269 | 760.4 | 717.4 | 805.1 |  | 626 | 8982 | 696.9 | 645.1 | 751.6 | |  |
| **2014** |  | 1070 | 16166 | 661.9 | 624 | 701.3 | |  | | 832 | 10959 | 759.2 | 710.3 | 810.4 |  | 441 | 6697 | 658.5 | 600.2 | 720.6 | |  |
| **2015** |  | 709 | 11514 | 615.8 | 572.5 | 661.3 | |  | | 569 | 7903 | 720 | 664 | 779.2 |  | 309 | 4856 | 636.3 | 569.3 | 708.7 | |  |
| **2016** |  | 616 | 8580 | 717.9 | 664.2 | 774.6 | |  | | 494 | 5954 | 829.7 | 760.9 | 902.6 |  | 252 | 3589 | 702.1 | 620.7 | 790.6 | |  |
| **2017°** |  | 61 | 1194 | 510.9 | 392.8 | 651.8 | |  | | 12 | 61 | 1967.2 | 1053.4 | 3196 |  | *#* | *#* | *#* | *#* | *#* | |  |
| **Age at pregnancy start date** | | | | | | | | | | | | | | | | | | | | |  |  |
| **18–24** |  | 4874 | 64282 | 758.2 | 737.9 | 779 | |  | | 3943 | 40102 | 983.2 | 954 | 1013.1 |  | 2290 | 25770 | 888.6 | 853.9 | 924.3 | |  |
| **25–29** |  | 5420 | 73199 | 740.4 | 721.5 | 759.7 | |  | | 4453 | 54429 | 818.1 | 795.1 | 841.6 |  | 2659 | 35746 | 743.9 | 716.7 | 771.8 | |  |
| **30–34** |  | 5847 | 85923 | 680.5 | 663.6 | 697.7 | |  | | 4804 | 65489 | 733.6 | 713.5 | 754 |  | 3003 | 44713 | 671.6 | 648.4 | 695.4 | |  |
| **35–39** |  | 3629 | 56947 | 637.3 | 617.2 | 657.7 | |  | | 2815 | 39366 | 715.1 | 689.7 | 741.1 |  | 1743 | 26076 | 668.4 | 638.2 | 699.7 | |  |
| **40–45** |  | 1024 | 17804 | 575.2 | 541 | 610.8 | |  | | 634 | 8942 | 709 | 656.4 | 764.5 |  | 360 | 5627 | 639.8 | 577 | 707.2 | |  |
| **Ethnicity** | | | | | | | | | | | | | | | | | | | | |  |  |
| **White** |  | 18028 | 252166 | 714.9 | 704.8 | 725.2 | |  | | 14546 | 178527 | 814.8 | 801.9 | 827.8 |  | 8611 | 115810 | 743.5 | 728.3 | 759.1 | |  |
| **Asian** |  | 1324 | 16757 | 790.1 | 749.2 | 832.5 | |  | | 984 | 12054 | 816.3 | 767.1 | 867.6 |  | 676 | 8918 | 758 | 702.7 | 816.3 | |  |
| **Black** |  | 512 | 8821 | 580.4 | 531.4 | 632.5 | |  | | 384 | 5672 | 677 | 611.6 | 747.1 |  | 263 | 4071 | 646 | 571.7 | 726.9 | |  |
| **Mixed** |  | 236 | 3528 | 668.9 | 587 | 758.5 | |  | | 186 | 2320 | 801.7 | 692.5 | 922 |  | 104 | 1518 | 685.1 | 562.6 | 824.8 | |  |
| **Other** |  | 417 | 6277 | 664.3 | 604.4 | 728.2 | |  | | 335 | 4360 | 768.3 | 691.7 | 850.7 |  | 245 | 3342 | 733.1 | 647.3 | 826.3 | |  |
| **Unknown** |  | 277 | 10606 | 261.2 | 231.9 | 293 | |  | | 214 | 5395 | 396.7 | 345.2 | 453.4 |  | 156 | 4273 | 365.1 | 310 | 426.8 | |  |
| **Index of Multiple Deprivation** | | | | | | | | | | | | | | | | | | | | |  |  |
| **1 (least)** |  | 3879 | 61945 | 626.2 | 607 | 645.8 | |  | | 3207 | 44577 | 719.4 | 695.2 | 744.2 |  | 2093 | 30724 | 681.2 | 652.8 | 710.5 | |  |
| **2** |  | 3818 | 58360 | 654.2 | 634.1 | 674.8 | |  | | 3071 | 41240 | 744.7 | 719.1 | 770.9 |  | 1901 | 27772 | 684.5 | 654.7 | 715.2 | |  |
| **3** |  | 3926 | 58758 | 668.2 | 647.9 | 688.9 | |  | | 3162 | 40881 | 773.5 | 747.4 | 800.2 |  | 1909 | 27328 | 698.6 | 668.1 | 729.9 | |  |
| **4** |  | 4065 | 59246 | 686.1 | 665.7 | 706.9 | |  | | 3306 | 40908 | 808.2 | 781.6 | 835.4 |  | 1963 | 26604 | 737.9 | 706.4 | 770.3 | |  |
| **5 (most)** |  | 5091 | 59612 | 854 | 831.5 | 876.9 | |  | | 3891 | 40573 | 959 | 930.1 | 988.5 |  | 2184 | 25415 | 859.3 | 824.6 | 895.1 | |  |
| **Missing** |  | 15 | 234 | 641 | 359.8 | 1042 | |  | | 12 | 149 | 805.4 | 418.5 | 1374.3 |  | 5 | 89 | 561.8 | 163.9 | 1341.1 | |  |

AP, All Pregnancies; AP24+, All Pregnancies with gestational age ≥24 weeks; LR, Low-Risk pregnancies; n, number of pregnancies in the specified category; CI, confidence interval; #, results where an individual cell count is <5 (to maintain confidentiality).

If a particular pregnancy-related event of interest occurred several times for the same pregnancy, it was only counted once for that pregnancy.

**Table S9.10. Pre-eclampsia**

|  |  | **AP cohort** | | | | |  | | **AP24+ cohort** | | | | | |  | **LR cohort** | | | | | |  |
| --- | --- | --- | --- | --- | --- | --- | --- | --- | --- | --- | --- | --- | --- | --- | --- | --- | --- | --- | --- | --- | --- | --- |
|  |  | **n** | **Population under study** | **Incidence/**  **10 000** | **95% CI** | | |  | | **n** | **Population under study** | **Incidence/**  **10 000** | **95% CI** | |  | **n** | **Population under study** | **Incidence/**  **10 000** | **95% CI** | | |  |
| **Year of pregnancy start date** | | | | | | | | | | | | | | | | | | | | |  |  |
| **2004*** |  | 323 | 16027 | 201.5 | 180.3 | 224.5 | |  | | 315 | 14041 | 224.3 | 200.5 | 250.2 |  | 206 | 10040 | 205.2 | 178.3 | 234.8 | |  |
| **2005** |  | 420 | 27609 | 152.1 | 137.9 | 167.4 | |  | | 396 | 19220 | 206 | 186.4 | 227.1 |  | 262 | 13456 | 194.7 | 172 | 219.5 | |  |
| **2006** |  | 445 | 28221 | 157.7 | 143.4 | 173 | |  | | 424 | 19706 | 215.2 | 195.4 | 236.4 |  | 252 | 13527 | 186.3 | 164.2 | 210.5 | |  |
| **2007** |  | 432 | 29076 | 148.6 | 134.9 | 163.3 | |  | | 419 | 20268 | 206.7 | 187.6 | 227.3 |  | 249 | 13830 | 180 | 158.5 | 203.6 | |  |
| **2008** |  | 447 | 29761 | 150.2 | 136.5 | 164.9 | |  | | 421 | 20382 | 206.6 | 187.5 | 227 |  | 236 | 13709 | 172.1 | 151 | 195.3 | |  |
| **2009** |  | 481 | 29342 | 163.9 | 149.6 | 179.2 | |  | | 455 | 20125 | 226.1 | 206 | 247.6 |  | 270 | 13421 | 201.2 | 178.1 | 226.4 | |  |
| **2010** |  | 408 | 27983 | 145.8 | 132 | 160.6 | |  | | 383 | 19479 | 196.6 | 177.6 | 217.1 |  | 218 | 12763 | 170.8 | 149 | 194.8 | |  |
| **2011** |  | 402 | 27209 | 147.7 | 133.7 | 162.9 | |  | | 388 | 19073 | 203.4 | 183.9 | 224.5 |  | 214 | 12349 | 173.3 | 151 | 197.9 | |  |
| **2012** |  | 404 | 24661 | 163.8 | 148.2 | 180.6 | |  | | 376 | 16888 | 222.6 | 200.9 | 246 |  | 225 | 10688 | 210.5 | 184.1 | 239.5 | |  |
| **2013** |  | 275 | 20812 | 132.1 | 117 | 148.7 | |  | | 260 | 14269 | 182.2 | 160.9 | 205.5 |  | 146 | 8982 | 162.5 | 137.4 | 190.9 | |  |
| **2014** |  | 246 | 16166 | 152.2 | 133.7 | 172.4 | |  | | 222 | 10959 | 202.6 | 177 | 230.7 |  | 125 | 6697 | 186.7 | 155.6 | 222 | |  |
| **2015** |  | 166 | 11514 | 144.2 | 123.2 | 167.7 | |  | | 153 | 7903 | 193.6 | 164.4 | 226.4 |  | 83 | 4856 | 170.9 | 136.4 | 211.3 | |  |
| **2016** |  | 131 | 8580 | 152.7 | 127.7 | 181.1 | |  | | 127 | 5954 | 213.3 | 178.1 | 253.3 |  | 72 | 3589 | 200.6 | 157.3 | 252 | |  |
| **2017°** |  | *#* | *#* | *#* | *#* | *#* | |  | | *#* | *#* | *#* | *#* | *#* |  | 0 | 25 | 0 |  |  | |  |
| **Age at pregnancy start date** | | | | | | | | | | | | | | | | | | | | |  |  |
| **18–24** |  | 995 | 64282 | 154.8 | 144.9 | 165.2 | |  | | 950 | 40102 | 236.9 | 221.6 | 252.9 |  | 559 | 25770 | 216.9 | 198.9 | 236.1 | |  |
| **25–29** |  | 1158 | 73199 | 158.2 | 149 | 167.8 | |  | | 1099 | 54429 | 201.9 | 190.1 | 214.3 |  | 659 | 35746 | 184.4 | 170.4 | 199.1 | |  |
| **30–34** |  | 1290 | 85923 | 150.1 | 141.9 | 158.7 | |  | | 1213 | 65489 | 185.2 | 174.9 | 196 |  | 751 | 44713 | 168 | 156.1 | 180.4 | |  |
| **35–39** |  | 894 | 56947 | 157 | 146.7 | 167.8 | |  | | 847 | 39366 | 215.2 | 201 | 230 |  | 467 | 26076 | 179.1 | 163.4 | 195.9 | |  |
| **40–45** |  | 244 | 17804 | 137 | 119.9 | 155.9 | |  | | 231 | 8942 | 258.3 | 226.1 | 293.8 |  | 122 | 5627 | 216.8 | 180.1 | 258.7 | |  |
| **Ethnicity** | | | | | | | | | | | | | | | | | | | | |  |  |
| **White** |  | 3962 | 252166 | 157.1 | 152 | 162.3 | |  | | 3758 | 178527 | 210.5 | 203.6 | 217.6 |  | 2173 | 115810 | 187.6 | 179.6 | 196 | |  |
| **Asian** |  | 242 | 16757 | 144.4 | 126.6 | 164.1 | |  | | 231 | 12054 | 191.6 | 167.4 | 218.4 |  | 157 | 8918 | 176 | 149.1 | 206.5 | |  |
| **Black** |  | 159 | 8821 | 180.3 | 151.9 | 212.3 | |  | | 148 | 5672 | 260.9 | 219.2 | 308.1 |  | 87 | 4071 | 213.7 | 169.5 | 265.8 | |  |
| **Mixed** |  | 56 | 3528 | 158.7 | 117.1 | 210.1 | |  | | 52 | 2320 | 224.1 | 165 | 297.1 |  | 27 | 1518 | 177.9 | 114.1 | 263.8 | |  |
| **Other** |  | 79 | 6277 | 125.9 | 99.7 | 156.7 | |  | | 73 | 4360 | 167.4 | 131.5 | 210 |  | 54 | 3342 | 161.6 | 121.8 | 210 | |  |
| **Unknown** |  | 83 | 10606 | 78.3 | 62.4 | 96.9 | |  | | 78 | 5395 | 144.6 | 114.4 | 180.1 |  | 60 | 4273 | 140.4 | 106.9 | 181 | |  |
| **Index of Multiple Deprivation** | | | | | | | | | | | | | | | | | | | | |  |  |
| **1 (least)** |  | 901 | 61945 | 145.5 | 135.7 | 155.7 | |  | | 866 | 44577 | 194.3 | 181.2 | 208 |  | 546 | 30724 | 177.7 | 162.7 | 193.7 | |  |
| **2** |  | 914 | 58360 | 156.6 | 146.4 | 167.4 | |  | | 860 | 41240 | 208.5 | 194.6 | 223.2 |  | 508 | 27772 | 182.9 | 167.1 | 199.8 | |  |
| **3** |  | 902 | 58758 | 153.5 | 143.2 | 164.3 | |  | | 856 | 40881 | 209.4 | 195.2 | 224.3 |  | 512 | 27328 | 187.4 | 170.9 | 204.9 | |  |
| **4** |  | 902 | 59246 | 152.2 | 141.9 | 163.1 | |  | | 855 | 40908 | 209 | 194.8 | 224 |  | 493 | 26604 | 185.3 | 168.8 | 203 | |  |
| **5 (most)** |  | 960 | 59612 | 161 | 150.4 | 172.2 | |  | | 901 | 40573 | 222.1 | 207.3 | 237.6 |  | 498 | 25415 | 195.9 | 178.5 | 214.7 | |  |
| **Missing** |  | *#* | *#* | *#* | *#* | *#* | |  | | *#* | *#* | *#* | *#* | *#* |  | *#* | *#* | *#* | *#* | *#* | |  |

AP, All Pregnancies; AP24+, All Pregnancies with gestational age ≥24 weeks; LR, Low-Risk pregnancies; n, number of pregnancies in the specified category; CI, confidence interval; #, results where an individual cell count is <5 (to maintain confidentiality).

If a particular pregnancy-related event of interest occurred several times for the same pregnancy, it was only counted once for that pregnancy.

**Table S9.11. Eclampsia**

|  |  | **AP cohort** | | | | |  | | **AP24+ cohort** | | | | | |  | **LR cohort** | | | | | |  |
| --- | --- | --- | --- | --- | --- | --- | --- | --- | --- | --- | --- | --- | --- | --- | --- | --- | --- | --- | --- | --- | --- | --- |
|  |  | **n** | **Population under study** | **Incidence/**  **10 000** | **95% CI** | | |  | | **n** | **Population under study** | **Incidence/**  **10 000** | **95% CI** | |  | **n** | **Population under study** | **Incidence/**  **10 000** | **95% CI** | | |  |
| **Year of pregnancy start date** | | | | | | | | | | | | | | | | | | | | |  |  |
| **2004*** |  | 19 | 16027 | 11.9 | 7.1 | 18.5 | |  | | 17 | 14041 | 12.1 | 7.1 | 19.4 |  | 11 | 10040 | 11 | 5.5 | 19.6 | |  |
| **2005** |  | 25 | 27609 | 9.1 | 5.8 | 13.5 | |  | | 24 | 19220 | 12.5 | 8 | 18.6 |  | 13 | 13456 | 9.7 | 5.1 | 16.5 | |  |
| **2006** |  | 34 | 28221 | 12 | 8.3 | 16.9 | |  | | 34 | 19706 | 17.3 | 12 | 24.1 |  | 15 | 13527 | 11.1 | 6.2 | 18.3 | |  |
| **2007** |  | 20 | 29076 | 6.9 | 4.2 | 10.6 | |  | | 19 | 20268 | 9.4 | 5.6 | 14.6 |  | 11 | 13830 | 8 | 4 | 14.2 | |  |
| **2008** |  | 21 | 29761 | 7.1 | 4.4 | 10.8 | |  | | 20 | 20382 | 9.8 | 6 | 15.2 |  | 13 | 13709 | 9.5 | 5 | 16.2 | |  |
| **2009** |  | 20 | 29342 | 6.8 | 4.2 | 10.5 | |  | | 19 | 20125 | 9.4 | 5.7 | 14.7 |  | 11 | 13421 | 8.2 | 4.1 | 14.7 | |  |
| **2010** |  | 14 | 27983 | 5 | 2.7 | 8.5 | |  | | 12 | 19479 | 6.2 | 3.2 | 10.8 |  | 6 | 12763 | 4.7 | 1.7 | 10.2 | |  |
| **2011** |  | 15 | 27209 | 5.5 | 3.1 | 9.1 | |  | | 13 | 19073 | 6.8 | 3.6 | 11.7 |  | 8 | 12349 | 6.5 | 2.8 | 12.8 | |  |
| **2012** |  | 18 | 24661 | 7.3 | 4.3 | 11.6 | |  | | 17 | 16888 | 10.1 | 5.9 | 16.1 |  | 9 | 10688 | 8.4 | 3.9 | 16 | |  |
| **2013** |  | 14 | 20812 | 6.7 | 3.6 | 11.4 | |  | | 14 | 14269 | 9.8 | 5.4 | 16.5 |  | 6 | 8982 | 6.7 | 2.5 | 14.5 | |  |
| **2014** |  | 15 | 16166 | 9.3 | 5.1 | 15.4 | |  | | 15 | 10959 | 13.7 | 7.7 | 22.6 |  | 7 | 6697 | 10.5 | 4.2 | 21.5 | |  |
| **2015** |  | *#* | *#* | *#* | *#* | *#* | |  | | *#* | *#* | *#* | *#* | *#* |  | *#* | *#* | *#* | *#* | *#* | |  |
| **2016** |  | 5 | 8580 | 5.8 | 1.8 | 13.8 | |  | | 5 | 5954 | 8.4 | 2.7 | 19.6 |  | *#* | *#* | *#* | *#* | *#* | |  |
| **2017°** |  | 0 | 1194 | 0 |  |  | |  | | 0 | 61 | 0 |  |  |  | 0 | 25 | 0 |  |  | |  |
| **Age at pregnancy start date** | | | | | | | | | | | | | | | | | | | | |  |  |
| **18–24** |  | 42 | 64282 | 6.5 | 4.7 | 8.9 | |  | | 40 | 40102 | 10 | 7.1 | 13.6 |  | 18 | 25770 | 7 | 4.2 | 10.9 | |  |
| **25–29** |  | 57 | 73199 | 7.8 | 5.9 | 10.1 | |  | | 54 | 54429 | 9.9 | 7.5 | 12.9 |  | 28 | 35746 | 7.8 | 5.2 | 11.3 | |  |
| **30–34** |  | 59 | 85923 | 6.9 | 5.2 | 8.9 | |  | | 54 | 65489 | 8.2 | 6.2 | 10.7 |  | 34 | 44713 | 7.6 | 5.3 | 10.6 | |  |
| **35–39** |  | 50 | 56947 | 8.8 | 6.4 | 11.7 | |  | | 50 | 39366 | 12.7 | 9.4 | 16.8 |  | 25 | 26076 | 9.6 | 6.1 | 14.3 | |  |
| **40–45** |  | 16 | 17804 | 9 | 4.9 | 15 | |  | | 15 | 8942 | 16.8 | 9.3 | 28 |  | 9 | 5627 | 16 | 7.2 | 30.7 | |  |
| **Ethnicity** | | | | | | | | | | | | | | | | | | | | |  |  |
| **White** |  | 189 | 252166 | 7.5 | 6.4 | 8.7 | |  | | 181 | 178527 | 10.1 | 8.7 | 11.8 |  | 96 | 115810 | 8.3 | 6.7 | 10.1 | |  |
| **Asian** |  | 13 | 16757 | 7.8 | 4.3 | 13 | |  | | 13 | 12054 | 10.8 | 5.8 | 18.3 |  | 8 | 8918 | 9 | 3.5 | 18.7 | |  |
| **Black** |  | 9 | 8821 | 10.2 | 4 | 21.3 | |  | | 7 | 5672 | 12.3 | 4.6 | 26.5 |  | 5 | 4071 | 12.3 | 3.8 | 29.6 | |  |
| **Mixed** |  | *#* | *#* | *#* | *#* | *#* | |  | | *#* | *#* | *#* | *#* | *#* |  | 0 | 1518 | 0 |  |  | |  |
| **Other** |  | 5 | 6277 | 8 | 2.5 | 18.8 | |  | | 5 | 4360 | 11.5 | 3.9 | 25.9 |  | *#* | *#* | *#* | *#* | *#* | |  |
| **Unknown** |  | 6 | 10606 | 5.7 | 2.3 | 11.7 | |  | | 5 | 5395 | 9.3 | 3.2 | 21 |  | *#* | *#* | *#* | *#* | *#* | |  |
| **Index of Multiple Deprivation** | | | | | | | | | | | | | | | | | | | | |  |  |
| **1 (least)** |  | 59 | 61945 | 9.5 | 7.2 | 12.4 | |  | | 58 | 44577 | 13 | 9.9 | 16.8 |  | 34 | 30724 | 11.1 | 7.7 | 15.5 | |  |
| **2** |  | 41 | 58360 | 7 | 4.9 | 9.7 | |  | | 38 | 41240 | 9.2 | 6.4 | 12.8 |  | 22 | 27772 | 7.9 | 4.9 | 12 | |  |
| **3** |  | 45 | 58758 | 7.7 | 5.4 | 10.5 | |  | | 42 | 40881 | 10.3 | 7.4 | 13.9 |  | 20 | 27328 | 7.3 | 4.5 | 11.3 | |  |
| **4** |  | 41 | 59246 | 6.9 | 4.9 | 9.5 | |  | | 40 | 40908 | 9.8 | 7 | 13.2 |  | 21 | 26604 | 7.9 | 4.8 | 12.2 | |  |
| **5 (most)** |  | 38 | 59612 | 6.4 | 4.5 | 8.8 | |  | | 35 | 40573 | 8.6 | 6 | 12.1 |  | 17 | 25415 | 6.7 | 3.8 | 10.9 | |  |
| **Missing** |  | 0 | 234 | 0 |  |  | |  | | 0 | 149 | 0 |  |  |  | 0 | 89 | 0 |  |  | |  |

AP, All Pregnancies; AP24+, All Pregnancies with gestational age ≥24 weeks; LR, Low-Risk pregnancies; n, number of pregnancies in the specified category; CI, confidence interval; #, results where an individual cell count is <5 (to maintain confidentiality).

If a particular pregnancy-related event of interest occurred several times for the same pregnancy, it was only counted once for that pregnancy.

**Table S9.12. Pregnancy-related hypertension**

|  |  | **AP cohort** | | | | |  | | **AP24+ cohort** | | | | | |  | **LR cohort** | | | | | |  |
| --- | --- | --- | --- | --- | --- | --- | --- | --- | --- | --- | --- | --- | --- | --- | --- | --- | --- | --- | --- | --- | --- | --- |
|  |  | **n** | **Population under study** | **Incidence/**  **10 000** | **95% CI** | | |  | | **n** | **Population under study** | **Incidence/**  **10 000** | **95% CI** | |  | **n** | **Population under study** | **Incidence/**  **10 000** | **95% CI** | | |  |
| **Year of pregnancy start date** | | | | | | | | | | | | | | | | | | | | |  |  |
| **2004*** |  | 75 | 16027 | 46.8 | 36.8 | 58.6 | |  | | 70 | 14041 | 49.9 | 38.9 | 62.9 |  | 40 | 10040 | 39.8 | 28.5 | 54.2 | |  |
| **2005** |  | 94 | 27609 | 34 | 27.5 | 41.7 | |  | | 91 | 19220 | 47.3 | 38.1 | 58.1 |  | 50 | 13456 | 37.2 | 27.6 | 49 | |  |
| **2006** |  | 90 | 28221 | 31.9 | 25.6 | 39.2 | |  | | 87 | 19706 | 44.1 | 35.4 | 54.4 |  | 44 | 13527 | 32.5 | 23.6 | 43.6 | |  |
| **2007** |  | 112 | 29076 | 38.5 | 31.6 | 46.4 | |  | | 109 | 20268 | 53.8 | 44.2 | 64.8 |  | 59 | 13830 | 42.7 | 32.5 | 55 | |  |
| **2008** |  | 114 | 29761 | 38.3 | 31.6 | 46 | |  | | 108 | 20382 | 53 | 43.5 | 63.9 |  | 56 | 13709 | 40.8 | 30.9 | 53 | |  |
| **2009** |  | 108 | 29342 | 36.8 | 30.1 | 44.5 | |  | | 99 | 20125 | 49.2 | 40 | 59.9 |  | 51 | 13421 | 38 | 28.3 | 49.9 | |  |
| **2010** |  | 120 | 27983 | 42.9 | 35.5 | 51.3 | |  | | 115 | 19479 | 59 | 48.8 | 70.8 |  | 62 | 12763 | 48.6 | 37.3 | 62.2 | |  |
| **2011** |  | 111 | 27209 | 40.8 | 33.6 | 49.1 | |  | | 108 | 19073 | 56.6 | 46.5 | 68.3 |  | 44 | 12349 | 35.6 | 25.9 | 47.8 | |  |
| **2012** |  | 108 | 24661 | 43.8 | 35.9 | 52.9 | |  | | 98 | 16888 | 58 | 47.1 | 70.7 |  | 43 | 10688 | 40.2 | 29.1 | 54.2 | |  |
| **2013** |  | 72 | 20812 | 34.6 | 27.1 | 43.5 | |  | | 68 | 14269 | 47.7 | 37 | 60.4 |  | 33 | 8982 | 36.7 | 25.3 | 51.6 | |  |
| **2014** |  | 80 | 16166 | 49.5 | 39.2 | 61.7 | |  | | 76 | 10959 | 69.3 | 54.7 | 86.7 |  | 39 | 6697 | 58.2 | 41.4 | 79.5 | |  |
| **2015** |  | 49 | 11514 | 42.6 | 31.3 | 56.5 | |  | | 48 | 7903 | 60.7 | 44.8 | 80.5 |  | 16 | 4856 | 32.9 | 18.8 | 53.5 | |  |
| **2016** |  | 41 | 8580 | 47.8 | 34.1 | 65.1 | |  | | 37 | 5954 | 62.1 | 43.8 | 85.6 |  | 19 | 3589 | 52.9 | 31.9 | 82.6 | |  |
| **2017°** |  | # | # | # | # | # | |  | | 0 | 61 | 0 |  |  |  | 0 | 25 | 0 |  |  | |  |
| **Age at pregnancy start date** | | | | | | | | | | | | | | | | | | | | |  |  |
| **18–24** |  | 160 | 64282 | 24.9 | 21 | 29.3 | |  | | 148 | 40102 | 36.9 | 31 | 43.6 |  | 78 | 25770 | 30.3 | 23.7 | 38.1 | |  |
| **25–29** |  | 259 | 73199 | 35.4 | 31.1 | 40.1 | |  | | 251 | 54429 | 46.1 | 40.5 | 52.3 |  | 123 | 35746 | 34.4 | 28.5 | 41.1 | |  |
| **30–34** |  | 382 | 85923 | 44.5 | 40 | 49.3 | |  | | 364 | 65489 | 55.6 | 50 | 61.6 |  | 186 | 44713 | 41.6 | 35.8 | 48.1 | |  |
| **35–39** |  | 289 | 56947 | 50.7 | 44.9 | 57.1 | |  | | 274 | 39366 | 69.6 | 61.6 | 78.4 |  | 135 | 26076 | 51.8 | 43.4 | 61.3 | |  |
| **40–45** |  | 85 | 17804 | 47.7 | 37.9 | 59.3 | |  | | 77 | 8942 | 86.1 | 68.1 | 107.4 |  | 34 | 5627 | 60.4 | 41.7 | 84.7 | |  |
| **Ethnicity** | | | | | | | | | | | | | | | | | | | | |  |  |
| **White** |  | 968 | 252166 | 38.4 | 35.9 | 41 | |  | | 928 | 178527 | 52 | 48.6 | 55.5 |  | 458 | 115810 | 39.5 | 35.9 | 43.4 | |  |
| **Asian** |  | 70 | 16757 | 41.8 | 32.3 | 53.1 | |  | | 61 | 12054 | 50.6 | 38.4 | 65.5 |  | 35 | 8918 | 39.2 | 26.8 | 55.4 | |  |
| **Black** |  | 79 | 8821 | 89.6 | 70.1 | 112.8 | |  | | 71 | 5672 | 125.2 | 96.8 | 159.2 |  | 30 | 4071 | 73.7 | 48.4 | 107.5 | |  |
| **Mixed** |  | 10 | 3528 | 28.3 | 13.3 | 52.9 | |  | | 9 | 2320 | 38.8 | 16.5 | 77.2 |  | 5 | 1518 | 32.9 | 9.9 | 80 | |  |
| **Other** |  | 22 | 6277 | 35 | 22 | 53 | |  | | 21 | 4360 | 48.2 | 29.7 | 73.7 |  | 10 | 3342 | 29.9 | 13.6 | 56.9 | |  |
| **Unknown** |  | 26 | 10606 | 24.5 | 16 | 35.9 | |  | | 24 | 5395 | 44.5 | 28.3 | 66.6 |  | 18 | 4273 | 42.1 | 24.6 | 67.2 | |  |
| **Index of Multiple Deprivation** | | | | | | | | | | | | | | | | | | | | |  |  |
| **1 (least)** |  | 259 | 61945 | 41.8 | 36.8 | 47.3 | |  | | 250 | 44577 | 56.1 | 49.3 | 63.6 |  | 138 | 30724 | 44.9 | 37.7 | 53.1 | |  |
| **2** |  | 240 | 58360 | 41.1 | 35.8 | 47 | |  | | 225 | 41240 | 54.6 | 47.5 | 62.4 |  | 110 | 27772 | 39.6 | 32.3 | 48 | |  |
| **3** |  | 227 | 58758 | 38.6 | 33.5 | 44.3 | |  | | 216 | 40881 | 52.8 | 45.8 | 60.6 |  | 112 | 27328 | 41 | 33.6 | 49.6 | |  |
| **4** |  | 249 | 59246 | 42 | 36.7 | 47.9 | |  | | 237 | 40908 | 57.9 | 50.5 | 66.1 |  | 112 | 26604 | 42.1 | 34.4 | 51 | |  |
| **5 (most)** |  | 200 | 59612 | 33.6 | 28.9 | 38.8 | |  | | 186 | 40573 | 45.8 | 39.3 | 53.2 |  | 84 | 25415 | 33.1 | 26 | 41.4 | |  |
| **Missing** |  | 0 | 234 | 0 |  |  | |  | | 0 | 149 | 0 |  |  |  | 0 | 89 | 0 |  |  | |  |

AP, All Pregnancies; AP24+, All Pregnancies with gestational age ≥24 weeks; LR, Low-Risk pregnancies; n, number of pregnancies in the specified category; CI, confidence interval; #, results where an individual cell count is <5 (to maintain confidentiality).

If a particular pregnancy-related event of interest occurred several times for the same pregnancy, it was only counted once for that pregnancy.

**Table S9.13. Liver or biliary disease**

|  |  | **AP cohort** | | | | |  | | **AP24+ cohort** | | | | | |  | **LR cohort** | | | | | |  |
| --- | --- | --- | --- | --- | --- | --- | --- | --- | --- | --- | --- | --- | --- | --- | --- | --- | --- | --- | --- | --- | --- | --- |
|  |  | **n** | **Population under study** | **Incidence/**  **10 000** | **95% CI** | | |  | | **n** | **Population under study** | **Incidence/**  **10 000** | **95% CI** | |  | **n** | **Population under study** | **Incidence/**  **10 000** | **95% CI** | | |  |
| **Year of pregnancy start date** | | | | | | | | | | | | | | | | | | | | |  |  |
| **2004*** |  | *#* | *#* | *#* | *#* | *#* | |  | | *#* | *#* | *#* | *#* | *#* |  | 0 | 10040 | 0 |  |  | |  |
| **2005** |  | *#* | *#* | *#* | *#* | *#* | |  | | *#* | *#* | *#* | *#* | *#* |  | 0 | 13456 | 0 |  |  | |  |
| **2006** |  | *#* | *#* | *#* | *#* | *#* | |  | | *#* | *#* | *#* | *#* | *#* |  | *#* | *#* | *#* | *#* | *#* | |  |
| **2007** |  | 9 | 29076 | 3.1 | 1.4 | 5.8 | |  | | 8 | 20268 | 3.9 | 1.7 | 7.8 |  | *#* | *#* | *#* | *#* | *#* | |  |
| **2008** |  | *#* | *#* | *#* | *#* | *#* | |  | | *#* | *#* | *#* | *#* | *#* |  | 0 | 13709 | 0 |  |  | |  |
| **2009** |  | *#* | *#* | *#* | *#* | *#* | |  | | *#* | *#* | *#* | *#* | *#* |  | 0 | 13421 | 0 |  |  | |  |
| **2010** |  | *#* | *#* | *#* | *#* | *#* | |  | | *#* | *#* | *#* | *#* | *#* |  | *#* | *#* | *#* | *#* | *#* | |  |
| **2011** |  | *#* | *#* | *#* | *#* | *#* | |  | | *#* | *#* | *#* | *#* | *#* |  | *#* | *#* | *#* | *#* | *#* | |  |
| **2012** |  | *#* | *#* | *#* | *#* | *#* | |  | | *#* | *#* | *#* | *#* | *#* |  | *#* | *#* | *#* | *#* | *#* | |  |
| **2013** |  | *#* | *#* | *#* | *#* | *#* | |  | | *#* | *#* | *#* | *#* | *#* |  | 0 | 8982 | 0 |  |  | |  |
| **2014** |  | *#* | *#* | *#* | *#* | *#* | |  | | *#* | *#* | *#* | *#* | *#* |  | 0 | 6697 | 0 |  |  | |  |
| **2015** |  | *#* | *#* | *#* | *#* | *#* | |  | | *#* | *#* | *#* | *#* | *#* |  | 0 | 4856 | 0 |  |  | |  |
| **2016** |  | *#* | *#* | *#* | *#* | *#* | |  | | *#* | *#* | *#* | *#* | *#* |  | 0 | 3589 | 0 |  |  | |  |
| **2017°** |  | *#* | *#* | *#* | *#* | *#* | |  | | *#* | *#* | *#* | *#* | *#* |  | 0 | 25 | 0 |  |  | |  |
| **Age at pregnancy start date** | | | | | | | | | | | | | | | | | | | | |  |  |
| **18–24** |  | *#* | *#* | *#* | *#* | *#* | |  | | *#* | *#* | *#* | *#* | *#* |  | *#* | *#* | *#* | *#* | *#* | |  |
| **25–29** |  | 7 | 73199 | 1 | 0.4 | 2.1 | |  | | 6 | 54429 | 1.1 | 0.4 | 2.5 |  | *#* | *#* | *#* | *#* | *#* | |  |
| **30–34** |  | 14 | 85923 | 1.6 | 0.9 | 2.8 | |  | | 13 | 65489 | 2 | 1 | 3.5 |  | *#* | *#* | *#* | *#* | *#* | |  |
| **35–39** |  | 8 | 56947 | 1.4 | 0.6 | 2.7 | |  | | 7 | 39366 | 1.8 | 0.7 | 3.6 |  | *#* | *#* | *#* | *#* | *#* | |  |
| **40–45** |  | *#* | *#* | *#* | *#* | *#* | |  | | *#* | *#* | *#* | *#* | *#* |  | 0 | 5627 | 0 |  |  | |  |
| **Ethnicity** | | | | | | | | | | | | | | | | | | | | |  |  |
| **White** |  | 27 | 252166 | 1.1 | 0.7 | 1.6 | |  | | 25 | 178527 | 1.4 | 0.9 | 2.1 |  | *#* | *#* | *#* | *#* | *#* | |  |
| **Asian** |  | *#* | *#* | *#* | *#* | *#* | |  | | *#* | *#* | *#* | *#* | *#* |  | 0 | 8918 | 0 |  |  | |  |
| **Black** |  | *#* | *#* | *#* | *#* | *#* | |  | | *#* | *#* | *#* | *#* | *#* |  | *#* | *#* | *#* | *#* | *#* | |  |
| **Mixed** |  | *#* | *#* | *#* | *#* | *#* | |  | | *#* | *#* | *#* | *#* | *#* |  | 0 | 1518 | 0 |  |  | |  |
| **Other** |  | 5 | 6277 | 8 | 2.2 | 20 | |  | | 5 | 4360 | 11.5 | 3.5 | 27.6 |  | *#* | *#* | *#* | *#* | *#* | |  |
| **Unknown** |  | 0 | 10606 | 0 |  |  | |  | | 0 | 5395 | 0 |  |  |  | 0 | 4273 | 0 |  |  | |  |
| **Index of Multiple Deprivation** | | | | | | | | | | | | | | | | | | | | |  |  |
| **1 (least)** |  | *#* | *#* | *#* | *#* | *#* | |  | | *#* | *#* | *#* | *#* | *#* |  | *#* | *#* | *#* | *#* | *#* | |  |
| **2** |  | 5 | 58360 | 0.9 | 0.2 | 2.1 | |  | | 5 | 41240 | 1.2 | 0.4 | 2.9 |  | *#* | *#* | *#* | *#* | *#* | |  |
| **3** |  | 11 | 58758 | 1.9 | 0.9 | 3.4 | |  | | 9 | 40881 | 2.2 | 0.9 | 4.4 |  | *#* | *#* | *#* | *#* | *#* | |  |
| **4** |  | 13 | 59246 | 2.2 | 1.1 | 3.8 | |  | | 13 | 40908 | 3.2 | 1.6 | 5.6 |  | *#* | *#* | *#* | *#* | *#* | |  |
| **5 (most)** |  | *#* | *#* | *#* | *#* | *#* | |  | | *#* | *#* | *#* | *#* | *#* |  | *#* | *#* | *#* | *#* | *#* | |  |
| **Missing** |  | 0 | 234 | 0 |  |  | |  | | 0 | 149 | 0 |  |  |  | 0 | 89 | 0 |  |  | |  |

AP, All Pregnancies; AP24+, All Pregnancies with gestational age ≥24 weeks; LR, Low-Risk pregnancies; n, number of pregnancies in the specified category; CI, confidence interval; #, results where an individual cell count is <5 (to maintain confidentiality).

If a particular pregnancy-related event of interest occurred several times for the same pregnancy, it was only counted once for that pregnancy.

**Table S9.14. Premature/preterm labor**

|  |  | **AP cohort** | | | | |  | | **AP24+ cohort** | | | | | |  | **LR cohort** | | | | | |  |
| --- | --- | --- | --- | --- | --- | --- | --- | --- | --- | --- | --- | --- | --- | --- | --- | --- | --- | --- | --- | --- | --- | --- |
|  |  | **n** | **Population under study** | **Incidence/**  **10 000** | **95% CI** | | |  | | **n** | **Population under study** | **Incidence/**  **10 000** | **95% CI** | |  | **n** | **Population under study** | **Incidence/**  **10 000** | **95% CI** | | |  |
| **Year of pregnancy start date** | | | | | | | | | | | | | | | | | | | | |  |  |
| **2004*** |  | 405 | 16027 | 252.7 | 229 | 278.2 | |  | | 397 | 14041 | 282.7 | 256 | 311.5 |  | 260 | 10040 | 259 | 228.8 | 291.9 | |  |
| **2005** |  | 607 | 27609 | 219.9 | 202.8 | 238 | |  | | 583 | 19220 | 303.3 | 279.5 | 328.6 |  | 378 | 13456 | 280.9 | 253.7 | 310.2 | |  |
| **2006** |  | 654 | 28221 | 231.7 | 214.3 | 250.2 | |  | | 630 | 19706 | 319.7 | 295.6 | 345.2 |  | 387 | 13527 | 286.1 | 258.6 | 315.6 | |  |
| **2007** |  | 668 | 29076 | 229.7 | 212.8 | 247.7 | |  | | 637 | 20268 | 314.3 | 290.7 | 339.2 |  | 399 | 13830 | 288.5 | 261.2 | 317.8 | |  |
| **2008** |  | 685 | 29761 | 230.2 | 213.4 | 247.9 | |  | | 646 | 20382 | 316.9 | 293.3 | 341.9 |  | 394 | 13709 | 287.4 | 260.1 | 316.7 | |  |
| **2009** |  | 712 | 29342 | 242.7 | 225.3 | 261 | |  | | 672 | 20125 | 333.9 | 309.5 | 359.6 |  | 361 | 13421 | 269 | 242.3 | 297.7 | |  |
| **2010** |  | 683 | 27983 | 244.1 | 226.2 | 263 | |  | | 642 | 19479 | 329.6 | 305 | 355.6 |  | 367 | 12763 | 287.5 | 259.2 | 318 | |  |
| **2011** |  | 570 | 27209 | 209.5 | 192.7 | 227.3 | |  | | 529 | 19073 | 277.4 | 254.5 | 301.7 |  | 311 | 12349 | 251.8 | 224.9 | 281 | |  |
| **2012** |  | 439 | 24661 | 178 | 161.8 | 195.4 | |  | | 413 | 16888 | 244.6 | 221.8 | 269 |  | 238 | 10688 | 222.7 | 195.5 | 252.5 | |  |
| **2013** |  | 371 | 20812 | 178.3 | 160.7 | 197.2 | |  | | 349 | 14269 | 244.6 | 219.9 | 271.3 |  | 190 | 8982 | 211.5 | 182.8 | 243.5 | |  |
| **2014** |  | 285 | 16166 | 176.3 | 156.5 | 197.9 | |  | | 275 | 10959 | 250.9 | 222.5 | 282 |  | 140 | 6697 | 209 | 176.1 | 246.2 | |  |
| **2015** |  | 201 | 11514 | 174.6 | 151.3 | 200.4 | |  | | 196 | 7903 | 248 | 214.8 | 284.7 |  | 106 | 4856 | 218.3 | 179 | 263.4 | |  |
| **2016** |  | 125 | 8580 | 145.7 | 121.3 | 173.5 | |  | | 116 | 5954 | 194.8 | 161.3 | 233.1 |  | 58 | 3589 | 161.6 | 122.9 | 208.4 | |  |
| **2017°** |  | 12 | 1194 | 100.5 | 51.8 | 175.5 | |  | | 9 | 61 | 1475.4 | 692.2 | 2628.4 |  | # | # | # | # | # | |  |
| **Age at pregnancy start date** | | | | | | | | | | | | | | | | | | | | |  |  |
| **18–24** |  | 1365 | 64282 | 212.3 | 201.2 | 224 | |  | | 1299 | 40102 | 323.9 | 306.7 | 341.9 |  | 751 | 25770 | 291.4 | 271.1 | 312.8 | |  |
| **25–29** |  | 1641 | 73199 | 224.2 | 213.5 | 235.3 | |  | | 1561 | 54429 | 286.8 | 272.8 | 301.3 |  | 917 | 35746 | 256.5 | 240.2 | 273.6 | |  |
| **30–34** |  | 1854 | 85923 | 215.8 | 206 | 225.9 | |  | | 1751 | 65489 | 267.4 | 255 | 280.2 |  | 1055 | 44713 | 235.9 | 221.9 | 250.6 | |  |
| **35–39** |  | 1256 | 56947 | 220.6 | 208.5 | 233.2 | |  | | 1197 | 39366 | 304.1 | 287.3 | 321.6 |  | 710 | 26076 | 272.3 | 252.8 | 292.9 | |  |
| **40–45** |  | 301 | 17804 | 169.1 | 149.9 | 189.9 | |  | | 286 | 8942 | 319.8 | 284.1 | 358.7 |  | 157 | 5627 | 279 | 237.1 | 326 | |  |
| **Ethnicity** | | | | | | | | | | | | | | | | | | | | |  |  |
| **White** |  | 5469 | 252166 | 216.9 | 211 | 222.8 | |  | | 5232 | 178527 | 293.1 | 285.1 | 301.2 |  | 3012 | 115810 | 260.1 | 250.8 | 269.6 | |  |
| **Asian** |  | 441 | 16757 | 263.2 | 239.4 | 288.6 | |  | | 403 | 12054 | 334.3 | 303 | 367.9 |  | 283 | 8918 | 317.3 | 282 | 355.8 | |  |
| **Black** |  | 210 | 8821 | 238.1 | 206.3 | 273.2 | |  | | 190 | 5672 | 335 | 288.3 | 386.8 |  | 113 | 4071 | 277.6 | 228.3 | 334.1 | |  |
| **Mixed** |  | 83 | 3528 | 235.3 | 188.2 | 290.3 | |  | | 75 | 2320 | 323.3 | 256 | 402.3 |  | 48 | 1518 | 316.2 | 234.3 | 416.8 | |  |
| **Other** |  | 131 | 6277 | 208.7 | 174.7 | 247.2 | |  | | 114 | 4360 | 261.5 | 216.3 | 313 |  | 72 | 3342 | 215.4 | 169.1 | 270.3 | |  |
| **Unknown** |  | 83 | 10606 | 78.3 | 62.7 | 96.4 | |  | | 80 | 5395 | 148.3 | 117.9 | 184.1 |  | 62 | 4273 | 145.1 | 111.5 | 185.5 | |  |
| **Index of Multiple Deprivation** | | | | | | | | | | | | | | | | | | | | |  |  |
| **1 (least)** |  | 1068 | 61945 | 172.4 | 162.1 | 183.2 | |  | | 1025 | 44577 | 229.9 | 216 | 244.5 |  | 659 | 30724 | 214.5 | 198.4 | 231.5 | |  |
| **2** |  | 1218 | 58360 | 208.7 | 196.9 | 221 | |  | | 1156 | 41240 | 280.3 | 264.2 | 297.1 |  | 710 | 27772 | 255.7 | 237 | 275.3 | |  |
| **3** |  | 1249 | 58758 | 212.6 | 200.7 | 225 | |  | | 1180 | 40881 | 288.6 | 272.3 | 305.7 |  | 691 | 27328 | 252.9 | 234.2 | 272.6 | |  |
| **4** |  | 1301 | 59246 | 219.6 | 207.7 | 232 | |  | | 1230 | 40908 | 300.7 | 284.1 | 318 |  | 692 | 26604 | 260.1 | 241.1 | 280.2 | |  |
| **5 (most)** |  | 1574 | 59612 | 264 | 251 | 277.6 | |  | | 1496 | 40573 | 368.7 | 350.2 | 387.9 |  | 833 | 25415 | 327.8 | 305.9 | 350.7 | |  |
| **Missing** |  | 7 | 234 | 299.1 | 110 | 641.4 | |  | | 7 | 149 | 469.8 | 181.7 | 971.6 |  | 5 | 89 | 561.8 | 163.9 | 1341.1 | |  |

AP, All Pregnancies; AP24+, All Pregnancies with gestational age ≥24 weeks; LR, Low-Risk pregnancies; n, number of pregnancies in the specified category; CI, confidence interval; #, results where an individual cell count is <5 (to maintain confidentiality).

If a particular pregnancy-related event of interest occurred several times for the same pregnancy, it was only counted once for that pregnancy.

**Table S9.15. Labor protraction/arrest disorders**

|  |  | **AP cohort** | | | | |  | | **AP24+ cohort** | | | | | |  | **LR cohort** | | | | | |  |
| --- | --- | --- | --- | --- | --- | --- | --- | --- | --- | --- | --- | --- | --- | --- | --- | --- | --- | --- | --- | --- | --- | --- |
|  |  | **n** | **Population under study** | **Incidence/**  **10 000** | **95% CI** | | |  | | **n** | **Population under study** | **Incidence/**  **10 000** | **95% CI** | |  | **n** | **Population under study** | **Incidence/**  **10 000** | **95% CI** | | |  |
| **Year of pregnancy start date** | | | | | | | | | | | | | | | | | | | | |  |  |
| **2004*** |  | 1076 | 16027 | 671.4 | 633.1 | 711.2 | |  | | 1056 | 14041 | 752.1 | 709 | 796.9 |  | 786 | 10040 | 782.9 | 731.1 | 837.1 | |  |
| **2005** |  | 1473 | 27609 | 533.5 | 507.1 | 560.9 | |  | | 1433 | 19220 | 745.6 | 708.8 | 783.6 |  | 1037 | 13456 | 770.7 | 726.1 | 817 | |  |
| **2006** |  | 1588 | 28221 | 562.7 | 536 | 590.4 | |  | | 1561 | 19706 | 792.1 | 754.8 | 830.7 |  | 1092 | 13527 | 807.3 | 761.9 | 854.5 | |  |
| **2007** |  | 1565 | 29076 | 538.2 | 512.4 | 565 | |  | | 1527 | 20268 | 753.4 | 717.4 | 790.6 |  | 1102 | 13830 | 796.8 | 752.2 | 843.2 | |  |
| **2008** |  | 1618 | 29761 | 543.7 | 518 | 570.3 | |  | | 1569 | 20382 | 769.8 | 733.6 | 807.2 |  | 1043 | 13709 | 760.8 | 717 | 806.5 | |  |
| **2009** |  | 1602 | 29342 | 546 | 520.1 | 572.8 | |  | | 1565 | 20125 | 777.6 | 741 | 815.5 |  | 1048 | 13421 | 780.9 | 736 | 827.6 | |  |
| **2010** |  | 1747 | 27983 | 624.3 | 596.1 | 653.5 | |  | | 1682 | 19479 | 863.5 | 824.4 | 903.8 |  | 1092 | 12763 | 855.6 | 807.6 | 905.5 | |  |
| **2011** |  | 1568 | 27209 | 576.3 | 548.7 | 604.9 | |  | | 1509 | 19073 | 791.2 | 753.2 | 830.4 |  | 1002 | 12349 | 811.4 | 763.8 | 861 | |  |
| **2012** |  | 1237 | 24661 | 501.6 | 474.5 | 529.8 | |  | | 1194 | 16888 | 707 | 668.8 | 746.7 |  | 794 | 10688 | 742.9 | 693.9 | 794.3 | |  |
| **2013** |  | 1007 | 20812 | 483.9 | 454.8 | 514.2 | |  | | 972 | 14269 | 681.2 | 640.4 | 723.8 |  | 621 | 8982 | 691.4 | 639.8 | 745.8 | |  |
| **2014** |  | 773 | 16166 | 478.2 | 445.5 | 512.4 | |  | | 745 | 10959 | 679.8 | 633.4 | 728.6 |  | 502 | 6697 | 749.6 | 687.6 | 815.3 | |  |
| **2015** |  | 521 | 11514 | 452.5 | 414.9 | 492.4 | |  | | 504 | 7903 | 637.7 | 584.8 | 693.9 |  | 334 | 4856 | 687.8 | 618.2 | 762.7 | |  |
| **2016** |  | 367 | 8580 | 427.7 | 385.6 | 473 | |  | | 356 | 5954 | 597.9 | 539 | 661.2 |  | 229 | 3589 | 638.1 | 560.3 | 723.1 | |  |
| **2017°** |  | 6 | 1194 | 50.3 | 18.3 | 109.6 | |  | | *#* | *#* | *#* | *#* | *#* |  | *#* | *#* | *#* | *#* | *#* | |  |
| **Age at pregnancy start date** | | | | | | | | | | | | | | | | | | | | |  |  |
| **18–24** |  | 3192 | 64282 | 496.6 | 479.3 | 514.2 | |  | | 3091 | 40102 | 770.8 | 744.2 | 798 |  | 2047 | 25770 | 794.3 | 760.8 | 828.9 | |  |
| **25–29** |  | 4455 | 73199 | 608.6 | 590.8 | 626.8 | |  | | 4332 | 54429 | 795.9 | 772.9 | 819.3 |  | 2914 | 35746 | 815.2 | 786.6 | 844.5 | |  |
| **30–34** |  | 5240 | 85923 | 609.8 | 593.6 | 626.4 | |  | | 5079 | 65489 | 775.6 | 755 | 796.5 |  | 3563 | 44713 | 796.9 | 771.7 | 822.5 | |  |
| **35–39** |  | 2697 | 56947 | 473.6 | 455.9 | 491.7 | |  | | 2622 | 39366 | 666.1 | 641.6 | 691.2 |  | 1802 | 26076 | 691.1 | 660.5 | 722.5 | |  |
| **40–45** |  | 564 | 17804 | 316.8 | 291 | 344.2 | |  | | 551 | 8942 | 616.2 | 567 | 668.3 |  | 357 | 5627 | 634.4 | 572.1 | 701.3 | |  |
| **Ethnicity** | | | | | | | | | | | | | | | | | | | | |  |  |
| **White** |  | 14234 | 252166 | 564.5 | 555.1 | 573.9 | |  | | 13837 | 178527 | 775.1 | 762.4 | 787.9 |  | 9283 | 115810 | 801.6 | 785.6 | 817.7 | |  |
| **Asian** |  | 779 | 16757 | 464.9 | 432 | 499.5 | |  | | 742 | 12054 | 615.6 | 572.1 | 661.3 |  | 566 | 8918 | 634.7 | 583.6 | 688.8 | |  |
| **Black** |  | 281 | 8821 | 318.6 | 282 | 358.4 | |  | | 269 | 5672 | 474.3 | 420 | 533.4 |  | 194 | 4071 | 476.5 | 412.9 | 546.9 | |  |
| **Mixed** |  | 161 | 3528 | 456.3 | 386 | 535.3 | |  | | 156 | 2320 | 672.4 | 570.4 | 786.3 |  | 103 | 1518 | 678.5 | 552.7 | 822.7 | |  |
| **Other** |  | 311 | 6277 | 495.5 | 440.9 | 554.7 | |  | | 297 | 4360 | 681.2 | 606.5 | 762 |  | 226 | 3342 | 676.2 | 592.1 | 768.3 | |  |
| **Unknown** |  | 382 | 10606 | 360.2 | 325.6 | 397.3 | |  | | 374 | 5395 | 693.2 | 626.4 | 764.8 |  | 311 | 4273 | 727.8 | 651.4 | 810.2 | |  |
| **Index of Multiple Deprivation** | | | | | | | | | | | | | | | | | | | | |  |  |
| **1 (least)** |  | 3550 | 61945 | 573.1 | 554.5 | 592.1 | |  | | 3457 | 44577 | 775.5 | 750.6 | 801 |  | 2427 | 30724 | 789.9 | 759.6 | 821.1 | |  |
| **2** |  | 3355 | 58360 | 574.9 | 555.5 | 594.7 | |  | | 3249 | 41240 | 787.8 | 761.5 | 814.8 |  | 2232 | 27772 | 803.7 | 771.5 | 836.8 | |  |
| **3** |  | 3417 | 58758 | 581.5 | 562.1 | 601.5 | |  | | 3326 | 40881 | 813.6 | 786.7 | 841.1 |  | 2279 | 27328 | 833.9 | 800.7 | 868.1 | |  |
| **4** |  | 3142 | 59246 | 530.3 | 511.6 | 549.6 | |  | | 3029 | 40908 | 740.4 | 714.4 | 767.1 |  | 2047 | 26604 | 769.4 | 736.8 | 803.1 | |  |
| **5 (most)** |  | 2672 | 59612 | 448.2 | 431 | 466 | |  | | 2602 | 40573 | 641.3 | 616.9 | 666.4 |  | 1691 | 25415 | 665.4 | 634.1 | 697.7 | |  |
| **Missing** |  | 12 | 234 | 512.8 | 262.2 | 891.2 | |  | | 12 | 149 | 805.4 | 415.7 | 1380.5 |  | 7 | 89 | 786.5 | 308.2 | 1592.6 | |  |

AP, All Pregnancies; AP24+, All Pregnancies with gestational age ≥24 weeks; LR, Low-Risk pregnancies; n, number of pregnancies in the specified category; CI, confidence interval; #, results where an individual cell count is <5 (to maintain confidentiality).

If a particular pregnancy-related event of interest occurred several times for the same pregnancy, it was only counted once for that pregnancy.

**Table S9.16. Oligohydramnios**

|  |  | **AP cohort** | | | | |  | | **AP24+ cohort** | | | | | |  | **LR cohort** | | | | | |  |
| --- | --- | --- | --- | --- | --- | --- | --- | --- | --- | --- | --- | --- | --- | --- | --- | --- | --- | --- | --- | --- | --- | --- |
|  |  | **n** | **Population under study** | **Incidence/**  **10 000** | **95% CI** | | |  | | **n** | **Population under study** | **Incidence/**  **10 000** | **95% CI** | |  | **n** | **Population under study** | **Incidence/**  **10 000** | **95% CI** | | |  |
| **Year of pregnancy start date** | | | | | | | | | | | | | | | | | | | | |  |  |
| **2004*** |  | 73 | 16027 | 45.5 | 35.7 | 57.2 | |  | | 69 | 14041 | 49.1 | 38.3 | 62.2 |  | 35 | 10040 | 34.9 | 24.3 | 48.5 | |  |
| **2005** |  | 149 | 27609 | 54 | 45.7 | 63.3 | |  | | 137 | 19220 | 71.3 | 59.9 | 84.2 |  | 83 | 13456 | 61.7 | 49.2 | 76.4 | |  |
| **2006** |  | 118 | 28221 | 41.8 | 34.6 | 50.1 | |  | | 116 | 19706 | 58.9 | 48.7 | 70.6 |  | 73 | 13527 | 54 | 42.3 | 67.8 | |  |
| **2007** |  | 143 | 29076 | 49.2 | 41.4 | 58 | |  | | 142 | 20268 | 70.1 | 59.1 | 82.5 |  | 91 | 13830 | 65.8 | 53 | 80.7 | |  |
| **2008** |  | 158 | 29761 | 53.1 | 45.1 | 62.1 | |  | | 145 | 20382 | 71.1 | 60.1 | 83.7 |  | 69 | 13709 | 50.3 | 39.2 | 63.7 | |  |
| **2009** |  | 149 | 29342 | 50.8 | 42.9 | 59.7 | |  | | 141 | 20125 | 70.1 | 59 | 82.6 |  | 82 | 13421 | 61.1 | 48.6 | 75.8 | |  |
| **2010** |  | 157 | 27983 | 56.1 | 47.6 | 65.6 | |  | | 151 | 19479 | 77.5 | 65.7 | 90.9 |  | 81 | 12763 | 63.5 | 50.4 | 78.8 | |  |
| **2011** |  | 142 | 27209 | 52.2 | 43.9 | 61.6 | |  | | 134 | 19073 | 70.3 | 58.9 | 83.1 |  | 69 | 12349 | 55.9 | 43.5 | 70.7 | |  |
| **2012** |  | 132 | 24661 | 53.5 | 44.8 | 63.5 | |  | | 121 | 16888 | 71.6 | 59.5 | 85.5 |  | 76 | 10688 | 71.1 | 56.1 | 88.9 | |  |
| **2013** |  | 123 | 20812 | 59.1 | 49.1 | 70.6 | |  | | 114 | 14269 | 79.9 | 65.9 | 95.9 |  | 58 | 8982 | 64.6 | 49.1 | 83.4 | |  |
| **2014** |  | 100 | 16166 | 61.9 | 50.4 | 75.2 | |  | | 93 | 10959 | 84.9 | 68.6 | 103.8 |  | 50 | 6697 | 74.7 | 55.5 | 98.3 | |  |
| **2015** |  | 50 | 11514 | 43.4 | 32.2 | 57.2 | |  | | 46 | 7903 | 58.2 | 42.7 | 77.5 |  | 26 | 4856 | 53.5 | 35.2 | 78.1 | |  |
| **2016** |  | 55 | 8580 | 64.1 | 48.3 | 83.4 | |  | | 52 | 5954 | 87.3 | 65.3 | 114.4 |  | 33 | 3589 | 91.9 | 63.4 | 128.9 | |  |
| **2017°** |  | *#* | *#* | *#* | *#* | *#* | |  | | *#* | *#* | *#* | *#* | *#* |  | *#* | *#* | *#* | *#* | *#* | |  |
| **Age at pregnancy start date** | | | | | | | | | | | | | | | | | | | | |  |  |
| **18–24** |  | 398 | 64282 | 61.9 | 56 | 68.3 | |  | | 379 | 40102 | 94.5 | 85.2 | 104.5 |  | 212 | 25770 | 82.3 | 71.6 | 94.1 | |  |
| **25–29** |  | 420 | 73199 | 57.4 | 51.9 | 63.2 | |  | | 394 | 54429 | 72.4 | 65.4 | 79.9 |  | 226 | 35746 | 63.2 | 55.2 | 72.1 | |  |
| **30–34** |  | 409 | 85923 | 47.6 | 43 | 52.5 | |  | | 386 | 65489 | 58.9 | 53.2 | 65.1 |  | 226 | 44713 | 50.5 | 44.1 | 57.6 | |  |
| **35–39** |  | 264 | 56947 | 46.4 | 40.9 | 52.3 | |  | | 247 | 39366 | 62.7 | 55.1 | 71.1 |  | 134 | 26076 | 51.4 | 43 | 61 | |  |
| **40–45** |  | 61 | 17804 | 34.3 | 26.3 | 43.9 | |  | | 57 | 8942 | 63.7 | 48.3 | 82.5 |  | 29 | 5627 | 51.5 | 34.4 | 74.2 | |  |
| **Ethnicity** | | | | | | | | | | | | | | | | | | | | |  |  |
| **White** |  | 1303 | 252166 | 51.7 | 48.9 | 54.6 | |  | | 1233 | 178527 | 69.1 | 65.2 | 73.1 |  | 677 | 115810 | 58.5 | 54.1 | 63.1 | |  |
| **Asian** |  | 150 | 16757 | 89.5 | 75.8 | 104.9 | |  | | 142 | 12054 | 117.8 | 99.4 | 138.6 |  | 90 | 8918 | 100.9 | 80.9 | 124.4 | |  |
| **Black** |  | 29 | 8821 | 32.9 | 21.5 | 48.2 | |  | | 25 | 5672 | 44.1 | 28.4 | 65.3 |  | 14 | 4071 | 34.4 | 18.8 | 57.8 | |  |
| **Mixed** |  | 18 | 3528 | 51 | 30.2 | 80.6 | |  | | 15 | 2320 | 64.7 | 36.3 | 106.2 |  | 6 | 1518 | 39.5 | 14.3 | 86.7 | |  |
| **Other** |  | 20 | 6277 | 31.9 | 19.3 | 49.5 | |  | | 16 | 4360 | 36.7 | 20.7 | 60.1 |  | 14 | 3342 | 41.9 | 23 | 70.1 | |  |
| **Unknown** |  | 32 | 10606 | 30.2 | 20.8 | 42.3 | |  | | 32 | 5395 | 59.3 | 40.7 | 83.5 |  | 26 | 4273 | 60.8 | 40.1 | 88.5 | |  |
| **Index of Multiple Deprivation** | | | | | | | | | | | | | | | | | | | | |  |  |
| **1 (least)** |  | 255 | 61945 | 41.2 | 36.2 | 46.6 | |  | | 241 | 44577 | 54.1 | 47.4 | 61.4 |  | 144 | 30724 | 46.9 | 39.5 | 55.2 | |  |
| **2** |  | 265 | 58360 | 45.4 | 40 | 51.3 | |  | | 247 | 41240 | 59.9 | 52.6 | 67.9 |  | 143 | 27772 | 51.5 | 43.3 | 60.7 | |  |
| **3** |  | 291 | 58758 | 49.5 | 43.8 | 55.8 | |  | | 280 | 40881 | 68.5 | 60.5 | 77.3 |  | 167 | 27328 | 61.1 | 52 | 71.4 | |  |
| **4** |  | 356 | 59246 | 60.1 | 54 | 66.7 | |  | | 335 | 40908 | 81.9 | 73.3 | 91.2 |  | 185 | 26604 | 69.5 | 59.8 | 80.4 | |  |
| **5 (most)** |  | 383 | 59612 | 64.2 | 58 | 71 | |  | | 358 | 40573 | 88.2 | 79.4 | 97.8 |  | 187 | 25415 | 73.6 | 63.5 | 84.8 | |  |
| **Missing** |  | *#* | *#* | *#* | *#* | *#* | |  | | *#* | *#* | *#* | *#* | *#* |  | *#* | *#* | *#* | *#* | *#* | |  |

AP, All Pregnancies; AP24+, All Pregnancies with gestational age ≥24 weeks; LR, Low-Risk pregnancies; n, number of pregnancies in the specified category; CI, confidence interval; #, results where an individual cell count is <5 (to maintain confidentiality).

If a particular pregnancy-related event of interest occurred several times for the same pregnancy, it was only counted once for that pregnancy.

**Table S9.17. Polyhydramnios**

|  |  | **AP cohort** | | | | |  | | **AP24+ cohort** | | | | | |  | **LR cohort** | | | | | |  |
| --- | --- | --- | --- | --- | --- | --- | --- | --- | --- | --- | --- | --- | --- | --- | --- | --- | --- | --- | --- | --- | --- | --- |
|  |  | **n** | **Population under study** | **Incidence/**  **10 000** | **95% CI** | | |  | | **n** | **Population under study** | **Incidence/**  **10 000** | **95% CI** | |  | **n** | **Population under study** | **Incidence/**  **10 000** | **95% CI** | | |  |
| **Year of pregnancy start date** | | | | | | | | | | | | | | | | | | | | |  |  |
| **2004*** |  | 61 | 16027 | 38.1 | 29.1 | 48.9 | |  | | 61 | 14041 | 43.4 | 33.2 | 55.8 |  | 32 | 10040 | 31.9 | 21.8 | 45 | |  |
| **2005** |  | 94 | 27609 | 34 | 27.5 | 41.7 | |  | | 94 | 19220 | 48.9 | 39.5 | 59.8 |  | 64 | 13456 | 47.6 | 36.6 | 60.7 | |  |
| **2006** |  | 95 | 28221 | 33.7 | 27.2 | 41.2 | |  | | 93 | 19706 | 47.2 | 38.1 | 57.8 |  | 55 | 13527 | 40.7 | 30.6 | 52.9 | |  |
| **2007** |  | 106 | 29076 | 36.5 | 29.8 | 44.2 | |  | | 102 | 20268 | 50.3 | 41 | 61.1 |  | 63 | 13830 | 45.6 | 35 | 58.2 | |  |
| **2008** |  | 120 | 29761 | 40.3 | 33.4 | 48.2 | |  | | 113 | 20382 | 55.4 | 45.7 | 66.6 |  | 65 | 13709 | 47.4 | 36.6 | 60.4 | |  |
| **2009** |  | 135 | 29342 | 46 | 38.5 | 54.5 | |  | | 133 | 20125 | 66.1 | 55.4 | 78.2 |  | 87 | 13421 | 64.8 | 51.9 | 79.9 | |  |
| **2010** |  | 162 | 27983 | 57.9 | 49.3 | 67.5 | |  | | 158 | 19479 | 81.1 | 69 | 94.7 |  | 91 | 12763 | 71.3 | 57.4 | 87.5 | |  |
| **2011** |  | 174 | 27209 | 63.9 | 54.7 | 74.3 | |  | | 173 | 19073 | 90.7 | 77.7 | 105.2 |  | 87 | 12349 | 70.5 | 56.5 | 86.8 | |  |
| **2012** |  | 186 | 24661 | 75.4 | 65 | 87.1 | |  | | 174 | 16888 | 103 | 88.4 | 119.4 |  | 97 | 10688 | 90.8 | 73.7 | 110.6 | |  |
| **2013** |  | 180 | 20812 | 86.5 | 74.3 | 100.1 | |  | | 171 | 14269 | 119.8 | 102.6 | 139.1 |  | 99 | 8982 | 110.2 | 89.7 | 134 | |  |
| **2014** |  | 148 | 16166 | 91.6 | 77.3 | 107.6 | |  | | 144 | 10959 | 131.4 | 110.9 | 154.5 |  | 72 | 6697 | 107.5 | 84.2 | 135.2 | |  |
| **2015** |  | 104 | 11514 | 90.3 | 73.7 | 109.5 | |  | | 102 | 7903 | 129.1 | 105.3 | 156.5 |  | 58 | 4856 | 119.4 | 90.8 | 154.1 | |  |
| **2016** |  | 98 | 8580 | 114.2 | 92.6 | 139.3 | |  | | 94 | 5954 | 157.9 | 127.8 | 192.9 |  | 52 | 3589 | 144.9 | 108.4 | 189.6 | |  |
| **2017°** |  | 0 | 1194 | 0 |  |  | |  | | 0 | 61 | 0 |  |  |  | 0 | 25 | 0 |  |  | |  |
| **Age at pregnancy start date** | | | | | | | | | | | | | | | | | | | | |  |  |
| **18–24** |  | 253 | 64282 | 39.4 | 34.6 | 44.6 | |  | | 242 | 40102 | 60.3 | 52.9 | 68.6 |  | 124 | 25770 | 48.1 | 39.9 | 57.5 | |  |
| **25–29** |  | 373 | 73199 | 51 | 45.8 | 56.5 | |  | | 360 | 54429 | 66.1 | 59.5 | 73.4 |  | 192 | 35746 | 53.7 | 46.3 | 62 | |  |
| **30–34** |  | 504 | 85923 | 58.7 | 53.6 | 64.1 | |  | | 494 | 65489 | 75.4 | 68.9 | 82.4 |  | 301 | 44713 | 67.3 | 59.9 | 75.4 | |  |
| **35–39** |  | 411 | 56947 | 72.2 | 65.2 | 79.7 | |  | | 398 | 39366 | 101.1 | 91.4 | 111.5 |  | 229 | 26076 | 87.8 | 76.9 | 99.9 | |  |
| **40–45** |  | 122 | 17804 | 68.5 | 56.6 | 82.1 | |  | | 118 | 8942 | 132 | 109.2 | 158 |  | 76 | 5627 | 135.1 | 106.6 | 168.7 | |  |
| **Ethnicity** | | | | | | | | | | | | | | | | | | | | |  |  |
| **White** |  | 1433 | 252166 | 56.8 | 53.9 | 59.9 | |  | | 1393 | 178527 | 78 | 74 | 82.3 |  | 772 | 115810 | 66.7 | 62 | 71.6 | |  |
| **Asian** |  | 80 | 16757 | 47.7 | 37.1 | 60.5 | |  | | 77 | 12054 | 63.9 | 49.9 | 80.6 |  | 47 | 8918 | 52.7 | 38.1 | 71.1 | |  |
| **Black** |  | 68 | 8821 | 77.1 | 59.3 | 98.5 | |  | | 64 | 5672 | 112.8 | 86.6 | 144.4 |  | 46 | 4071 | 113 | 82.7 | 150.6 | |  |
| **Mixed** |  | 21 | 3528 | 59.5 | 36 | 92.6 | |  | | 20 | 2320 | 86.2 | 52.1 | 134.1 |  | 7 | 1518 | 46.1 | 17.1 | 99.4 | |  |
| **Other** |  | 35 | 6277 | 55.8 | 38.2 | 78.6 | |  | | 33 | 4360 | 75.7 | 51.3 | 107.6 |  | 26 | 3342 | 77.8 | 49.7 | 115.9 | |  |
| **Unknown** |  | 26 | 10606 | 24.5 | 16.4 | 35.2 | |  | | 25 | 5395 | 46.3 | 30.1 | 68.1 |  | 24 | 4273 | 56.2 | 36.3 | 83 | |  |
| **Index of Multiple Deprivation** | | | | | | | | | | | | | | | | | | | | |  |  |
| **1 (least)** |  | 310 | 61945 | 50 | 44.5 | 56.1 | |  | | 302 | 44577 | 67.7 | 60.1 | 76 |  | 188 | 30724 | 61.2 | 52.5 | 70.9 | |  |
| **2** |  | 313 | 58360 | 53.6 | 47.7 | 60.1 | |  | | 305 | 41240 | 74 | 65.8 | 82.9 |  | 187 | 27772 | 67.3 | 57.8 | 78 | |  |
| **3** |  | 278 | 58758 | 47.3 | 41.9 | 53.2 | |  | | 273 | 40881 | 66.8 | 59.2 | 75.1 |  | 152 | 27328 | 55.6 | 47.2 | 65.1 | |  |
| **4** |  | 358 | 59246 | 60.4 | 54.3 | 67.1 | |  | | 343 | 40908 | 83.8 | 75.2 | 93.2 |  | 197 | 26604 | 74 | 63.9 | 85.4 | |  |
| **5 (most)** |  | 399 | 59612 | 66.9 | 60.5 | 73.9 | |  | | 384 | 40573 | 94.6 | 85.4 | 104.6 |  | 196 | 25415 | 77.1 | 66.6 | 88.8 | |  |
| **Missing** |  | 5 | 234 | 213.7 | 62.1 | 522.2 | |  | | 5 | 149 | 335.6 | 103.3 | 790.6 |  | *#* | *#* | *#* | *#* | *#* | |  |

AP, All Pregnancies; AP24+, All Pregnancies with gestational age ≥24 weeks; LR, Low-Risk pregnancies; n, number of pregnancies in the specified category; CI, confidence interval; #, results where an individual cell count is <5 (to maintain confidentiality).

If a particular pregnancy-related event of interest occurred several times for the same pregnancy, it was only counted once for that pregnancy.

**Table S9.18. Intrauterine growth restriction/poor fetal growth**

|  |  | **AP cohort** | | | | |  | | **AP24+ cohort** | | | | | |  | **LR cohort** | | | | | |  |
| --- | --- | --- | --- | --- | --- | --- | --- | --- | --- | --- | --- | --- | --- | --- | --- | --- | --- | --- | --- | --- | --- | --- |
|  |  | **n** | **Population under study** | **Incidence/**  **10 000** | **95% CI** | | |  | | **n** | **Population under study** | **Incidence/**  **10 000** | **95% CI** | |  | **n** | **Population under study** | **Incidence/**  **10 000** | **95% CI** | | |  |
| **Year of pregnancy start date** | | | | | | | | | | | | | | | | | | | | |  |  |
| **2004*** |  | 207 | 16027 | 129.2 | 112.3 | 147.9 | |  | | 204 | 14041 | 145.3 | 126.2 | 166.5 |  | 125 | 10040 | 124.5 | 103.7 | 148.2 | |  |
| **2005** |  | 314 | 27609 | 113.7 | 101.4 | 127.1 | |  | | 300 | 19220 | 156.1 | 139 | 174.6 |  | 189 | 13456 | 140.5 | 121.3 | 161.8 | |  |
| **2006** |  | 349 | 28221 | 123.7 | 111 | 137.4 | |  | | 339 | 19706 | 172 | 154.3 | 191.2 |  | 208 | 13527 | 153.8 | 133.7 | 176 | |  |
| **2007** |  | 350 | 29076 | 120.4 | 108.1 | 133.7 | |  | | 340 | 20268 | 167.8 | 150.5 | 186.4 |  | 186 | 13830 | 134.5 | 116 | 155.1 | |  |
| **2008** |  | 398 | 29761 | 133.7 | 120.9 | 147.5 | |  | | 379 | 20382 | 185.9 | 167.8 | 205.4 |  | 209 | 13709 | 152.5 | 132.6 | 174.4 | |  |
| **2009** |  | 356 | 29342 | 121.3 | 109 | 134.7 | |  | | 344 | 20125 | 170.9 | 153.5 | 189.8 |  | 190 | 13421 | 141.6 | 122.3 | 163 | |  |
| **2010** |  | 395 | 27983 | 141.2 | 127.6 | 155.7 | |  | | 381 | 19479 | 195.6 | 176.6 | 216 |  | 220 | 12763 | 172.4 | 150.5 | 196.5 | |  |
| **2011** |  | 412 | 27209 | 151.4 | 137.2 | 166.7 | |  | | 394 | 19073 | 206.6 | 186.9 | 227.8 |  | 208 | 12349 | 168.4 | 146.5 | 192.7 | |  |
| **2012** |  | 392 | 24661 | 159 | 143.6 | 175.5 | |  | | 376 | 16888 | 222.6 | 200.9 | 246 |  | 203 | 10688 | 189.9 | 164.9 | 217.6 | |  |
| **2013** |  | 407 | 20812 | 195.6 | 177 | 215.5 | |  | | 383 | 14269 | 268.4 | 242.5 | 296.2 |  | 213 | 8982 | 237.1 | 206.7 | 270.8 | |  |
| **2014** |  | 327 | 16166 | 202.3 | 181 | 225.4 | |  | | 316 | 10959 | 288.3 | 257.8 | 321.4 |  | 163 | 6697 | 243.4 | 207.8 | 283.2 | |  |
| **2015** |  | 264 | 11514 | 229.3 | 202.5 | 258.6 | |  | | 252 | 7903 | 318.9 | 281.3 | 359.9 |  | 137 | 4856 | 282.1 | 237.5 | 332.5 | |  |
| **2016** |  | 236 | 8580 | 275.1 | 241.3 | 312.1 | |  | | 230 | 5954 | 386.3 | 338.8 | 438.4 |  | 119 | 3589 | 331.6 | 275.4 | 395.5 | |  |
| **2017°** |  | 5 | 1194 | 41.9 | 13.5 | 97.9 | |  | | *#* | *#* | *#* | *#* | *#* |  | 0 | 25 | 0 |  |  | |  |
| **Age at pregnancy start date** | | | | | | | | | | | | | | | | | | | | |  |  |
| **18–24** |  | 1249 | 64282 | 194.3 | 183.7 | 205.4 | |  | | 1198 | 40102 | 298.7 | 282.1 | 316.1 |  | 671 | 25770 | 260.4 | 241 | 280.9 | |  |
| **25–29** |  | 1170 | 73199 | 159.8 | 150.7 | 169.3 | |  | | 1121 | 54429 | 206 | 194.1 | 218.3 |  | 621 | 35746 | 173.7 | 160.4 | 187.8 | |  |
| **30–34** |  | 1127 | 85923 | 131.2 | 123.5 | 139.2 | |  | | 1095 | 65489 | 167.2 | 157.4 | 177.5 |  | 626 | 44713 | 140 | 129.2 | 151.5 | |  |
| **35–39** |  | 678 | 56947 | 119.1 | 110.1 | 128.5 | |  | | 642 | 39366 | 163.1 | 150.7 | 176.2 |  | 354 | 26076 | 135.8 | 122.1 | 150.6 | |  |
| **40–45** |  | 188 | 17804 | 105.6 | 90.8 | 122 | |  | | 184 | 8942 | 205.8 | 177.3 | 237.4 |  | 98 | 5627 | 174.2 | 141.7 | 211.7 | |  |
| **Ethnicity** | | | | | | | | | | | | | | | | | | | | |  |  |
| **White** |  | 3623 | 252166 | 143.7 | 138.9 | 148.5 | |  | | 3490 | 178527 | 195.5 | 189 | 202.2 |  | 1872 | 115810 | 161.6 | 154.3 | 169.2 | |  |
| **Asian** |  | 484 | 16757 | 288.8 | 263.8 | 315.5 | |  | | 464 | 12054 | 384.9 | 351 | 421.1 |  | 313 | 8918 | 351 | 313.7 | 391.4 | |  |
| **Black** |  | 117 | 8821 | 132.6 | 108.5 | 160.4 | |  | | 111 | 5672 | 195.7 | 160.2 | 236.5 |  | 71 | 4071 | 174.4 | 135.2 | 221.3 | |  |
| **Mixed** |  | 46 | 3528 | 130.4 | 94.5 | 175.3 | |  | | 43 | 2320 | 185.3 | 134 | 249.6 |  | 23 | 1518 | 151.5 | 96.4 | 226.4 | |  |
| **Other** |  | 74 | 6277 | 117.9 | 92 | 148.7 | |  | | 66 | 4360 | 151.4 | 116.7 | 193.1 |  | 42 | 3342 | 125.7 | 90 | 170.7 | |  |
| **Unknown** |  | 68 | 10606 | 64.1 | 49.8 | 81.2 | |  | | 66 | 5395 | 122.3 | 94 | 156.5 |  | 49 | 4273 | 114.7 | 84.5 | 152 | |  |
| **Index of Multiple Deprivation** | | | | | | | | | | | | | | | | | | | | |  |  |
| **1 (least)** |  | 607 | 61945 | 98 | 90.1 | 106.4 | |  | | 581 | 44577 | 130.3 | 119.8 | 141.6 |  | 340 | 30724 | 110.7 | 99.1 | 123.1 | |  |
| **2** |  | 677 | 58360 | 116 | 107.3 | 125.3 | |  | | 654 | 41240 | 158.6 | 146.6 | 171.3 |  | 395 | 27772 | 142.2 | 128.4 | 157.2 | |  |
| **3** |  | 720 | 58758 | 122.5 | 113.6 | 132 | |  | | 689 | 40881 | 168.5 | 156.1 | 181.7 |  | 396 | 27328 | 144.9 | 130.9 | 160 | |  |
| **4** |  | 957 | 59246 | 161.5 | 151.3 | 172.3 | |  | | 926 | 40908 | 226.4 | 211.9 | 241.5 |  | 511 | 26604 | 192.1 | 175.7 | 209.6 | |  |
| **5 (most)** |  | 1446 | 59612 | 242.6 | 230 | 255.6 | |  | | 1386 | 40573 | 341.6 | 323.7 | 360.2 |  | 726 | 25415 | 285.7 | 265 | 307.5 | |  |
| **Missing** |  | 5 | 234 | 213.7 | 89 | 427.3 | |  | | *#* | *#* | *#* | *#* | *#* |  | *#* | *#* | *#* | *#* | *#* | |  |

AP, All Pregnancies; AP24+, All Pregnancies with gestational age ≥24 weeks; LR, Low-Risk pregnancies; n, number of pregnancies in the specified category; CI, confidence interval; #, results where an individual cell count is <5 (to maintain confidentiality).

If a particular pregnancy-related event of interest occurred several times for the same pregnancy, it was only counted once for that pregnancy.

**Table S9.19. Gestational diabetes mellitus**

|  |  | **AP cohort** | | | | |  | | **AP24+ cohort** | | | | | |  | **LR cohort** | | | | | |  |
| --- | --- | --- | --- | --- | --- | --- | --- | --- | --- | --- | --- | --- | --- | --- | --- | --- | --- | --- | --- | --- | --- | --- |
|  |  | **n** | **Population under study** | **Incidence/**  **10 000** | **95% CI** | | |  | | **n** | **Population under study** | **Incidence/**  **10 000** | **95% CI** | |  | **n** | **Population under study** | **Incidence/**  **10 000** | **95% CI** | | |  |
| **Year of pregnancy start date** | | | | | | | | | | | | | | | | | | | | |  |  |
| **2004*** |  | 178 | 16027 | 111.1 | 95.4 | 128.5 | |  | | 175 | 14041 | 124.6 | 106.9 | 144.4 |  | 105 | 10040 | 104.6 | 85.6 | 126.5 | |  |
| **2005** |  | 263 | 27609 | 95.3 | 84 | 107.5 | |  | | 255 | 19220 | 132.7 | 117 | 149.9 |  | 156 | 13456 | 115.9 | 98.5 | 135.5 | |  |
| **2006** |  | 324 | 28221 | 114.8 | 102.6 | 128 | |  | | 316 | 19706 | 160.4 | 143.3 | 178.9 |  | 171 | 13527 | 126.4 | 108.3 | 146.7 | |  |
| **2007** |  | 350 | 29076 | 120.4 | 108.1 | 133.6 | |  | | 341 | 20268 | 168.2 | 151 | 186.9 |  | 199 | 13830 | 143.9 | 124.7 | 165.2 | |  |
| **2008** |  | 420 | 29761 | 141.1 | 127.9 | 155.3 | |  | | 412 | 20382 | 202.1 | 183.3 | 222.4 |  | 228 | 13709 | 166.3 | 145.6 | 189.1 | |  |
| **2009** |  | 494 | 29342 | 168.4 | 153.9 | 183.8 | |  | | 482 | 20125 | 239.5 | 218.8 | 261.6 |  | 274 | 13421 | 204.2 | 180.9 | 229.5 | |  |
| **2010** |  | 544 | 27983 | 194.4 | 178.4 | 211.4 | |  | | 529 | 19479 | 271.6 | 249.2 | 295.4 |  | 298 | 12763 | 233.5 | 208 | 261.2 | |  |
| **2011** |  | 559 | 27209 | 205.4 | 188.8 | 223.1 | |  | | 546 | 19073 | 286.3 | 263.1 | 310.9 |  | 307 | 12349 | 248.6 | 221.9 | 277.6 | |  |
| **2012** |  | 546 | 24661 | 221.4 | 203.3 | 240.6 | |  | | 522 | 16888 | 309.1 | 283.5 | 336.3 |  | 299 | 10688 | 279.8 | 249.3 | 312.8 | |  |
| **2013** |  | 550 | 20812 | 264.3 | 242.8 | 287.1 | |  | | 526 | 14269 | 368.6 | 338.3 | 400.9 |  | 292 | 8982 | 325.1 | 289.4 | 363.9 | |  |
| **2014** |  | 453 | 16166 | 280.2 | 255.1 | 307 | |  | | 440 | 10959 | 401.5 | 365.5 | 440 |  | 249 | 6697 | 371.8 | 327.8 | 419.9 | |  |
| **2015** |  | 330 | 11514 | 286.6 | 256.6 | 319.1 | |  | | 324 | 7903 | 410 | 367.3 | 456.1 |  | 184 | 4856 | 378.9 | 327 | 436.5 | |  |
| **2016** |  | 333 | 8580 | 388.1 | 347.7 | 431.7 | |  | | 325 | 5954 | 545.9 | 489.5 | 606.6 |  | 177 | 3589 | 493.2 | 424.6 | 569.2 | |  |
| **2017°** |  | 8 | 1194 | 67 | 28.8 | 132.1 | |  | | 6 | 61 | 983.6 | 365.8 | 2030 |  | *#* | *#* | *#* | *#* | *#* | |  |
| **Age at pregnancy start date** | | | | | | | | | | | | | | | | | | | | |  |  |
| **18–24** |  | 427 | 64282 | 66.4 | 60.1 | 73.2 | |  | | 407 | 40102 | 101.5 | 91.7 | 112.1 |  | 233 | 25770 | 90.4 | 79 | 103 | |  |
| **25–29** |  | 1085 | 73199 | 148.2 | 139.3 | 157.6 | |  | | 1054 | 54429 | 193.6 | 182 | 205.9 |  | 591 | 35746 | 165.3 | 152.1 | 179.4 | |  |
| **30–34** |  | 1805 | 85923 | 210.1 | 200.2 | 220.3 | |  | | 1762 | 65489 | 269.1 | 256.5 | 282 |  | 1028 | 44713 | 229.9 | 215.9 | 244.6 | |  |
| **35–39** |  | 1554 | 56947 | 272.9 | 259.2 | 287.1 | |  | | 1507 | 39366 | 382.8 | 363.8 | 402.6 |  | 849 | 26076 | 325.6 | 304 | 348.3 | |  |
| **40–45** |  | 481 | 17804 | 270.2 | 246.5 | 295.4 | |  | | 469 | 8942 | 524.5 | 478.9 | 573 |  | 240 | 5627 | 426.5 | 374.9 | 482.9 | |  |
| **Ethnicity** | | | | | | | | | | | | | | | | | | | | |  |  |
| **White** |  | 3852 | 252166 | 152.8 | 147.7 | 157.9 | |  | | 3760 | 178527 | 210.6 | 203.7 | 217.7 |  | 2025 | 115810 | 174.9 | 167.1 | 182.9 | |  |
| **Asian** |  | 846 | 16757 | 504.9 | 470.3 | 541.2 | |  | | 820 | 12054 | 680.3 | 634.1 | 728.7 |  | 528 | 8918 | 592.1 | 542.3 | 645 | |  |
| **Black** |  | 262 | 8821 | 297 | 261.5 | 335.8 | |  | | 246 | 5672 | 433.7 | 381.4 | 490.9 |  | 132 | 4071 | 324.2 | 271.2 | 384.3 | |  |
| **Mixed** |  | 78 | 3528 | 221.1 | 171.3 | 280.5 | |  | | 74 | 2320 | 319 | 247.5 | 404 |  | 46 | 1518 | 303 | 217.8 | 409.6 | |  |
| **Other** |  | 199 | 6277 | 317 | 274.1 | 364.6 | |  | | 190 | 4360 | 435.8 | 375.9 | 502.2 |  | 128 | 3342 | 383 | 319.1 | 455.6 | |  |
| **Unknown** |  | 115 | 10606 | 108.4 | 89.7 | 129.9 | |  | | 109 | 5395 | 202 | 165.3 | 244.3 |  | 82 | 4273 | 191.9 | 151.3 | 239.8 | |  |
| **Index of Multiple Deprivation** | | | | | | | | | | | | | | | | | | | | |  |  |
| **1 (least)** |  | 972 | 61945 | 156.9 | 146.8 | 167.5 | |  | | 950 | 44577 | 213.1 | 199.4 | 227.5 |  | 551 | 30724 | 179.3 | 164.2 | 195.5 | |  |
| **2** |  | 984 | 58360 | 168.6 | 157.7 | 180.1 | |  | | 960 | 41240 | 232.8 | 217.8 | 248.5 |  | 568 | 27772 | 204.5 | 187.5 | 222.7 | |  |
| **3** |  | 1060 | 58758 | 180.4 | 169.2 | 192.1 | |  | | 1030 | 40881 | 252 | 236.3 | 268.3 |  | 594 | 27328 | 217.4 | 199.7 | 236.1 | |  |
| **4** |  | 1101 | 59246 | 185.8 | 174.5 | 197.7 | |  | | 1067 | 40908 | 260.8 | 245 | 277.4 |  | 597 | 26604 | 224.4 | 206.4 | 243.6 | |  |
| **5 (most)** |  | 1228 | 59612 | 206 | 194 | 218.5 | |  | | 1185 | 40573 | 292.1 | 275.1 | 309.8 |  | 626 | 25415 | 246.3 | 226.7 | 267.1 | |  |
| **Missing** |  | 7 | 234 | 299.1 | 112.2 | 634.4 | |  | | 7 | 149 | 469.8 | 187.8 | 953.2 |  | 5 | 89 | 561.8 | 178.8 | 1284.4 | |  |

AP, All Pregnancies; AP24+, All Pregnancies with gestational age ≥24 weeks; LR, Low-Risk pregnancies; n, number of pregnancies in the specified category; CI, confidence interval; #, results where an individual cell count is <5 (to maintain confidentiality).

If a particular pregnancy-related event of interest occurred several times for the same pregnancy, it was only counted once for that pregnancy.

**Table S9.20. Maternal death (any time)**

|  |  | **AP cohort** | | | | |  | | **AP24+ cohort** | | | | | |  | **LR cohort** | | | | | |  |
| --- | --- | --- | --- | --- | --- | --- | --- | --- | --- | --- | --- | --- | --- | --- | --- | --- | --- | --- | --- | --- | --- | --- |
|  |  | **n** | **Population under study** | **Incidence/**  **10 000** | **95% CI** | | |  | | **n** | **Population under study** | **Incidence/**  **10 000** | **95% CI** | |  | **n** | **Population under study** | **Incidence/**  **10 000** | **95% CI** | | |  |
| **Year of pregnancy start date** | | | | | | | | | | | | | | | | | | | | |  |  |
| **2004*** |  | *#* | *#* | *#* | *#* | *#* | |  | | *#* | *#* | *#* | *#* | *#* |  | *#* | *#* | *#* | *#* | *#* | |  |
| **2005** |  | *#* | *#* | *#* | *#* | *#* | |  | | *#* | *#* | *#* | *#* | *#* |  | *#* | *#* | *#* | *#* | *#* | |  |
| **2006** |  | *#* | *#* | *#* | *#* | *#* | |  | | *#* | *#* | *#* | *#* | *#* |  | *#* | *#* | *#* | *#* | *#* | |  |
| **2007** |  | *#* | *#* | *#* | *#* | *#* | |  | | *#* | *#* | *#* | *#* | *#* |  | *#* | *#* | *#* | *#* | *#* | |  |
| **2008** |  | *#* | *#* | *#* | *#* | *#* | |  | | *#* | *#* | *#* | *#* | *#* |  | *#* | *#* | *#* | *#* | *#* | |  |
| **2009** |  | *#* | *#* | *#* | *#* | *#* | |  | | *#* | *#* | *#* | *#* | *#* |  | *#* | *#* | *#* | *#* | *#* | |  |
| **2010** |  | *#* | *#* | *#* | *#* | *#* | |  | | *#* | *#* | *#* | *#* | *#* |  | *#* | *#* | *#* | *#* | *#* | |  |
| **2011** |  | *#* | *#* | *#* | *#* | *#* | |  | | *#* | *#* | *#* | *#* | *#* |  | *#* | *#* | *#* | *#* | *#* | |  |
| **2012** |  | *#* | *#* | *#* | *#* | *#* | |  | | *#* | *#* | *#* | *#* | *#* |  | *#* | *#* | *#* | *#* | *#* | |  |
| **2013** |  | *#* | *#* | *#* | *#* | *#* | |  | | *#* | *#* | *#* | *#* | *#* |  | *#* | *#* | *#* | *#* | *#* | |  |
| **2014** |  | *#* | *#* | *#* | *#* | *#* | |  | | *#* | *#* | *#* | *#* | *#* |  | *#* | *#* | *#* | *#* | *#* | |  |
| **2015** |  | *#* | *#* | *#* | *#* | *#* | |  | | *#* | *#* | *#* | *#* | *#* |  | *#* | *#* | *#* | *#* | *#* | |  |
| **2016** |  | *#* | *#* | *#* | *#* | *#* | |  | | *#* | *#* | *#* | *#* | *#* |  | *#* | *#* | *#* | *#* | *#* | |  |
| **2017°** |  | *#* | *#* | *#* | *#* | *#* | |  | | *#* | *#* | *#* | *#* | *#* |  | *#* | *#* | *#* | *#* | *#* | |  |
| **Age at pregnancy start date** | | | | | | | | | | | | | | | | | | | | |  |  |
| **18–24** |  | *#* | *#* | *#* | *#* | *#* | |  | | *#* | *#* | *#* | *#* | *#* |  | *#* | *#* | *#* | *#* | *#* | |  |
| **25–29** |  | *#* | *#* | *#* | *#* | *#* | |  | | *#* | *#* | *#* | *#* | *#* |  | *#* | *#* | *#* | *#* | *#* | |  |
| **30–34** |  | 5 | 85923 | 0.6 | 0.2 | 1.4 | |  | | *#* | *#* | *#* | *#* | *#* |  | *#* | *#* | *#* | *#* | *#* | |  |
| **35–39** |  | 7 | 56947 | 1.2 | 0.5 | 2.6 | |  | | *#* | *#* | *#* | *#* | *#* |  | *#* | *#* | *#* | *#* | *#* | |  |
| **40–45** |  | *#* | *#* | *#* | *#* | *#* | |  | | *#* | *#* | *#* | *#* | *#* |  | *#* | *#* | *#* | *#* | *#* | |  |
| **Ethnicity** | | | | | | | | | | | | | | | | | | | | |  |  |
| **White** |  | 12 | 252166 | 0.5 | 0.2 | 0.9 | |  | | 10 | 178527 | 0.6 | 0.2 | 1.1 |  | 8 | 115810 | 0.7 | 0.3 | 1.4 | |  |
| **Asian** |  | *#* | *#* | *#* | *#* | *#* | |  | | *#* | *#* | *#* | *#* | *#* |  | *#* | *#* | *#* | *#* | *#* | |  |
| **Black** |  | *#* | *#* | *#* | *#* | *#* | |  | | 0 | 5672 | 0 |  |  |  | 0 | 4071 | 0 |  |  | |  |
| **Mixed** |  | 0 | 3528 | 0 |  |  | |  | | 0 | 2320 | 0 |  |  |  | 0 | 1518 | 0 |  |  | |  |
| **Other** |  | *#* | *#* | *#* | *#* | *#* | |  | | *#* | *#* | *#* | *#* | *#* |  | *#* | *#* | *#* | *#* | *#* | |  |
| **Unknown** |  | *#* | *#* | *#* | *#* | *#* | |  | | 0 | 5395 | 0 |  |  |  | 0 | 4273 | 0 |  |  | |  |
| **Index of Multiple Deprivation** | | | | | | | | | | | | | | | | | | | | |  |  |
| **1 (least)** |  | *#* | *#* | *#* | *#* | *#* | |  | | *#* | *#* | *#* | *#* | *#* |  | *#* | *#* | *#* | *#* | *#* | |  |
| **2** |  | 6 | 58360 | 1 | 0.3 | 2.6 | |  | | *#* | *#* | *#* | *#* | *#* |  | *#* | *#* | *#* | *#* | *#* | |  |
| **3** |  | *#* | *#* | *#* | *#* | *#* | |  | | *#* | *#* | *#* | *#* | *#* |  | *#* | *#* | *#* | *#* | *#* | |  |
| **4** |  | 5 | 59246 | 0.8 | 0.2 | 2.1 | |  | | *#* | *#* | *#* | *#* | *#* |  | *#* | *#* | *#* | *#* | *#* | |  |
| **5 (most)** |  | 5 | 59612 | 0.8 | 0.3 | 2 | |  | | *#* | *#* | *#* | *#* | *#* |  | *#* | *#* | *#* | *#* | *#* | |  |
| **Missing** |  | 0 | 234 | 0 |  |  | |  | | 0 | 149 | 0 |  |  |  | 0 | 89 | 0 |  |  | |  |

AP, All Pregnancies; AP24+, All Pregnancies with gestational age ≥24 weeks; LR, Low-Risk pregnancies; n, number of pregnancies in the specified category; CI, confidence interval; #, results where an individual cell count is <5 (to maintain confidentiality).

**Table S9.21. Maternal death (before delivery)**

|  |  | **AP cohort** | | | | |  | | **AP24+ cohort** | | | | | |  | **LR cohort** | | | | | |  |
| --- | --- | --- | --- | --- | --- | --- | --- | --- | --- | --- | --- | --- | --- | --- | --- | --- | --- | --- | --- | --- | --- | --- |
|  |  | **n** | **Population under study** | **Incidence/**  **10 000** | **95% CI** | | |  | | **n** | **Population under study** | **Incidence/**  **10 000** | **95% CI** | |  | **n** | **Population under study** | **Incidence/**  **10 000** | **95% CI** | | |  |
| **Year of pregnancy start date** | | | | | | | | | | | | | | | | | | | | |  |  |
| **2004*** |  | 0 | 16027 | 0 |  |  | |  | | 0 | 14041 | 0 |  |  |  | 0 | 10040 | 0 |  |  | |  |
| **2005** |  | 0 | 27609 | 0 |  |  | |  | | 0 | 19220 | 0 |  |  |  | 0 | 13456 | 0 |  |  | |  |
| **2006** |  | 0 | 28221 | 0 |  |  | |  | | 0 | 19706 | 0 |  |  |  | 0 | 13527 | 0 |  |  | |  |
| **2007** |  | 0 | 29076 | 0 |  |  | |  | | 0 | 20268 | 0 |  |  |  | 0 | 13830 | 0 |  |  | |  |
| **2008** |  | *#* | *#* | *#* | *#* | *#* | |  | | 0 | 20382 | 0 |  |  |  | 0 | 13709 | 0 |  |  | |  |
| **2009** |  | 0 | 29342 | 0 |  |  | |  | | 0 | 20125 | 0 |  |  |  | 0 | 13421 | 0 |  |  | |  |
| **2010** |  | *#* | *#* | *#* | *#* | *#* | |  | | *#* | *#* | *#* | *#* | *#* |  | *#* | *#* | *#* | *#* | *#* | |  |
| **2011** |  | 0 | 27209 | 0 |  |  | |  | | 0 | 19073 | 0 |  |  |  | 0 | 12349 | 0 |  |  | |  |
| **2012** |  | 0 | 24661 | 0 |  |  | |  | | 0 | 16888 | 0 |  |  |  | 0 | 10688 | 0 |  |  | |  |
| **2013** |  | 0 | 20812 | 0 |  |  | |  | | 0 | 14269 | 0 |  |  |  | 0 | 8982 | 0 |  |  | |  |
| **2014** |  | 0 | 16166 | 0 |  |  | |  | | 0 | 10959 | 0 |  |  |  | 0 | 6697 | 0 |  |  | |  |
| **2015** |  | *#* | *#* | *#* | *#* | *#* | |  | | *#* | *#* | *#* | *#* | *#* |  | 0 | 4856 | 0 |  |  | |  |
| **2016** |  | 0 | 8580 | 0 |  |  | |  | | 0 | 5954 | 0 |  |  |  | 0 | 3589 | 0 |  |  | |  |
| **2017°** |  | 0 | 1194 | 0 |  |  | |  | | 0 | 61 | 0 |  |  |  | 0 | 25 | 0 |  |  | |  |
| **Age at pregnancy start date** | | | | | | | | | | | | | | | | | | | | |  |  |
| **18–24** |  | *#* | *#* | *#* | *#* | *#* | |  | | *#* | *#* | *#* | *#* | *#* |  | *#* | *#* | *#* | *#* | *#* | |  |
| **25–29** |  | 0 | 73199 | 0 |  |  | |  | | 0 | 54429 | 0 |  |  |  | 0 | 35746 | 0 |  |  | |  |
| **30–34** |  | *#* | *#* | *#* | *#* | *#* | |  | | *#* | *#* | *#* | *#* | *#* |  | 0 | 44713 | 0 |  |  | |  |
| **35–39** |  | 0 | 56947 | 0 |  |  | |  | | 0 | 39366 | 0 |  |  |  | 0 | 26076 | 0 |  |  | |  |
| **40–45** |  | 0 | 17804 | 0 |  |  | |  | | 0 | 8942 | 0 |  |  |  | 0 | 5627 | 0 |  |  | |  |
| **Ethnicity** | | | | | | | | | | | | | | | | | | | | |  |  |
| **White** |  | *#* | *#* | *#* | *#* | *#* | |  | | *#* | *#* | *#* | *#* | *#* |  | 0 | 115810 | 0 |  |  | |  |
| **Asian** |  | *#* | *#* | *#* | *#* | *#* | |  | | *#* | *#* | *#* | *#* | *#* |  | *#* | *#* | *#* | *#* | *#* | |  |
| **Black** |  | 0 | 8821 | 0 |  |  | |  | | 0 | 5672 | 0 |  |  |  | 0 | 4071 | 0 |  |  | |  |
| **Mixed** |  | 0 | 3528 | 0 |  |  | |  | | 0 | 2320 | 0 |  |  |  | 0 | 1518 | 0 |  |  | |  |
| **Other** |  | 0 | 6277 | 0 |  |  | |  | | 0 | 4360 | 0 |  |  |  | 0 | 3342 | 0 |  |  | |  |
| **Unknown** |  | *#* | *#* | *#* | *#* | *#* | |  | | 0 | 5395 | 0 |  |  |  | 0 | 4273 | 0 |  |  | |  |
| **Index of Multiple Deprivation** | | | | | | | | | | | | | | | | | | | | |  |  |
| **1 (least)** |  | 0 | 61945 | 0 |  |  | |  | | 0 | 44577 | 0 |  |  |  | 0 | 30724 | 0 |  |  | |  |
| **2** |  | *#* | *#* | *#* | *#* | *#* | |  | | *#* | *#* | *#* | *#* | *#* |  | 0 | 27772 | 0 |  |  | |  |
| **3** |  | 0 | 58758 | 0 |  |  | |  | | 0 | 40881 | 0 |  |  |  | 0 | 27328 | 0 |  |  | |  |
| **4** |  | 0 | 59246 | 0 |  |  | |  | | 0 | 40908 | 0 |  |  |  | 0 | 26604 | 0 |  |  | |  |
| **5 (most)** |  | *#* | *#* | *#* | *#* | *#* | |  | | *#* | *#* | *#* | *#* | *#* |  | *#* | *#* | *#* | *#* | *#* | |  |
| **Missing** |  | 0 | 234 | 0 |  |  | |  | | 0 | 149 | 0 |  |  |  | 0 | 89 | 0 |  |  | |  |

AP, All Pregnancies; AP24+, All Pregnancies with gestational age ≥24 weeks; LR, Low-Risk pregnancies; n, number of pregnancies in the specified category; CI, confidence interval; #, results where an individual cell count is <5 (to maintain confidentiality).

**Table S9.22. Maternal death (on delivery date or 1 day after)**

|  |  | **AP cohort** | | | | |  | | **AP24+ cohort** | | | | | |  | **LR cohort** | | | | | |  |
| --- | --- | --- | --- | --- | --- | --- | --- | --- | --- | --- | --- | --- | --- | --- | --- | --- | --- | --- | --- | --- | --- | --- |
|  |  | **n** | **Population under study** | **Incidence/**  **10 000** | **95% CI** | | |  | | **n** | **Population under study** | **Incidence/**  **10 000** | **95% CI** | |  | **n** | **Population under study** | **Incidence/**  **10 000** | **95% CI** | | |  |
| **Year of pregnancy start date** | | | | | | | | | | | | | | | | | | | | |  |  |
| **2004*** |  | 0 | 16027 | 0 |  |  | |  | | 0 | 14041 | 0 |  |  |  | 0 | 10040 | 0 |  |  | |  |
| **2005** |  | 0 | 27609 | 0 |  |  | |  | | 0 | 19220 | 0 |  |  |  | 0 | 13456 | 0 |  |  | |  |
| **2006** |  | 0 | 28221 | 0 |  |  | |  | | 0 | 19706 | 0 |  |  |  | 0 | 13527 | 0 |  |  | |  |
| **2007** |  | 0 | 29076 | 0 |  |  | |  | | 0 | 20268 | 0 |  |  |  | 0 | 13830 | 0 |  |  | |  |
| **2008** |  | *#* | *#* | *#* | *#* | *#* | |  | | *#* | *#* | *#* | *#* | *#* |  | *#* | *#* | *#* | *#* | *#* | |  |
| **2009** |  | *#* | *#* | *#* | *#* | *#* | |  | | *#* | *#* | *#* | *#* | *#* |  | *#* | *#* | *#* | *#* | *#* | |  |
| **2010** |  | 0 | 27983 | 0 |  |  | |  | | 0 | 19479 | 0 |  |  |  | 0 | 12763 | 0 |  |  | |  |
| **2011** |  | *#* | *#* | *#* | *#* | *#* | |  | | *#* | *#* | *#* | *#* | *#* |  | *#* | *#* | *#* | *#* | *#* | |  |
| **2012** |  | 0 | 24661 | 0 |  |  | |  | | 0 | 16888 | 0 |  |  |  | 0 | 10688 | 0 |  |  | |  |
| **2013** |  | 0 | 20812 | 0 |  |  | |  | | 0 | 14269 | 0 |  |  |  | 0 | 8982 | 0 |  |  | |  |
| **2014** |  | *#* | *#* | *#* | *#* | *#* | |  | | *#* | *#* | *#* | *#* | *#* |  | *#* | *#* | *#* | *#* | *#* | |  |
| **2015** |  | 0 | 11514 | 0 |  |  | |  | | 0 | 7903 | 0 |  |  |  | 0 | 4856 | 0 |  |  | |  |
| **2016** |  | 0 | 8580 | 0 |  |  | |  | | 0 | 5954 | 0 |  |  |  | 0 | 3589 | 0 |  |  | |  |
| **2017°** |  | *#* | *#* | *#* | *#* | *#* | |  | | *#* | *#* | *#* | *#* | *#* |  | *#* | *#* | *#* | *#* | *#* | |  |
| **Age at pregnancy start date** | | | | | | | | | | | | | | | | | | | | |  |  |
| **18–24** |  | *#* | *#* | *#* | *#* | *#* | |  | | *#* | *#* | *#* | *#* | *#* |  | *#* | *#* | *#* | *#* | *#* | |  |
| **25–29** |  | *#* | *#* | *#* | *#* | *#* | |  | | *#* | *#* | *#* | *#* | *#* |  | *#* | *#* | *#* | *#* | *#* | |  |
| **30–34** |  | *#* | *#* | *#* | *#* | *#* | |  | | *#* | *#* | *#* | *#* | *#* |  | *#* | *#* | *#* | *#* | *#* | |  |
| **35–39** |  | *#* | *#* | *#* | *#* | *#* | |  | | *#* | *#* | *#* | *#* | *#* |  | *#* | *#* | *#* | *#* | *#* | |  |
| **40–45** |  | 0 | 17804 | 0 |  |  | |  | | 0 | 8942 | 0 |  |  |  | 0 | 5627 | 0 |  |  | |  |
| **Ethnicity** | | | | | | | | | | | | | | | | | | | | |  |  |
| **White** |  | *#* | *#* | *#* | *#* | *#* | |  | | *#* | *#* | *#* | *#* | *#* |  | *#* | *#* | *#* | *#* | *#* | |  |
| **Asian** |  | *#* | *#* | *#* | *#* | *#* | |  | | *#* | *#* | *#* | *#* | *#* |  | *#* | *#* | *#* | *#* | *#* | |  |
| **Black** |  | 0 | 8821 | 0 |  |  | |  | | 0 | 5672 | 0 |  |  |  | 0 | 4071 | 0 |  |  | |  |
| **Mixed** |  | 0 | 3528 | 0 |  |  | |  | | 0 | 2320 | 0 |  |  |  | 0 | 1518 | 0 |  |  | |  |
| **Other** |  | 0 | 6277 | 0 |  |  | |  | | 0 | 4360 | 0 |  |  |  | 0 | 3342 | 0 |  |  | |  |
| **Unknown** |  | 0 | 10606 | 0 |  |  | |  | | 0 | 5395 | 0 |  |  |  | 0 | 4273 | 0 |  |  | |  |
| **Index of Multiple Deprivation** | | | | | | | | | | | | | | | | | | | | |  |  |
| **1 (least)** |  | 0 | 61945 | 0 |  |  | |  | | 0 | 44577 | 0 |  |  |  | 0 | 30724 | 0 |  |  | |  |
| **2** |  | *#* | *#* | *#* | *#* | *#* | |  | | *#* | *#* | *#* | *#* | *#* |  | *#* | *#* | *#* | *#* | *#* | |  |
| **3** |  | *#* | *#* | *#* | *#* | *#* | |  | | *#* | *#* | *#* | *#* | *#* |  | *#* | *#* | *#* | *#* | *#* | |  |
| **4** |  | *#* | *#* | *#* | *#* | *#* | |  | | *#* | *#* | *#* | *#* | *#* |  | *#* | *#* | *#* | *#* | *#* | |  |
| **5 (most)** |  | *#* | *#* | *#* | *#* | *#* | |  | | *#* | *#* | *#* | *#* | *#* |  | *#* | *#* | *#* | *#* | *#* | |  |
| **Missing** |  | 0 | 234 | 0 |  |  | |  | | 0 | 149 | 0 |  |  |  | 0 | 89 | 0 |  |  | |  |

AP, All Pregnancies; AP24+, All Pregnancies with gestational age ≥24 weeks; LR, Low-Risk pregnancies; n, number of pregnancies in the specified category; CI, confidence interval; #, results where an individual cell count is <5 (to maintain confidentiality).

**Table S9.23. Maternal death (2 to 42 days after delivery)**

|  |  | **AP cohort** | | | | |  | | **AP24+ cohort** | | | | | |  | **LR cohort** | | | | | |  |
| --- | --- | --- | --- | --- | --- | --- | --- | --- | --- | --- | --- | --- | --- | --- | --- | --- | --- | --- | --- | --- | --- | --- |
|  |  | **n** | **Population under study** | **Incidence/**  **10 000** | **95% CI** | | |  | | **n** | **Population under study** | **Incidence/**  **10 000** | **95% CI** | |  | **n** | **Population under study** | **Incidence/**  **10 000** | **95% CI** | | |  |
| **Year of pregnancy start date** | | | | | | | | | | | | | | | | | | | | |  |  |
| **2004*** |  | *#* | *#* | *#* | *#* | *#* | |  | | *#* | *#* | *#* | *#* | *#* |  | *#* | *#* | *#* | *#* | *#* | |  |
| **2005** |  | *#* | *#* | *#* | *#* | *#* | |  | | *#* | *#* | *#* | *#* | *#* |  | *#* | *#* | *#* | *#* | *#* | |  |
| **2006** |  | *#* | *#* | *#* | *#* | *#* | |  | | *#* | *#* | *#* | *#* | *#* |  | *#* | *#* | *#* | *#* | *#* | |  |
| **2007** |  | 0 | 29076 | 0 |  |  | |  | | 0 | 20268 | 0 |  |  |  | 0 | 13830 | 0 |  |  | |  |
| **2008** |  | *#* | *#* | *#* | *#* | *#* | |  | | *#* | *#* | *#* | *#* | *#* |  | 0 | 13709 | 0 |  |  | |  |
| **2009** |  | *#* | *#* | *#* | *#* | *#* | |  | | 0 | 20125 | 0 |  |  |  | 0 | 13421 | 0 |  |  | |  |
| **2010** |  | 0 | 27983 | 0 |  |  | |  | | 0 | 19479 | 0 |  |  |  | 0 | 12763 | 0 |  |  | |  |
| **2011** |  | 0 | 27209 | 0 |  |  | |  | | 0 | 19073 | 0 |  |  |  | 0 | 12349 | 0 |  |  | |  |
| **2012** |  | 0 | 24661 | 0 |  |  | |  | | 0 | 16888 | 0 |  |  |  | 0 | 10688 | 0 |  |  | |  |
| **2013** |  | *#* | *#* | *#* | *#* | *#* | |  | | *#* | *#* | *#* | *#* | *#* |  | *#* | *#* | *#* | *#* | *#* | |  |
| **2014** |  | 0 | 16166 | 0 |  |  | |  | | 0 | 10959 | 0 |  |  |  | 0 | 6697 | 0 |  |  | |  |
| **2015** |  | 0 | 11514 | 0 |  |  | |  | | 0 | 7903 | 0 |  |  |  | 0 | 4856 | 0 |  |  | |  |
| **2016** |  | *#* | *#* | *#* | *#* | *#* | |  | | *#* | *#* | *#* | *#* | *#* |  | *#* | *#* | *#* | *#* | *#* | |  |
| **2017°** |  | 0 | 1194 | 0 |  |  | |  | | 0 | 61 | 0 |  |  |  | 0 | 25 | 0 |  |  | |  |
| **Age at pregnancy start date** | | | | | | | | | | | | | | | | | | | | |  |  |
| **18–24** |  | 0 | 64282 | 0 |  |  | |  | | 0 | 40102 | 0 |  |  |  | 0 | 25770 | 0 |  |  | |  |
| **25–29** |  | *#* | *#* | *#* | *#* | *#* | |  | | *#* | *#* | *#* | *#* | *#* |  | *#* | *#* | *#* | *#* | *#* | |  |
| **30–34** |  | *#* | *#* | *#* | *#* | *#* | |  | | *#* | *#* | *#* | *#* | *#* |  | *#* | *#* | *#* | *#* | *#* | |  |
| **35–39** |  | 5 | 56947 | 0.9 | 0.3 | 2.1 | |  | | *#* | *#* | *#* | *#* | *#* |  | *#* | *#* | *#* | *#* | *#* | |  |
| **40–45** |  | *#* | *#* | *#* | *#* | *#* | |  | | *#* | *#* | *#* | *#* | *#* |  | *#* | *#* | *#* | *#* | *#* | |  |
| **Ethnicity** | | | | | | | | | | | | | | | | | | | | |  |  |
| **White** |  | 7 | 252166 | 0.3 | 0.1 | 0.6 | |  | | 5 | 178527 | 0.3 | 0.08 | 0.7 |  | *#* | *#* | *#* | *#* | *#* | |  |
| **Asian** |  | 0 | 16757 | 0 |  |  | |  | | 0 | 12054 | 0 |  |  |  | 0 | 8918 | 0 |  |  | |  |
| **Black** |  | *#* | *#* | *#* | *#* | *#* | |  | | 0 | 5672 | 0 |  |  |  | 0 | 4071 | 0 |  |  | |  |
| **Mixed** |  | 0 | 3528 | 0 |  |  | |  | | 0 | 2320 | 0 |  |  |  | 0 | 1518 | 0 |  |  | |  |
| **Other** |  | *#* | *#* | *#* | *#* | *#* | |  | | *#* | *#* | *#* | *#* | *#* |  | *#* | *#* | *#* | *#* | *#* | |  |
| **Unknown** |  | *#* | *#* | *#* | *#* | *#* | |  | | 0 | 5395 | 0 |  |  |  | 0 | 4273 | 0 |  |  | |  |
| **Index of Multiple Deprivation** | | | | | | | | | | | | | | | | | | | | |  |  |
| **1 (least)** |  | *#* | *#* | *#* | *#* | *#* | |  | | *#* | *#* | *#* | *#* | *#* |  | *#* | *#* | *#* | *#* | *#* | |  |
| **2** |  | *#* | *#* | *#* | *#* | *#* | |  | | *#* | *#* | *#* | *#* | *#* |  | *#* | *#* | *#* | *#* | *#* | |  |
| **3** |  | *#* | *#* | *#* | *#* | *#* | |  | | *#* | *#* | *#* | *#* | *#* |  | *#* | *#* | *#* | *#* | *#* | |  |
| **4** |  | *#* | *#* | *#* | *#* | *#* | |  | | *#* | *#* | *#* | *#* | *#* |  | *#* | *#* | *#* | *#* | *#* | |  |
| **5 (most)** |  | *#* | *#* | *#* | *#* | *#* | |  | | *#* | *#* | *#* | *#* | *#* |  | 0 | 25415 | 0 |  |  | |  |
| **Missing** |  | 0 | 234 | 0 |  |  | |  | | 0 | 149 | 0 |  |  |  | 0 | 89 | 0 |  |  | |  |

AP, All Pregnancies; AP24+, All Pregnancies with gestational age ≥24 weeks; LR, Low-Risk pregnancies; n, number of pregnancies in the specified category; CI, confidence interval; #, results where an individual cell count is <5 (to maintain confidentiality).

**Table S9.24. Preterm premature rupture of membranes**

|  |  | **AP cohort** | | | | |  | | **AP24+ cohort** | | | | | |  | **LR cohort** | | | | | |  |
| --- | --- | --- | --- | --- | --- | --- | --- | --- | --- | --- | --- | --- | --- | --- | --- | --- | --- | --- | --- | --- | --- | --- |
|  |  | **n** | **Population under study** | **Incidence/**  **10 000** | **95% CI** | | |  | | **n** | **Population under study** | **Incidence/**  **10 000** | **95% CI** | |  | **n** | **Population under study** | **Incidence/**  **10 000** | **95% CI** | | |  |
| **Year of pregnancy start date** | | | | | | | | | | | | | | | | | | | | |  |  |
| **2004*** |  | 167 | 16027 | 104.2 | 89.1 | 121.2 | |  | | 159 | 14041 | 113.2 | 96.4 | 132.1 |  | 109 | 10040 | 108.6 | 89.2 | 130.8 | |  |
| **2005** |  | 237 | 27609 | 85.8 | 75.3 | 97.5 | |  | | 212 | 19220 | 110.3 | 96 | 126.1 |  | 138 | 13456 | 102.6 | 86.3 | 121 | |  |
| **2006** |  | 279 | 28221 | 98.9 | 87.6 | 111.2 | |  | | 258 | 19706 | 130.9 | 115.5 | 147.8 |  | 164 | 13527 | 121.2 | 103.5 | 141.1 | |  |
| **2007** |  | 305 | 29076 | 104.9 | 93.5 | 117.3 | |  | | 280 | 20268 | 138.1 | 122.6 | 155.1 |  | 177 | 13830 | 128 | 109.9 | 148.1 | |  |
| **2008** |  | 307 | 29761 | 103.2 | 92 | 115.3 | |  | | 265 | 20382 | 130 | 114.9 | 146.5 |  | 160 | 13709 | 116.7 | 99.4 | 136.1 | |  |
| **2009** |  | 317 | 29342 | 108 | 96.5 | 120.6 | |  | | 278 | 20125 | 138.1 | 122.5 | 155.2 |  | 166 | 13421 | 123.7 | 105.7 | 143.9 | |  |
| **2010** |  | 340 | 27983 | 121.5 | 108.9 | 135.1 | |  | | 286 | 19479 | 146.8 | 130.4 | 164.7 |  | 167 | 12763 | 130.8 | 111.9 | 152.1 | |  |
| **2011** |  | 304 | 27209 | 111.7 | 99.5 | 125.1 | |  | | 245 | 19073 | 128.5 | 113 | 145.5 |  | 143 | 12349 | 115.8 | 97.7 | 136.3 | |  |
| **2012** |  | 343 | 24661 | 139.1 | 124.8 | 154.6 | |  | | 290 | 16888 | 171.7 | 152.7 | 192.4 |  | 162 | 10688 | 151.6 | 129.3 | 176.5 | |  |
| **2013** |  | 271 | 20812 | 130.2 | 115.3 | 146.5 | |  | | 228 | 14269 | 159.8 | 139.8 | 181.7 |  | 133 | 8982 | 148.1 | 124.1 | 175.2 | |  |
| **2014** |  | 211 | 16166 | 130.5 | 113.5 | 149.3 | |  | | 180 | 10959 | 164.2 | 141.3 | 189.8 |  | 89 | 6697 | 132.9 | 106.9 | 163.3 | |  |
| **2015** |  | 147 | 11514 | 127.7 | 107.8 | 150.1 | |  | | 126 | 7903 | 159.4 | 133 | 189.5 |  | 72 | 4856 | 148.3 | 116.2 | 186.4 | |  |
| **2016** |  | 120 | 8580 | 139.9 | 116.1 | 167.1 | |  | | 102 | 5954 | 171.3 | 140 | 207.4 |  | 47 | 3589 | 131 | 96.5 | 173.5 | |  |
| **2017°** |  | 14 | 1194 | 117.3 | 64 | 196.6 | |  | | 9 | 61 | 1475.4 | 692.2 | 2628.4 |  | # | # | # | # | # | |  |
| **Age at pregnancy start date** | | | | | | | | | | | | | | | | | | | | |  |  |
| **18–24** |  | 774 | 64282 | 120.4 | 112.1 | 129.1 | |  | | 675 | 40102 | 168.3 | 156 | 181.3 |  | 364 | 25770 | 141.2 | 127.3 | 156.3 | |  |
| **25–29** |  | 866 | 73199 | 118.3 | 110.5 | 126.5 | |  | | 763 | 54429 | 140.2 | 130.5 | 150.4 |  | 463 | 35746 | 129.5 | 118 | 141.8 | |  |
| **30–34** |  | 993 | 85923 | 115.6 | 108.4 | 123 | |  | | 854 | 65489 | 130.4 | 121.8 | 139.5 |  | 534 | 44713 | 119.4 | 109.5 | 130 | |  |
| **35–39** |  | 581 | 56947 | 102 | 93.8 | 110.7 | |  | | 501 | 39366 | 127.3 | 116.4 | 138.8 |  | 293 | 26076 | 112.4 | 99.9 | 125.9 | |  |
| **40–45** |  | 148 | 17804 | 83.1 | 70.1 | 97.9 | |  | | 125 | 8942 | 139.8 | 116.5 | 166.3 |  | 76 | 5627 | 135.1 | 106.5 | 168.9 | |  |
| **Ethnicity** | | | | | | | | | | | | | | | | | | | | |  |  |
| **White** |  | 2780 | 252166 | 110.2 | 106.1 | 114.5 | |  | | 2452 | 178527 | 137.3 | 132 | 142.9 |  | 1395 | 115810 | 120.5 | 114.2 | 127 | |  |
| **Asian** |  | 289 | 16757 | 172.5 | 153.1 | 193.6 | |  | | 230 | 12054 | 190.8 | 167.2 | 216.8 |  | 178 | 8918 | 199.6 | 171.2 | 231.3 | |  |
| **Black** |  | 116 | 8821 | 131.5 | 108.4 | 158 | |  | | 92 | 5672 | 162.2 | 130.3 | 199.5 |  | 60 | 4071 | 147.4 | 111.8 | 190.6 | |  |
| **Mixed** |  | 48 | 3528 | 136.1 | 98.7 | 182.8 | |  | | 42 | 2320 | 181 | 130 | 245.2 |  | 20 | 1518 | 131.8 | 80.1 | 203.8 | |  |
| **Other** |  | 81 | 6277 | 129 | 102.8 | 159.9 | |  | | 61 | 4360 | 139.9 | 107.1 | 179.5 |  | 43 | 3342 | 128.7 | 93.7 | 172.3 | |  |
| **Unknown** |  | 48 | 10606 | 45.3 | 33.6 | 59.6 | |  | | 41 | 5395 | 76 | 54.6 | 102.9 |  | 34 | 4273 | 79.6 | 55.5 | 110.5 | |  |
| **Index of Multiple Deprivation** | | | | | | | | | | | | | | | | | | | | |  |  |
| **1 (least)** |  | 564 | 61945 | 91 | 83.6 | 99 | |  | | 486 | 44577 | 109 | 99.4 | 119.3 |  | 316 | 30724 | 102.9 | 91.7 | 115 | |  |
| **2** |  | 620 | 58360 | 106.2 | 98 | 115 | |  | | 526 | 41240 | 127.5 | 116.9 | 138.9 |  | 311 | 27772 | 112 | 99.8 | 125.2 | |  |
| **3** |  | 674 | 58758 | 114.7 | 106.1 | 123.8 | |  | | 585 | 40881 | 143.1 | 131.7 | 155.2 |  | 359 | 27328 | 131.4 | 118.1 | 145.8 | |  |
| **4** |  | 724 | 59246 | 122.2 | 113.5 | 131.4 | |  | | 624 | 40908 | 152.5 | 140.9 | 164.9 |  | 345 | 26604 | 129.7 | 116.3 | 144.1 | |  |
| **5 (most)** |  | 775 | 59612 | 130 | 120.9 | 139.6 | |  | | 692 | 40573 | 170.6 | 158.1 | 183.7 |  | 395 | 25415 | 155.4 | 140.5 | 171.4 | |  |
| **Missing** |  | 5 | 234 | 213.7 | 64.3 | 513 | |  | | 5 | 149 | 335.6 | 106.4 | 778.6 |  | *#* | *#* | *#* | *#* | *#* | |  |

AP, All Pregnancies; AP24+, All Pregnancies with gestational age ≥24 weeks; LR, Low-Risk pregnancies; n, number of pregnancies in the specified category; CI, confidence interval; #, results where an individual cell count is <5 (to maintain confidentiality).

**Table S9.25. Fetal/perinatal distress or asphyxia**

|  |  | **AP cohort** | | | | |  | | **AP24+ cohort** | | | | | |  | **LR cohort** | | | | | |  |
| --- | --- | --- | --- | --- | --- | --- | --- | --- | --- | --- | --- | --- | --- | --- | --- | --- | --- | --- | --- | --- | --- | --- |
|  |  | **n** | **Population under study** | **Incidence/**  **10 000** | **95% CI** | | |  | | **n** | **Population under study** | **Incidence/**  **10 000** | **95% CI** | |  | **n** | **Population under study** | **Incidence/**  **10 000** | **95% CI** | | |  |
| **Year of pregnancy start date** | | | | | | | | | | | | | | | | | | | | |  |  |
| **2004*** |  | 2186 | 16027 | 1363.9 | 1311.2 | 1418.1 | |  | | 2143 | 14041 | 1526.2 | 1467.1 | 1586.8 |  | 1510 | 10040 | 1504 | 1434.6 | 1575.4 | |  |
| **2005** |  | 3113 | 27609 | 1127.5 | 1090.2 | 1165.7 | |  | | 3030 | 19220 | 1576.5 | 1525.2 | 1628.8 |  | 2156 | 13456 | 1602.3 | 1540.6 | 1665.4 | |  |
| **2006** |  | 3399 | 28221 | 1204.4 | 1166.4 | 1243.2 | |  | | 3299 | 19706 | 1674.1 | 1622.2 | 1727 |  | 2290 | 13527 | 1692.9 | 1630 | 1757.2 | |  |
| **2007** |  | 3555 | 29076 | 1222.7 | 1185 | 1261.1 | |  | | 3455 | 20268 | 1704.7 | 1653.1 | 1757.2 |  | 2385 | 13830 | 1724.5 | 1661.9 | 1788.5 | |  |
| **2008** |  | 3608 | 29761 | 1212.3 | 1175.1 | 1250.3 | |  | | 3463 | 20382 | 1699 | 1647.7 | 1751.3 |  | 2374 | 13709 | 1731.7 | 1668.7 | 1796.1 | |  |
| **2009** |  | 3819 | 29342 | 1301.5 | 1263 | 1340.9 | |  | | 3699 | 20125 | 1838 | 1784.7 | 1892.2 |  | 2470 | 13421 | 1840.4 | 1775.2 | 1907 | |  |
| **2010** |  | 4004 | 27983 | 1430.9 | 1389.8 | 1472.7 | |  | | 3883 | 19479 | 1993.4 | 1937.5 | 2050.2 |  | 2522 | 12763 | 1976 | 1907.2 | 2046.2 | |  |
| **2011** |  | 3745 | 27209 | 1376.4 | 1335.4 | 1418.2 | |  | | 3623 | 19073 | 1899.5 | 1844.1 | 1956 |  | 2361 | 12349 | 1911.9 | 1842.8 | 1982.4 | |  |
| **2012** |  | 3479 | 24661 | 1410.7 | 1367.2 | 1455.1 | |  | | 3325 | 16888 | 1968.9 | 1909.1 | 2029.7 |  | 2139 | 10688 | 2001.3 | 1925.8 | 2078.5 | |  |
| **2013** |  | 3099 | 20812 | 1489 | 1440.5 | 1538.6 | |  | | 2975 | 14269 | 2084.9 | 2018.5 | 2152.5 |  | 1893 | 8982 | 2107.5 | 2023.6 | 2193.4 | |  |
| **2014** |  | 2306 | 16166 | 1426.5 | 1372.4 | 1481.8 | |  | | 2210 | 10959 | 2016.6 | 1941.8 | 2093 |  | 1392 | 6697 | 2078.5 | 1981.9 | 2177.7 | |  |
| **2015** |  | 1717 | 11514 | 1491.2 | 1426.4 | 1557.9 | |  | | 1663 | 7903 | 2104.3 | 2014.8 | 2195.8 |  | 1027 | 4856 | 2114.9 | 2000.8 | 2232.5 | |  |
| **2016** |  | 1266 | 8580 | 1475.5 | 1400.5 | 1553 | |  | | 1227 | 5954 | 2060.8 | 1958.6 | 2165.8 |  | 763 | 3589 | 2125.9 | 1993 | 2263.5 | |  |
| **2017°** |  | 23 | 1194 | 192.6 | 122.1 | 288.4 | |  | | 11 | 61 | 1803.3 | 929.9 | 3009.6 |  | *#* | *#* | *#* | *#* | *#* | |  |
| **Age at pregnancy start date** | | | | | | | | | | | | | | | | | | | | |  |  |
| **18–24** |  | 8001 | 64282 | 1244.7 | 1218.6 | 1271.1 | |  | | 7681 | 40102 | 1915.4 | 1876.2 | 1955 |  | 5006 | 25770 | 1942.6 | 1893.6 | 1992.3 | |  |
| **25–29** |  | 10315 | 73199 | 1409.2 | 1383.4 | 1435.3 | |  | | 9962 | 54429 | 1830.3 | 1797.4 | 1863.5 |  | 6575 | 35746 | 1839.4 | 1798.8 | 1880.5 | |  |
| **30–34** |  | 12241 | 85923 | 1424.6 | 1400.9 | 1448.7 | |  | | 11845 | 65489 | 1808.7 | 1779.1 | 1838.6 |  | 8108 | 44713 | 1813.3 | 1777.6 | 1849.5 | |  |
| **35–39** |  | 7204 | 56947 | 1265 | 1237.3 | 1293.2 | |  | | 7002 | 39366 | 1778.7 | 1740.9 | 1817 |  | 4631 | 26076 | 1776 | 1729.6 | 1823 | |  |
| **40–45** |  | 1558 | 17804 | 875.1 | 833.1 | 918.5 | |  | | 1516 | 8942 | 1695.4 | 1618.3 | 1774.6 |  | 963 | 5627 | 1711.4 | 1613.9 | 1812.3 | |  |
| **Ethnicity** | | | | | | | | | | | | | | | | | | | | |  |  |
| **White** |  | 33351 | 252166 | 1322.6 | 1308.9 | 1336.3 | |  | | 32282 | 178527 | 1808.2 | 1790.1 | 1826.5 |  | 21032 | 115810 | 1816.1 | 1793.5 | 1838.8 | |  |
| **Asian** |  | 2436 | 16757 | 1453.7 | 1398.2 | 1510.6 | |  | | 2328 | 12054 | 1931.3 | 1859.3 | 2004.9 |  | 1737 | 8918 | 1947.7 | 1863.8 | 2033.8 | |  |
| **Black** |  | 1318 | 8821 | 1494.2 | 1417.9 | 1572.9 | |  | | 1260 | 5672 | 2221.4 | 2112 | 2333.9 |  | 936 | 4071 | 2299.2 | 2168.7 | 2433.7 | |  |
| **Mixed** |  | 464 | 3528 | 1315.2 | 1202.6 | 1434.3 | |  | | 454 | 2320 | 1956.9 | 1795.6 | 2126 |  | 295 | 1518 | 1943.3 | 1744.6 | 2154.3 | |  |
| **Other** |  | 834 | 6277 | 1328.7 | 1243.5 | 1417.4 | |  | | 790 | 4360 | 1811.9 | 1697 | 1931.2 |  | 589 | 3342 | 1762.4 | 1633 | 1897.6 | |  |
| **Unknown** |  | 916 | 10606 | 863.7 | 811 | 918.6 | |  | | 892 | 5395 | 1653.4 | 1554.7 | 1755.7 |  | 694 | 4273 | 1624.2 | 1514 | 1739 | |  |
| **Index of Multiple Deprivation** | | | | | | | | | | | | | | | | | | | | |  |  |
| **1 (least)** |  | 8197 | 61945 | 1323.3 | 1296 | 1350.9 | |  | | 7992 | 44577 | 1792.9 | 1756.8 | 1829.3 |  | 5512 | 30724 | 1794 | 1750.6 | 1838.1 | |  |
| **2** |  | 7804 | 58360 | 1337.2 | 1308.9 | 1365.9 | |  | | 7499 | 41240 | 1818.4 | 1780.6 | 1856.6 |  | 5103 | 27772 | 1837.5 | 1791.4 | 1884.2 | |  |
| **3** |  | 7842 | 58758 | 1334.6 | 1306.3 | 1363.3 | |  | | 7595 | 40881 | 1857.8 | 1819.4 | 1896.7 |  | 5143 | 27328 | 1882 | 1834.9 | 1929.7 | |  |
| **4** |  | 7982 | 59246 | 1347.3 | 1319 | 1375.9 | |  | | 7714 | 40908 | 1885.7 | 1847.2 | 1924.7 |  | 5028 | 26604 | 1889.9 | 1842.4 | 1938.2 | |  |
| **5 (most)** |  | 7468 | 59612 | 1252.8 | 1225.2 | 1280.7 | |  | | 7181 | 40573 | 1769.9 | 1731.8 | 1808.5 |  | 4483 | 25415 | 1763.9 | 1716 | 1812.6 | |  |
| **Missing** |  | 26 | 234 | 1111.1 | 737.3 | 1588 | |  | | 25 | 149 | 1677.9 | 1124.4 | 2363.9 |  | 14 | 89 | 1573 | 895.9 | 2483.2 | |  |

AP, All Pregnancies; AP24+, All Pregnancies with gestational age ≥24 weeks; LR, Low-Risk pregnancies; n, number of pregnancies in the specified category; CI, confidence interval; #, results where an individual cell count is <5 (to maintain confidentiality).

If a particular pregnancy-related event of interest occurred several times for the same pregnancy, it was only counted once for that pregnancy.
